# Durum Wheat Stress Tolerance Induced by Endophyte *Pantoea agglomerans* with Genes Contributing to Plant Functions and Secondary Metabolite Arsenal

**DOI:** 10.3390/ijms20163989

**Published:** 2019-08-16

**Authors:** Hafsa Cherif-Silini, Bathini Thissera, Ali Chenari Bouket, Nora Saadaoui, Allaoua Silini, Manal Eshelli, Faizah N. Alenezi, Armelle Vallat, Lenka Luptakova, Bilal Yahiaoui, Semcheddine Cherrad, Sebastien Vacher, Mostafa E. Rateb, Lassaad Belbahri

**Affiliations:** 1 Laboratory of Applied Microbiology, Department of Microbiology, Faculty of Natural and Life Sciences, Ferhat Abbas University, Setif 19000, Algeria; 2 School of Computing, Engineering and Physical Sciences, University of the West of Scotland, PA12BE Paisley, UK; 3 Plant Protection Research Department, East Azarbaijan Agricultural and Natural Resources Research and Education Center, AREEO, Tabriz 5355179854, Iran; 4 Food Science and Technology Department, Faculty of Agriculture, University of Tripoli, Tripoli 13275, Libya; 5 NextBiotech, 98 Rue Ali Belhouane, Agareb 3030, Tunisia; 6 Neuchâtel Platform of Analytical Chemistry, Institute of Chemistry, University of Neuchâtel, 2000 Neuchâtel, Switzerland; 7 Department of Biology and Genetics, Institute of Biology, University of Veterinary Medicine and Pharmacy, Zoology and Radiobiology, Komenského, 04181 Kosice, Slovakia; 8 CONIPHY, Parc d’activitésen Chuel, Route de Chasselay, 69650 Quincieux, France; 9 Laboratory of Soil Biology, University of Neuchatel, 2000 Neuchatel, Switzerland

**Keywords:** abiotic stress, antimicrobial activity, durum wheat, PGPR, *Pantoea agglomerans*, secondary metabolites, OSMAC, metabolomics, dereplication, wheat microbiome

## Abstract

In the arid region Bou-Saâda at the South of Algeria, durum wheat *Triticum durum* L. cv Waha production is severely threatened by abiotic stresses, mainly drought and salinity. Plant growth-promoting rhizobacteria (PGPR) hold promising prospects towards sustainable and environmentally-friendly agriculture. Using habitat-adapted symbiosis strategy, the PGPR *Pantoea agglomerans* strain Pa was recovered from wheat roots sampled in Bou-Saâda, conferred alleviation of salt stress in durum wheat plants and allowed considerable growth in this unhostile environment. Strain Pa showed growth up to 35 °C temperature, 5–10 pH range, and up to 30% polyethylene glycol (PEG), as well as 1 M salt concentration tolerance. Pa strain displayed pertinent plant growth promotion (PGP) features (direct and indirect) such as hormone auxin biosynthesis, production of 1-aminocyclopropane-1-carboxylate (ACC) deaminase, and ammonia and phosphate solubilization. PGPR features were stable over wide salt concentrations (0–400 mM). Pa strain was also able to survive in seeds, in the non-sterile and sterile wheat rhizosphere, and was shown to have an endophytic life style. Phylogenomic analysis of strain Pa indicated that *Pantoea* genus suffers taxonomic imprecision which blurs species delimitation and may have impacted their practical use as biofertilizers. When applied to plants, strain Pa promoted considerable growth of wheat seedlings, high chlorophyll content, lower accumulation of proline, and favored K^+^ accumulation in the inoculated plants when compared to Na^+^ in control non-inoculated plants. Metabolomic profiling of strain Pa under one strain many compounds (OSMAC) conditions revealed a wide diversity of secondary metabolites (SM) with interesting salt stress alleviation and PGP activities. All these findings strongly promote the implementation of *Pantoea agglomerans* strain Pa as an efficient biofertilizer in wheat plants culture in arid and salinity-impacted regions.

## 1. Introduction

Arid and semi-arid environments cover a considerable proportion of the earth landscape. One example is the south of Algeria, where water availability reaches the threshold that dramatically limits ecosystem functioning [1,2]. Irrigation is an ancient agricultural practice to favor crop production. However, in these areas where the climate is hot and dry, substantial water losses from irrigated lands occur through evapotranspiration [3]. Therefore, salts associated with irrigation water remain in the soil, and their concentration increases to levels causing a considerable reduction in the crop yield [4]. This process is called salinization and may lead to land abandonment [5]. Several studies have targeted understanding of the mechanisms behind plant stress tolerance (mainly drought and salinity) [2]. Plant response to salinity, heat, and drought abiotic stresses are complex and imply numerous reactions at the physiologic and genetic levels [2,6,7]. Different plants react in different ways, involving osmolyte production such as proline [8] and efficient reactive oxygen species scavenging (ROS) [9,10]. However, in extreme habitats where plants are exposed to important levels of abiotic stresses, relatively few species can thrive in such environments. To explain this observation, the habitat-adapted symbiosis ecological concept has been proposed. In the frame of this concept, microbiome from these extreme locations confer to plants habitat-specific stress tolerance [7]. This ecological concept was recently validated for numerous species from different extreme ecosystems such as rice tolerance to salt or drought stress [11], the halophyte *Limonium sinense* adaptation to salt stress [12], and *Phragmites australis* tolerance to high saline habitats [13].

Plant growth-promoting rhizobacteria (PGPR) are attracting considerably more attention owing to their remarkable and beneficial effects on plants [14] and their possible replacement of chemical plant protection, which is highly damaging to the environment [15]. They have proven effective in conferring tolerance against abiotic [16,17] and biotic stresses [18,19,20,21]. Plant benefits include direct plant growth promotion (PGP) activities such as phytohormone production, phosphate solubilization, and nitrogen fixation [22], as well as indirect PGP activities such as hydrogen cyanide (HCN), exoenzyme and 1-aminocyclopropane-1-carboxylate (ACC) deaminase production [23]. PGPR are extensively recovered from extreme niches such as arid regions [23] and salty environments [14]. Genome mining is an efficient way in the field of PGPR for two major reasons: (1) Phylogenomic analysis is now accepted as the gold standard for bacterial classification, rather than classical 16S rRNA phylogeny [22,24,25]; and (2) genome mining provides a direct link to bacterial genes contributing to plant-beneficial functions and allows efficient comparative genomic analysis of inter- and intra-species or genera of PGPR isolates through homology-based mining [22]. Additionally, genome mining provides accessibility to the putative secondary metabolite clusters of the targeted strains [24,25,26].

In the current study, we have recovered *Pantoea agglomerans* strain Pa from the durum wheat rhizosphere collected from the arid region Bou-Saâda (South of Algeria). Strain Pa proved effective in alleviating salt stress in durum wheat plants. It was able to survive in wheat seeds, sterile wheat rhizosphere and compete with other microorganisms in normal wheat rhizosphere. Genome mining of strain Pa showed a large repertoire of genes contributing to plant beneficial functions harbored by the *Pantoea* core genome and a wide range of secondary metabolites harbored by the accessory Pa genome. Additionally, the identification of different secondary metabolites strain Pa produce would be of great value to elaborate PGP ability of Pa as well as its ability to get involved in other biological effects. This was achieved by metabolomic profiling of strain Pa under one strain many compounds (OSMAC) conditions which revealed a high diversity of secondary metabolites with interesting therapeutic and PGP activities.

## 2. Results

### 2.1. P. agglomerans Pa Strain Recovery

Rhizosphere samples of durum wheat collected from durum wheat fields in the arid Bou-Saâda region were used for the isolation of different strains and their screening for PGP activities. Primary selection identified 22 strains that have been submitted to a secondary screen for PGP activities with high tolerance to salt. Pa strain was then selected as the best bacterial isolate with high PGP activities in the initial testing, the highest resistance to salt stress and the highest PGP activities under salt stress, and used in downstream experiments.

### 2.2. P. agglomerans Strain Pa Abiotic Stress Tolerance

**Figure 1:**
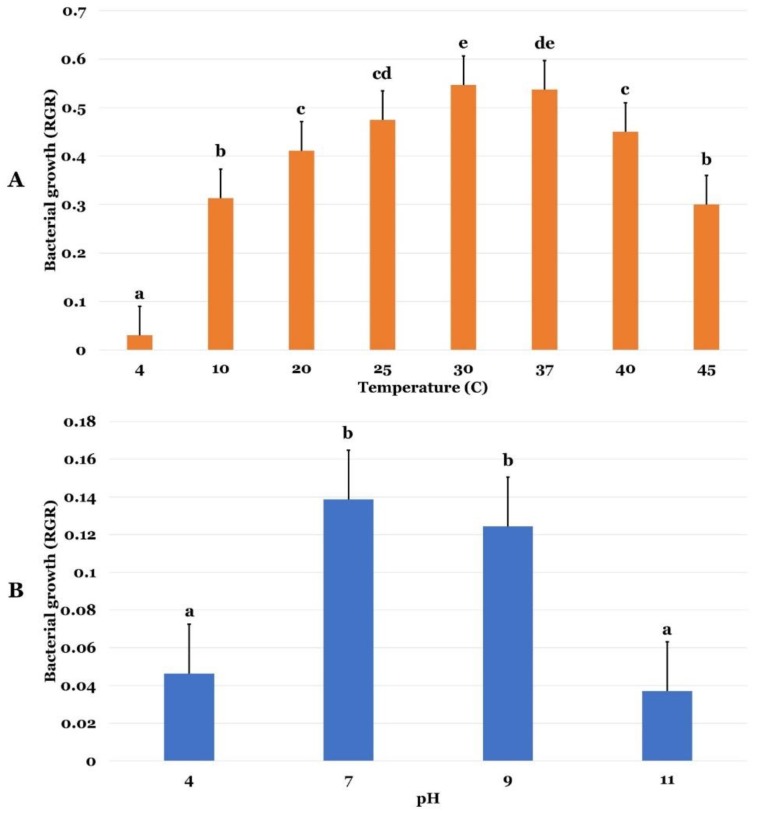
Growth performance of *P. agglomerans* strain Pa to temperature and pH stresses. (**A**) Temperature effect on relative growth rate (RGR) of bacterium. (**B**) pH effect on RGR bacterial growth. Data present the mean of three repetitions ± standard error. One-way ANOVA was performed by using SPSS 22 and subsequent Tukey’s multiple comparison post-test. Points highlighted with different letters indicate significant differences among the treatments (*p* < 0.05).

**Figure 2:**
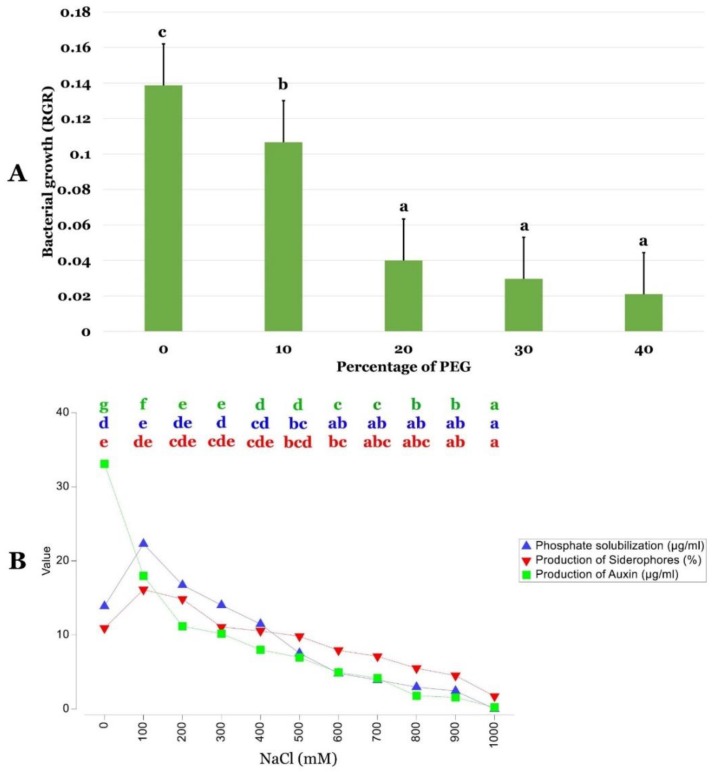
Growth performance of *P. agglomerans* strain Pa to polyethylene glycol (PEG) stress and its plant growth promoting (PGP) abilities and stabilities toward salt stress. (**A**) Effect of PEG percentage on bacterial growth (RGR). (**B**) Coherence chart illustrating the stability of PGP abilities of *P. agglomerans* strain Pa to varying NaCl concentrations. Data are represented by the mean of three repetitions ± standard error. One-way ANOVA was performed by using SPSS 22 and subsequent Tukey’s multiple comparison post-test. Points that are labeled with different letters indicate significantly different values (*p* < 0.05) among the treatments.

Three abiotic stresses were monitored in this study, namely temperature, pH, and osmotic stresses in *P. agglomerans* strain Pa. Figure 1A shows that optimal growth temperature of *P. agglomerans* strain Pa was 30 °C. The strain was also able to tolerate temperatures comprised between 10—40 °C (Figure 1A). Strain Pa grew best at pH 7 (Figure 1B). However, it was able to show moderate growth at 4.5 to 10 pH values. Statistical analysis showed no significant difference in strain Pa growth in pH values comprised between 7 and 9 (*p* < 0.05). Increasing concentrations of polyethylene glycol (PEG)clearly showed that *P. agglomerans* strain Pa grew well and tolerated PEG concentrations up to 30%. However, its growth was dropped beyond this value at higher PEG concentrations (Figure 2A). Statistical analysis showed clear statistical difference between 0 to 10% and 10 to 20% PEG concentrations. However, there was not a statistical difference among 20%, 30%, and 50% of PEG.

### 2.3. P. agglomerans Strain Pa PGP Activities

*P. agglomerans* strain Pa was able to perform direct plant growth promotion (Supplementary Table S3). It was able to solubilize phosphate, show growth on nitrogen-free media, and produce siderophore and plant hormone IAA (Figure 2B). Moreover, *P. agglomerans* strain Pa was able to perform indirect plant growth promotion, mainly chitinase, cellulase, and protease production. It was also able to produce ACC deaminase, ammonia NH_3_, and cyanhydric acid (HCN).

### 2.4. NaCl Stress Effect on PGP Activities of P. agglomerans Strain Pa

**Figure 3:**
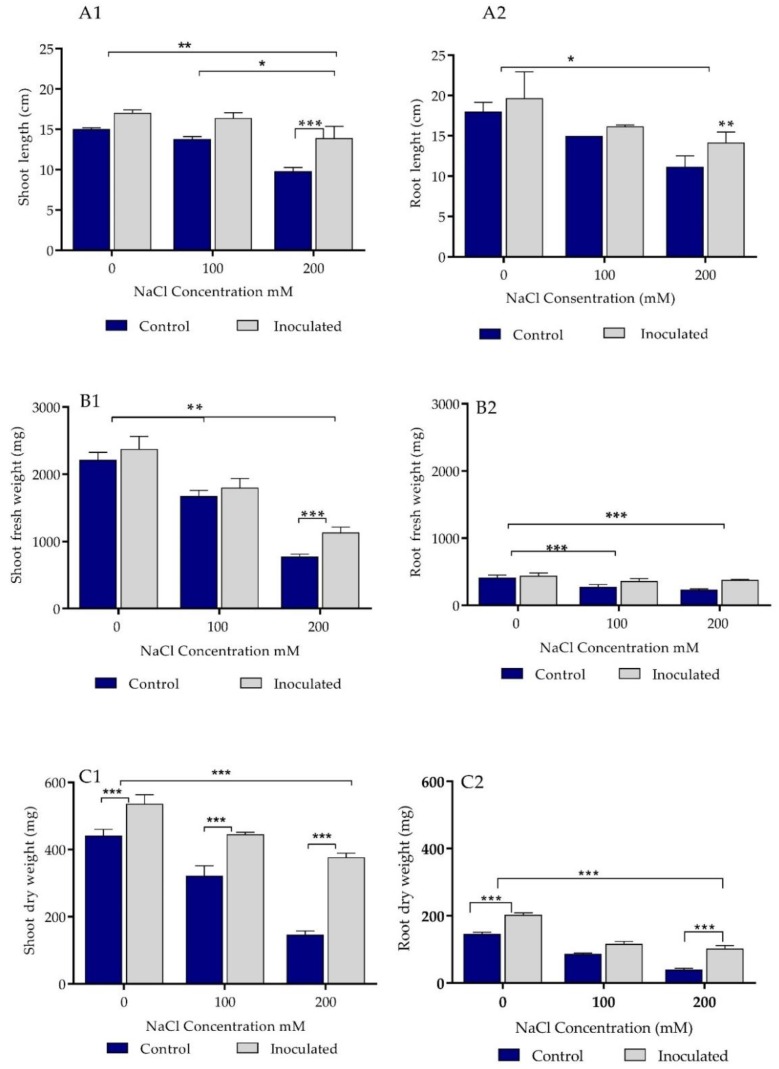
Effect of *P. agglomerans* strain Pa inoculation on growth parameters of durum wheat cv Waha grown in sand pots under saline and non-saline conditions. (**A1**,**A2**) Effect of bacterial inoculation (strain Pa) on shoot and root length (cm) in the presence of three concentrations (0, 100, and 200 mM) of NaCl. (**B1**,**B2**) Effect of bacterial inoculation (isolate Pa) on shoot and root fresh weight (mg) of *Triticum durum* L. cv Waha in the presence of three concentrations (0, 100, and 200 mM) of NaCl. (**C1**,**C2**) Effect of bacterial inoculation (strain Pa) on shoot and root dry weight (mg) of *Triticum durum* L. cv Waha in the presence of 0, 100, and 200 mM concentrations of NaCl. The bar plots represent the mean ± standard error of three different experiments. GraphPad Prism 8 was used to perform statistical analysis, using a two-way ANOVA and Tukey’s multiple comparison post-test. Only significant differences are displayed: * *p* < 0.05, ** *p* < 0.01, *** *p* < 0.001.

Figure 3B unambiguously shows that up to 1 M of varying salt concentrations interferes slightly with growth of Pa strain. For the varying salt concentrations, there was no significant difference of PGP activities (phosphate solubilization and siderophore production, for example) from control values up to 400 mM salt concentration (*p* < 0.05). However, at 100 mM salt concentration, IAA production was significantly reduced (*p* < 0.05).

### 2.5. Growth Parameters Analysis of Control and Bacteria Inoculated Durum Wheat Plants Grown in Sand Pots

Treatment of durum wheat seeds with *P. agglomerans* strain Pa showed significant positive effect on shoot length in both the absence of salt stress and at 100 mM salt (Figure 3(A1), Supplementary Figure S3). The shoot lengths were decreased by increasing the salt concentration. The shoot lengths were affected more by 200 mM salt concentration in comparison to the lower concentration. However, Pa had significant support for the shoot length in comparison to the control at 200 mM salt concentration (*p* ≤ 0.001).

Strain Pa enhanced shoots’ length growth even in the highest concentration used in this experiment. At 200 mM, strain Pa inoculation induced the growth of root lengths in comparison to the control (*p* ≤ 0.01, Figure 3(A2)). Strain Pa inoculation significantly affected (*p* ≤ 0.01) the fresh weight of durum wheat plants at 200 mM salt concentration (Figure 3(C1)). It also significantly affected the root fresh weight at 100 and 200 mM salt concentrations (Figure 3(C2)). Finally, inoculation of strain Pa significantly affected the dry weight of shoots and roots for all controls and treatments (100 and 200 mM salt concentrations, Figure 3(C2)).

### 2.6. Biochemical Parameters of Control and Bacterial Inoculated Durum Wheat Plants Grown in Sand Pots

**Figure 4:**
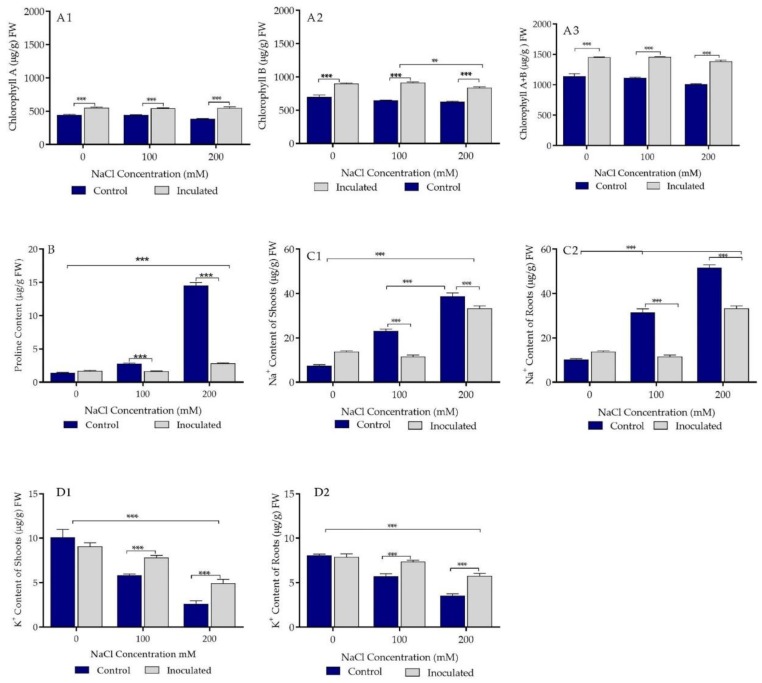
Effect of plant growth-promoting rhizobacteria (PGPR) *P. agglomerans* strain Pa inoculation on biochemical parameters of durum wheat cv Waha plants grown under saline and non-saline conditions in sand pots. (**A1**,**A2**,**A3**) Effect of bacterial inoculation (isolate Pa) on chlorophyll a, b, and a + b chlorophyll content (µg/g FW) in the presence of three concentrations (0, 100, and 200 mM) of NaCl. (**B**) Effect of bacterial inoculation (isolate Pa) on proline content (µg/g FW) under the presence of three concentrations (0, 100, and 200 mM) of NaCl. (**C1**,**C2**) Effect of bacterial inoculation (isolate Pa) on shoot and root Na+ content (µg/g FW). (**D1**,**D2**) Effect of bacterial inoculation (isolate Pa) on shoot and root K+ content (µg/g FW). The bar plots represent the mean ± standard error of three different experiments. GraphPad Prism 8 was used to perform statistical analysis, with a two-way ANOVA and Tukey’s multiple comparison post-test. Only significant variations are displayed ** *p* < 0.01, *** *p* < 0.001.

Three biochemical parameters were targeted in this study, namely chlorophyll, proline, and Na^+^ and K^+^ contents of control and Pa-treated durum wheat plants. Pa treatment significantly induced higher amounts of chlorophyll a, b, and a + b independently of the type of treatment (0, 100, and 200 mM salt concentrations, Figure 4(A1,A2,A3)). Treatment of durum wheat plants with either 100 or 200 mM salt concentrations significantly accumulated proline (Figure 4B). Pa inoculation suppressed proline accumulation in the plants (Figure 4B). Treatment of durum wheat plants with either 100 or 200 mM salt concentrations induced significant increase of Na^+^ and depletion of K^+^ concentrations in roots and shoots (Figure 4(C1,C2,D1,D2)). Pa inoculation strongly reduced Na^+^ and elevated K^+^ levels close to control values (Figure 4(C1,C2,D1,D2)).

### 2.7. Growth Parameters Analysis of Control and Bacterial Inoculated Durum Wheat Plants Grown in Soil Pots

**Figure 5:**
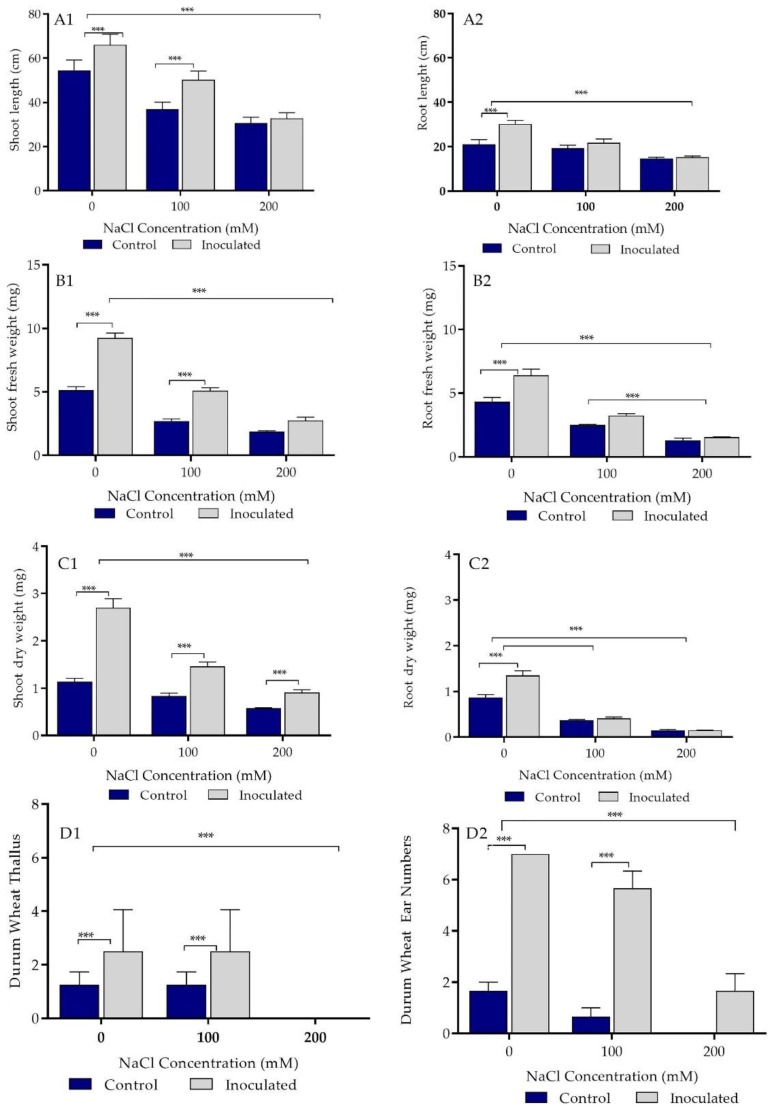
Effect of PGPR *P. agglomerans* strain Pa inoculation on growth parameters of durum wheat cv Waha grown in saline and non-saline settings in soil pots. (**A1**,**A2**) Effect of bacterial inoculation (strain Pa) on shoot and root length (cm) in the presence of three concentrations (0, 100, and 200 mM) of NaCl. (**B1**,**B2**) Effect of bacterial inoculation (strain Pa) on shoot and root fresh weight (mg) of *Triticum durum* L. cv Waha in the presence of three concentrations (0, 100, and 200 mM) of NaCl. (**C1**,**C2**) Effect of bacterial inoculation (isolate Pa) on shoot and root dry weight (mg) of *T. durum* L. cv Waha in the presence of three concentrations (0, 100, and 200 mM) of NaCl. (**D1**,**D2**) Effect of bacterial inoculation (strain Pa) on thallus and ear numbers of wheat plants grown in soil using three salt concentrations (0, 100, and 200 mM). Bar plots represent the mean ± standard error of three different experiments. We used GraphPad Prism 8 to make statistical analysis, using a two-way ANOVA setting followed by Tukey’s multiple comparison post-test. Only significant differences are displayed: *** *p* < 0.001.

Treatment of durum wheat seeds with *P. agglomerans* strain Pa significantly enhanced shoot and root length of in both absence of salt stress and at 100 mM salt concentration (Figure 5(A1,A2), Supplementary Figure S4). No significant support could be found for the longer durum wheat plants’ shoots and roots treated with strain Pa compared to the control at 200 mM (Figure 5(A1,A2)). Strain Pa inoculation significantly affected dry and fresh weights of durum wheat shoots and roots, except the fresh weight of roots at 200 mM salt and the roots’ dry weight at 100 and 200 mM salt (Figure 5B,C). Strain Pa inoculation had a significant effect on increasing the thallus numbers at 0 and 100 mM salt concentrations in comparison to control sample, while at 200 mM salt concentration, the thallus numbers disappeared in the control and treated sample. The number was decreased in plants irrigated with 0 and 100 mM salt. Statistically, the effect of the Pa strain on plants irrigated with 200 mM salt was significantly different (*p* ≤ 0.01) from 0 and 100 mM salt concentrations. However, it is important to note that Pa strain enhanced the ear numbers in comparison to the control in all concentrations.

### 2.8. Biochemical Parameters of Control and Bacterial Inoculated Durum Wheat Plants Grown in Soil Pots

**Figure 6:**
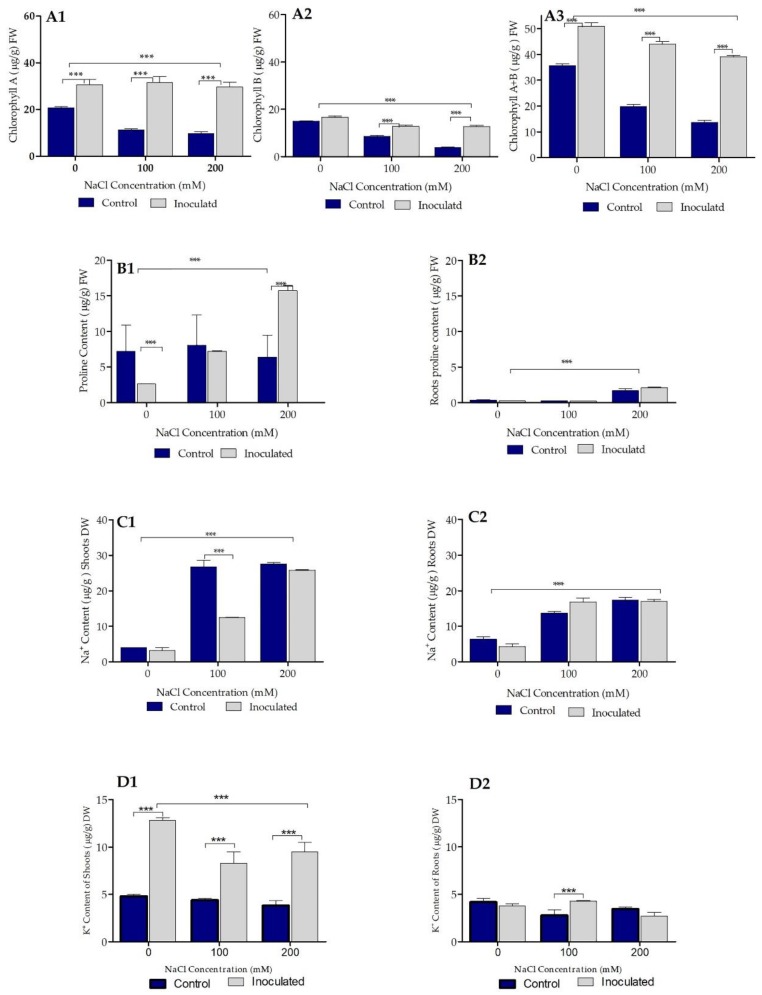
Effect of PGPR *P. agglomerans* strain Pa inoculation on biochemical parameters of durum wheat cv Waha grown in saline and non-saline settings in soil pots. (**A1**,**A2**,**A3**) Effect of bacterial inoculation (isolate Pa) on chlorophyll a, b, and a + b contents (µg/g FW) in wheat plants grown in soil with the presence of three NaCl concentrations (0, 100, and 200 mM). (**B1**,**B2**) Effect of bacterial inoculation (isolate Pa) on proline content (µg/g FW) in the presence of three concentrations (0, 100, and 200 mM) of NaCl in soil-grown wheat plants. (**C1**,**C2**) Effect of bacterial inoculation (isolate Pa) on Na^+^ content (µg/g DW) in the presence of three concentrations (0, 100, and 200 mM) of NaCl in wheat plants grown in soil. (**D1**,**D2**) Effect of Pa inoculation on K^+^ content (µg/g DW) in the presence of three NaCl concentrations (0, 100, and 200 mM) in wheat plants grown in soil. The bar plots represent the mean ± standard error of three different experiments. We used GraphPad Prism 8 to perform statistical analysis, using two-way ANOVA followed by Tukey’s multiple comparison post-test. Only significant variations are displayed: *** *p* < 0.001.

Chlorophyll, proline, and Na+ and K+ contents of control and Pa-treated durum wheat plants were targeted in this study. Similar to plants grown in sand pots, plants grown in soil pots showed significantly higher amounts of chlorophyll a, b, and a + b independently of the type of treatment (0, 100, and 200 mM salt concentrations, Figure 6(A1,A2,A3)). Treatment of durum wheat plants with either 100 or 200 mM salt concentrations significantly accumulated proline (Figure 6(B1,B2)). Pa-inoculated plants showed similar proline accumulation trends as in control plants (Figure 6(B1,B2)). For Na^+^ and K^+^ contents of control and Pa-treated durum wheat plants, a similar tendency was observed as for plants grown in sand.

### 2.9. Life Style of P. agglomerans Strain Pa

**Figure 7:**
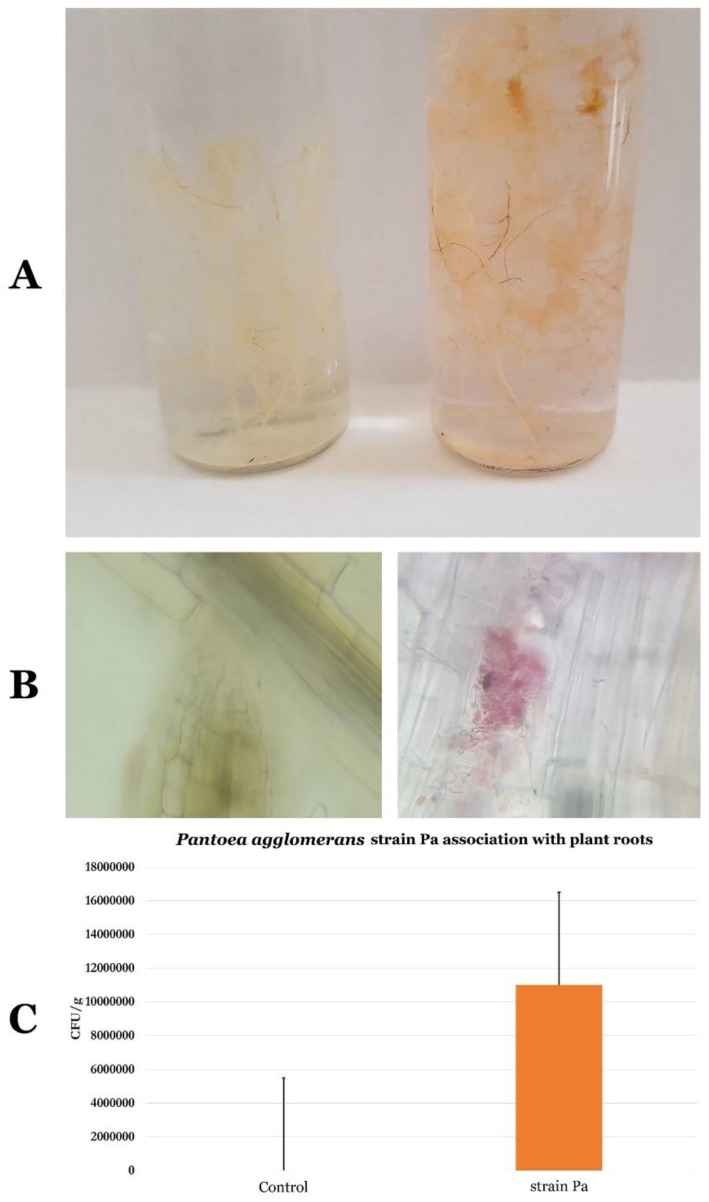
*P. agglomerans* strain Pa lifestyle. (**A**) Control plant roots (left) and *P. agglomerans* strain Pa adherence to plant roots (right). (**B**) Control plant roots (left) and *P. agglomerans* strain Pa staining within the plant root tissues (right). (**C**) Natural logarithm of colony-forming unit (Ln CFU) of *P. agglomerans* strain Pa association per gram of plant roots.

Microscopic examination of wheat roots incubated with bacterial suspension in the triphenyl tetrazolium chloride (TTC) assay indicated clear presence of rose areas at the surface of the roots (Figure 7A,B). This result attested for the presence of huge amounts of *P. agglomerans* strain Pa present at the wheat root surface. Analysis of wheat plant roots grown in the presence of *P. agglomerans* strain Pa allowed the quantification of up to 1.2 × 10^7^ colony-forming unit (CFU)/g inside the wheat plant roots (Figure 7C).

### 2.10. Survival of P. agglomerans Strain Pa in Wheat Seeds and in Non-Sterile and Sterile Wheat Rhizosphere

**Figure 8:**
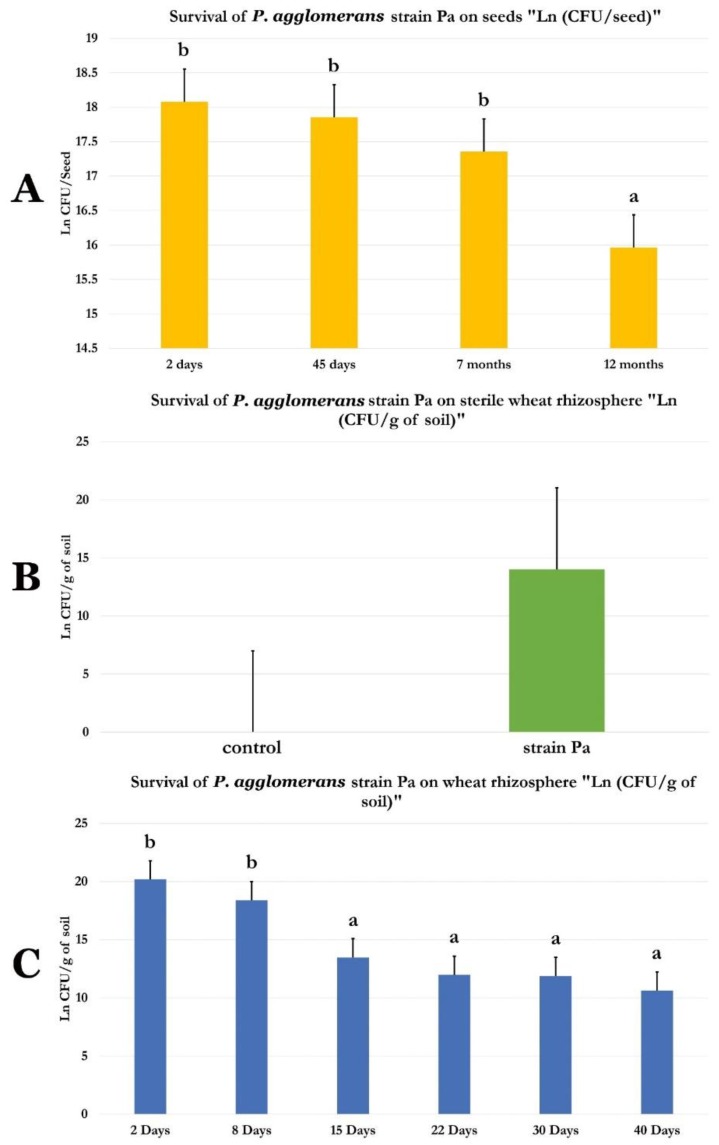
Survival of *P. agglomerans* strain Pa on (**A**) seeds, (**B**) sterile wheat rhizosphere, and (**C**) non-sterile wheat rhizosphere.

*P. agglomerans* strain Pa encapsulated in carboxy methyl cellulose (CMC) on wheat seeds, subsequent bacterial recovery, and viability check indicate that *P. agglomerans* strain Pa could be recovered from seeds at 7 × 10^7^, 5.8 × 10^7^, 3.6 × 10^7^, and 0.95 × 10^7^ CFU/seed. Wheat seeds encapsulated with *P. agglomerans* strain Pa and germinated in sterile compost substrate indicate that *P. agglomerans* strain Pa could be recovered at a rate of 1.2 × 10^6^ CFU/g of soil after 45 days of culture. When *P. agglomerans* strain Pa were germinated in non-sterile compost substrate, *P. agglomerans* strain Pa could be recovered at a rate of 2 × 10^8^, 2 × 10^7^, 2 × 10^6^, 1.6 × 10^5^, 1.6 × 10^5^, and 5 × 10^4^ after 2, 8, 15, 22, 30, and 40 days of culture, respectively (Figure 8A–C).

### 2.11. Phylogenetic Affinities of Strain Pa

Genomes of *P. vagans* and *P. agglomerans* strains collections available in GenBank were selected for phylogenomic analysis and the exact phylogenetic position of *P. agglomerans* strain Pa was inferred (Table 1). The genome size was in the range of 3.52 to 5.36 mega base pairs (Supplementary Table S1). Genome-to-Genome Distance Calculator (GGDC) analysis revealed four different species sensu Meier-Kolthoff et al. [27] that was nominated *P. vagans* and *P. agglomerans*. Species sensu Meier-Kolthoff et al. [27] are defined based on the presence of 70% similarity between two genomes, and this cut-off is believed to be suitable for bacterial species discrimination and is considered as the standard threshold allowing species boundaries delimitation (Supplementary Figure S1A).

**Figure 9:**
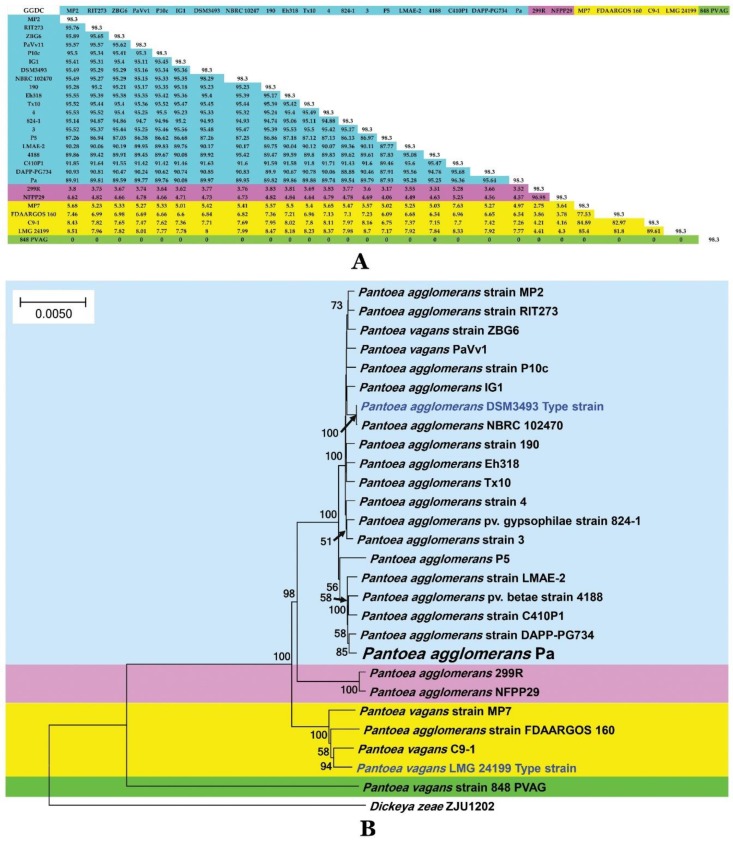
Taxonomy of *P. agglomerans* Pa isolate and its phylogenetic position within fully-sequenced genomes of *P. vagans* and *P. agglomerans* isolates publicly available in GenBank database. (**A**) Genome-to-Genome Distance Calculation (GGDC) values between each indicated strain were calculated with GGDC 2 web-based program, showing four species candidates based on 70% similarity thresholds. (**B**) Maximum Likelihood phylogenomic tree of Gram-negative bacteria *P. agglomerans* and *P. vagans* strains. *Dickeya zeae* strain ZJU1202 was used as outgroup. Bar, number of expected changes per site.

Average nucleotide identity (ANI) analysis confirmed the observations drawn using GGDC and four distinct species [38] were unambiguously documented (Supplementary Figure S1B). Strain Pa was therefore identified as *P. agglomerans,* while the group of strains MP7, C9-1, FDAARGOS 160 belongs to *P. vagans* and two new species represented by strains (848 PVAG) and (299R and NFPP 29) have to be erected. GGDC values (Figure 9A) allowed to note a total agreement for species delimitation. Whole genome phylogeny showed clear discrimination of the four putative groups within the *P. agglomerans–P. vagans* group (Figure 9B).

### 2.12. P. vagans and P. agglomerans Genome Mining

#### 2.12.1. PGP Activities

**Figure 10:**
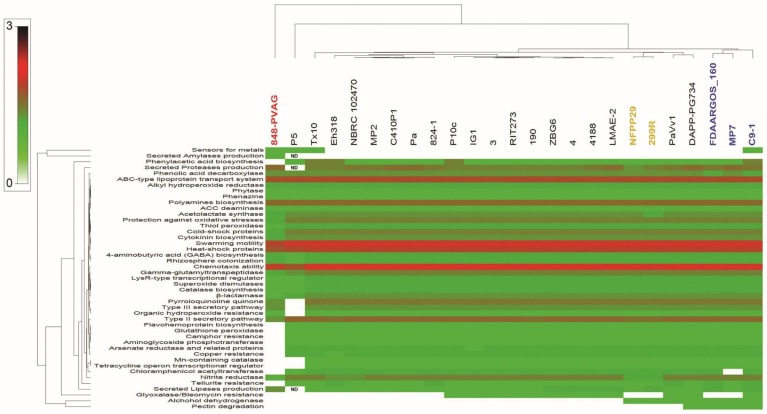
Genome mining of plant-beneficial function genes in *Pantoea agglomerans* and *P. vagans* strains illustrated by a heat map. Same colors are used for labeling strains of the same species.

Genome mining of the 25 *Pantoea* strains for PGP activities was conducted through homology-based searches for genes with putative functions that could benefit plants. Genes with plant beneficial functions involve: (i) Genes for nutrient recovery, mainly nitrogen fixation, mineral phosphate solubilization, phytase production, urea utilization, and exoenzyme production; (ii) genes allowing bacterial fitness; (iii) genes allowing colonization of root and growth promotion such as swarming motility, chemotaxis ability, rhizosphere colonization, and exopolysaccharide biosynthesis; (iv) traits allowing plant growth such as hormones, mainly auxin, cytokinin and phenylacetic acid biosynthesis, ACC deamination, nitric oxide production, and polyamines biosynthesis; (v) oxidative stress plant-protecting genes such as antioxidant enzymes; (vi) disease resistance induction in plants, mainly salicylate, acetoin, and 2,3-butanediol biosynthesis; (vii) antibiotics and related compounds such as hydrogen cyanide, 2,4-diacetyl phloroglucinol production, and γ-aminobutyric acid biosynthesis; (viii) drugs and heavy metals resistance, mainly tetracycline, streptothricin, fosfomycin, bleomycin, penicillin, quinolone, chloramphenicol/florfenicol aminoglycosides, arsenic, copper, aluminium, camphor, tellurite, cadmium, cobalt, and zinc resistance; and (iv) degradation of aromatic compounds, mainly phenolic acids, sulfur-containing organic molecules, 2-methyl hydroquinone, and catechol resistance. Results indicate that the large majority of strains harbor huge PGP capacities (Figure 10). The majority of *P. agglomerans* and *P. vagans* strains collection showed that presence of mined genes was independent of their putative association to plant rhizosphere or genome coverage (Supplementary Table S1).

#### 2.12.2. Secondary Metabolite Clusters

**Figure 11:**
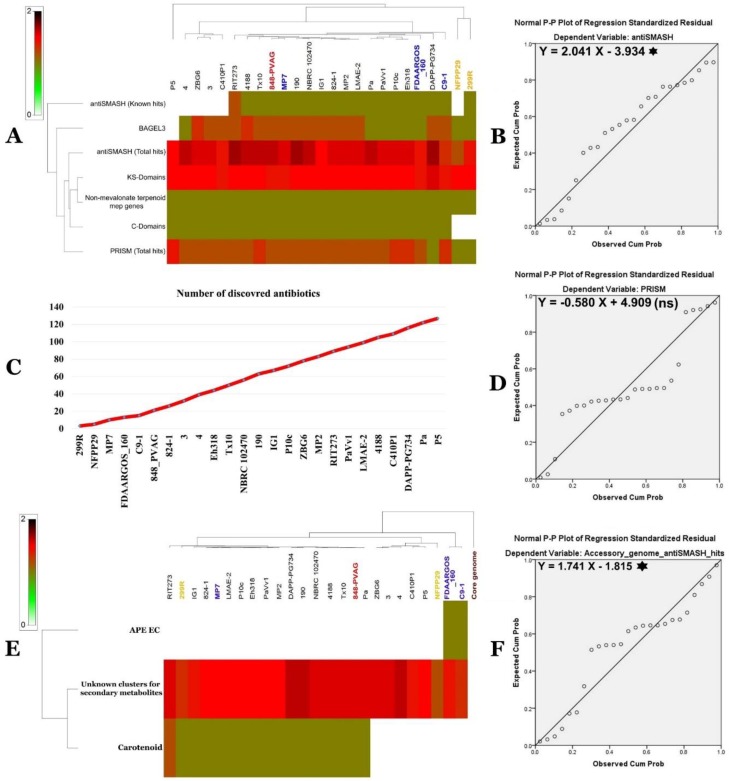
Secondary metabolite arsenal of *P. agglomerans* and *P. vagans* isolates and its relationship to genome size and accessory genomes. (**A**) Genome mining of secondary metabolite clusters illustrated by a heat map. (**B**) Linear relationship between antiSMASH total hits and genome sizes (*: statistically significant at *p* < 0.05). (**C**) Evolution of the number of discovered secondary metabolites in relation to the number of genomes sequenced. (**D**) Linear relationship between PRISM total hits and genome sizes (statistically non-significant). (**E**) Genome mining of secondary metabolite clusters in *P. agglomerans* and *P. vagans* accessory genomes illustrated by a heat map. (**F**) Linear relationship between antiSMASH total hits and sizes of the accessory genomes (*: statistically significant at *p* < 0.05).

Programs such as antiSMASH 3.0 [39], prediction informatics for secondary metabolomes (PRISM) [40], NapDos [41], NP.search [42], and BAGEL3 bacteriocin specific software [43] were used to identify secondary metabolite clusters present in the *P. agglomerans* and *P. vagans* genome collection. Figure 11A and Supplementary Table S5 show various secondary metabolite clusters using all approaches. Apart from carotenoid and arylpolyene biosynthetic gene clusters, none of the remaining clusters matched already-identified secondary metabolites, suggesting discovery of yet-to-be characterized natural products. Identified secondary metabolite clusters analyzed by the rarefaction approach highlight that saturation was far from being attained (Figure 11C).

All remaining secondary metabolites (SM) were unknown and lied within the different strain’s accessory genome. Using antiSMASH, a clear correspondence between the number of secondary metabolite gene clusters and the size of the accessory genome was demonstrated (Figure 11F). Approximately 17.7% of the number of secondary metabolite clusters variance could be interpreted by the size of the genome (Figure 11F).

Correlation between the number of secondary metabolite gene clusters uncovered using antiSMASH and the genome size was clearly established. Up to 20.3% of the variance in the number of SM clusters could be explained by genome size (Figure 11B). On the other hand, only 6.3% of the variance in the number of SM clusters could be interpreted by difference in genome size when using the PRISM software (Figure 11D).

### 2.13. Predicted SM Clusters Location within Pantoea Genomes

SM prediction in the core and the accessory genomes of the *P. agglomerans* and *P. vagans* collection showed numerous unknown SM (Figure 11E and Supplementary Table S5). Only carotenoid was observed in some strains of *P. agglomerans* and *P. vagans* (Figure 11E). A new representative of the aryl polyene family was also found in some strains (Supplementary Table S6). No secondary metabolites were located in the core genome of the *P. agglomerans* and *P. vagans* collection.

### 2.14. Characterization of the Core and the Pan Genome of P. agglomerans Genomes

**Figure 12:**
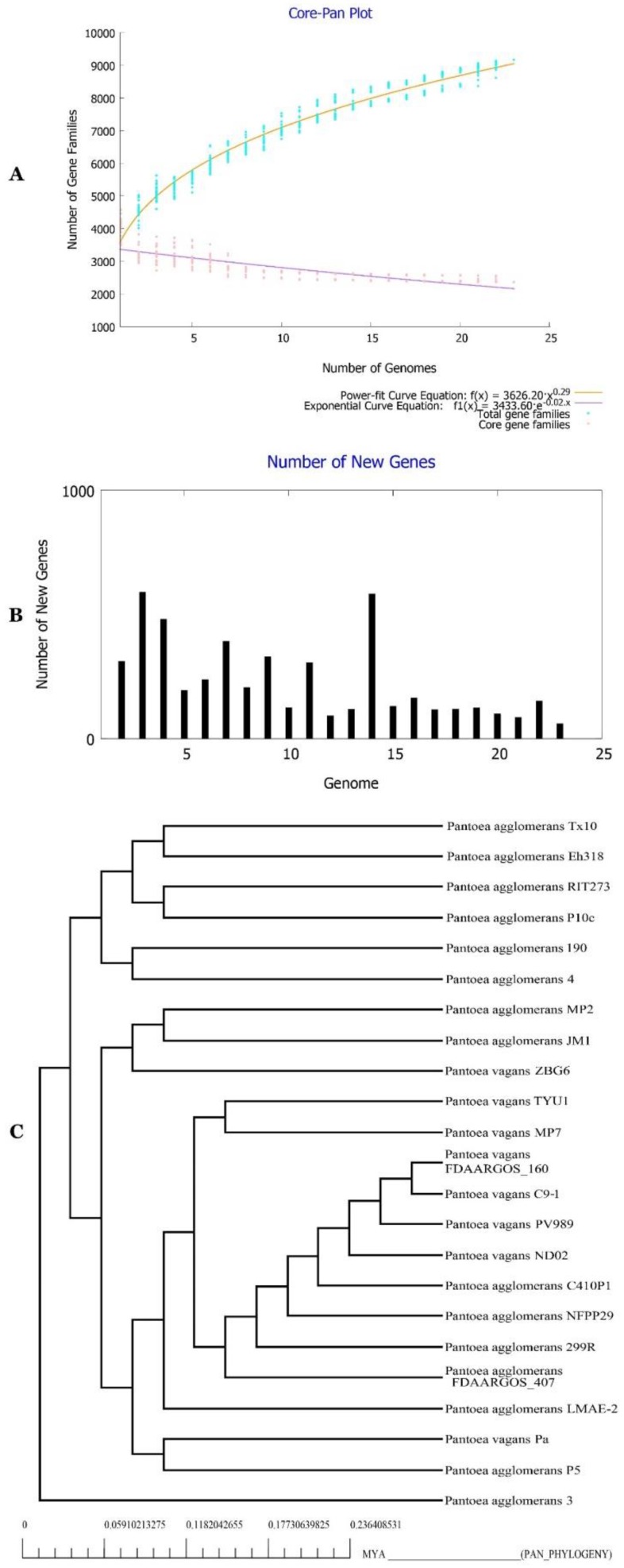
(**A**) Core–pan genome plot of *P. agglomerans* and *P. vagans* strains based on number of gene families and genomes. (**B**) New genes discovered in bacterial genomes. (**C**) Tree of Pan-genome of the bacterial strains.

Core and pan genome analysis of all bacterial isolates were achieved to explore genomic and metabolic capacities of the species, as suggested by Belbahri et al. [22]. This study was conducted using all genomes of the *P. agglomerans* and *P. vagans* collection described in Table 1. Figure 12A,B unambiguously documents an increase in the pan-genome of *P. agglomerans* after the rise in the number of genomes analyzed. Tettelin et al. [44] Heaps’ law allowed the conclusion that the *P. agglomerans* pan genome might be an open genome, given that it increased with a value of 0.29. Genome-based phylogenetic relationships among the *P. agglomerans* isolates collection recovered using the core-genome concatenated amino acid sequences revealed a phylogenetic tree that confirmed the GGD and ANI analysis results (Figure 12C).

### 2.15. Functional Characterization of the Core, Accessory, and Unique Genomes of P. agglomerans–P. vagans Strains Collection

**Figure 13:**
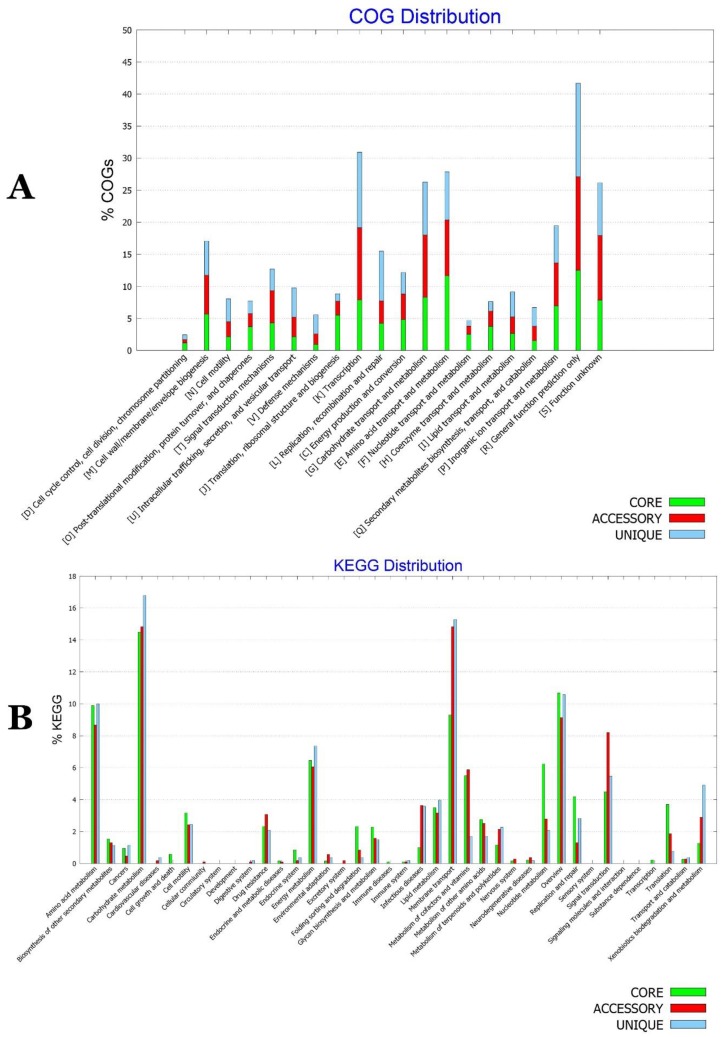
(**A**) COG and (**B**) KEGG distribution for accessory, core and unique genomes of *P. agglomerans*–*P. vagans* strains collection.

In Figure 13A, clear differences were visible between the core, accessory, and unique genomes of the species. Functional genes related to secretion and vesicular transport (U), defence mechanisms and intracellular trafficking (V) were enriched in the unique genome. Secondary metabolite biosynthesis and catabolism (Q) and signal transduction mechanisms (T) were enriched in the accessory genomes. However, energy production and conversion (C) and amino acid transport and metabolism (E) were enriched in the core genome. KEGG pathway investigation of metabolic features of *P. agglomerans–P. vagans* strains collection (Figure 13B) documented that signal transduction, drug resistance, and metabolism of terpenoids and polyketides were mainly present in the accessory genome. The unique genome was, however, enriched with the metabolism of terpenoids and polyketides, as well as xenobiotics biodegradation and metabolism along with amino acids, carbohydrates, and energy metabolism. Cell motility, nucleotide metabolism, and translation were fortified in the core genome of *P. agglomerans–P. vagans* strains collection.

### 2.16. GC-MS Analysis of P. agglomerans Strain Pa

GC-MS analysis of volatile organic compounds (VOCs) from *P. agglomerans* strain Pa revealed the presence of indole-3-acetic acid methyl ester and indole-3-acetamide, which are derivatives of the plant growth regulator auxin. Additionally, fatty acids such as palmitic, linoleic, and oleic acids have also been discovered in the volatile fraction of the bacteria (Supplementary Table S4). Indole-3-acetic acid methyl ester and palmitic acid were acknowledged for their capacity to alleviate salt stress in plants.

### 2.17. Metabolomics Profiling of P. agglomerans Strain Pa Under OSMAC Conditions

Small-scale fermentation of *P. agglomerans* strain Pa was performed using six different media as explained in Section 4.13. LC-HRESIMS raw data files of each medium inoculated with strain Pa culture and medium blanks were processed with MZmine 2.37 to produce a CSV file with possible formulae for peaks detected with their *m/z* and retention times. After a thorough clean-up process as explained in Section 4.13, the data set was dereplicated where 95 hits—including 29 possible new hits with diverse structural features—were identified under the selected six different media (M1 to M6) (Supplementary Table S5). The total of 95 hits can be classified tentatively as an array of quinolines, cyclic dipeptides, and thiazoles with reported therapeutic and plant growth-promoting activities.

**Figure 14:**
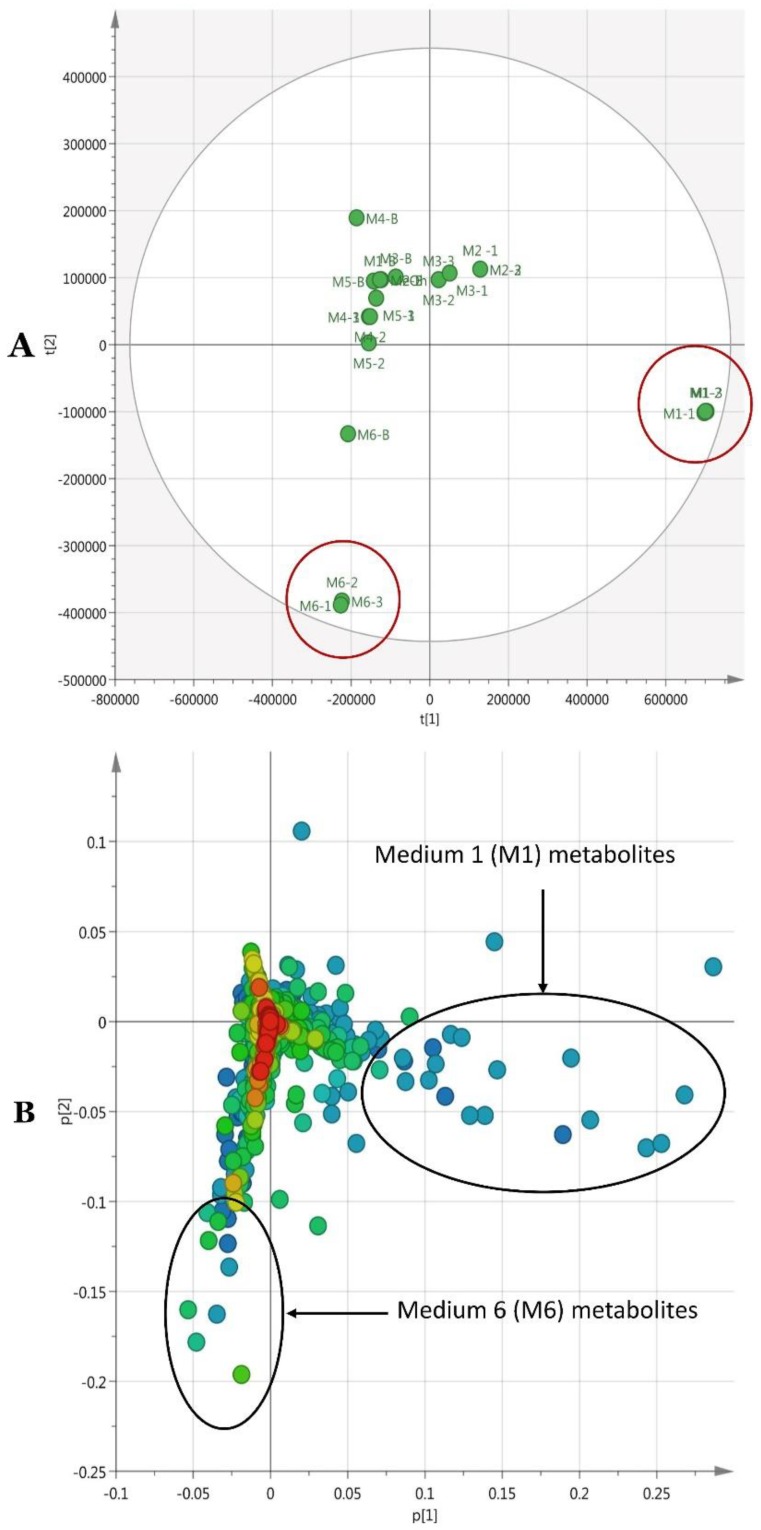
(**A**) PCA (R2 = 0.86, Q2 = 0.56) score plot of six different media (M1 to M6), media blanks (MB-1 to MB-6), and MeOH. (**B**) PCA loading plot of the six media (M1 to M6).

The final CSV file was further subjected to statistical analysis with SIMCA 14.1. The main intention of this statistical analysis was to figure out any significant differences between secondary metabolite profiles of the six different media, and select the best medium out of six for future large-scale fermentation. SIMCA 14.1 produced the score plot and loading plot with PCA method (Figure 14A,B), which is an unsupervised method. PCA analysis also helped to distinguish the distinct features associated with outlying media and, thereby, to optimize the best medium with interesting known and new secondary metabolites. This also helped to select the most effective medium in terms of richness of biologically-relevant secondary metabolites, and new and biologically un-assessed secondary metabolites. Isolation, structure characterization, and biological screening of predicted hits would be an interesting future research work.

When considering the PCA analysis of the six different media, M1 and M6 were laying furthest from the other four media, indicating different chemical profiles from the other four media. Moreover, the medium blank of M1 was positioned in a completely different quadrant in the score plot, confirming that the reported secondary metabolites are more likely produced only by strain Pa under the given M1 conditions. This was further clarified by overlaying raw LC-MS data sets on Xcalibur 4.1 (Supplementary Figure S5), which showed a well-defined LC-MS profile for M1 from its medium blank, while the other five media showed some similarities with their media blanks. Moreover, the PCA loading plot highlighted a higher number of outliers with M1, as marked within the Figure 14B, explaining a large number of distinct secondary metabolites with M1. This was further verified by identifying diverse secondary metabolites with some new hits during dereplication of M1 (Supplementary Table S5). Searching for biological activities of metabolites recovered from *P. agglomerans* strain Pa resulted in the description of numerous compounds with biological activity against phytopathogenic bacteria, fungi, and pests.

## 3. Discussion

Salinity and drought are drastic environmental factors limiting crop plants’ productivity [1,2]. The arid regions of traditional dryland farming, dependent on natural rainfall, are replaced mainly by irrigated farming to maximize crop yield [4]. Unfortunately, salinization caused by high levels of saltwater intrusion leads to adverse effects and challenges the sustainability of agriculture in these regions [5].

All these factors are reminiscent of the Bou-Saâda (South of Algeria) region where durum wheat *Triticum durum* L. cv Waha production is severely limited by abiotic stress, mainly drought and salinity. Habitat-adapted symbiosis is an ecological concept that endorses plant microbiome from these habitats to allow plants habitat-specific stress tolerance, and was proposed by Rodriguez et al. [7]. This concept was recently validated for numerous species such as rice [11], the halophyte *Limonium sinense* [14], and *Phragmites australis* [13] for their tolerance to high saline habitats. In order to improve wheat production in Bou-Saâda, we have screened wheat root epiphytic microbiome for high resistance to drought and salinity. Bacteria inhabiting soil strongly adhering to roots were isolated. The salt-tolerant PGPR were selected by culturing bacteria on media with different salt concentrations and checking for their PGP activities. The procedure resulted in the recovery of the *P. agglomerans* strain Pa, which had an optimal growth temperature of 30 °C. However, the strain could tolerate temperatures ranging from 10 to 40 °C. Optimal growth of *P. agglomerans* strain Pa occurred between pH 7 and 9. Pa strain tolerated 30% PEG concentrations with no statistical growth difference. Taken together, these data attest that Pa strain demonstrated high optimal growth temperatures, steady growth over a range of pH, and high osmotic tolerance, all reminiscent of the climate and soil characteristics of the Bou-Saâda region.

Using in vitro tests, strain Pa was able to efficiently solubilize phosphate and produce both siderophore and the phytohormone IAA. Strain Pa proved effective in producing extracellular enzymes, including cellulase, chitinase and protease, ACC deaminase, NH_3_, and cyanhydric acid. All these features confirm the PGP capabilities of strain Pa [20,21].

NaCl concentrations up to 1 M moderately interfered with the growth of strain Pa. Strain Pa at these salt concentrations was still able to solubilize phosphate, produce siderophores, and to a lesser extent produce IAA. These results attest that strain Pa is highly adapted to high salt concentrations in the Bou-Saâda region. These results agree with that of Qin et al. [12] for *Limonium sinense* (Girard) Kuntze bacterial microbiome.

All these PGP and bio-control features encouraged us to apply *P. agglomerans* strain Pa to wheat plants grown under salt stress conditions. While strain Pa had no clear influence on shoots and fresh weight of durum wheat plants grown in sand pots and subjected to salt stress, it showed an important effect on the dry weight of shoots and roots of durum wheat grown under salt stress conditions. Such findings suggest that the effect of PGPR should be studied at the level of dry weight rather than fresh weight to draw representative conclusions. Treatment with strain Pa significantly induced higher amounts of chlorophyll a, b, and a + b in durum wheat plants, independent of the type of treatment (0, 100, and 200 mM salt concentrations). These observations could explain the growth parameter differences at the level of the shoot and root dry weights of the plants treated with salt. Durum wheat plant treatment with either 100 or 200 mM salt concentration induced significant accumulation of proline, which was suppressed by Pa inoculation. Proline accumulation is known to be a typical response to salt stress in many plants [45]. It is believed that plants accumulate high proline levels when subjected to salt stress to sustain cell turgor and chlorophyll level, allowing efficient photosynthetic activity protection [46]. These findings are similar to those reported by Silini et al. [47]. Treatment of durum wheat plants with either 100 or 200 mM salt concentration induced significant enhancement of Na^+^ and depletion of K^+^ contents in the roots and shoots of durum wheat plants. In fact, salinity caused a disequilibrium in the ion flux within plant cells [48]. Pa inoculation strongly reduced Na^+^ and K^+^ levels close to control values, which agree with previous literature [48,49].

The growth of wheat plants on pots filled with soil from Bou-Saâda wheat fields confirmed the results obtained using wheat plants grown in sand pots. Additionally, growing plants until the recovery of ears allowed us to show that plants treated with Pa had more ears than control plants, using either 0 or 100 mM salt concentrations. Nonetheless, Pa strain enhanced the number of ears in comparison to the control in all concentrations. The ear numbers were despaired in the control sample and decreased in Pa-treated plants. Strain Pa did not increase the number of thalli of wheat plants at any of the salt concentrations tested. Taken together, our results are in explicit agreement with the habitat-adapted symbiosis ecological concept. The PGPR *P. agglomerans* strain Pa isolated from wheat roots sampled in Bou-Saâda confers alleviation of salt stress in durum wheat plants and confers considerable growth in this hostile environment.

Similar results using diverse *Pantoea* species have been documented in numerous studies, mainly in those of Panwar et al. [50,51], Paredes-Paliz et al. [52,53], and Li et al. [54]. The genus *Pantoea* have also been acknowledged as main actors in the shift of seed endophytic community between salt-sensitive and salt-resistant rice cultivars [55].

Towards its efficient use as a biofertilizer in wheat fields, the lifestyle of Pa strain should be uncovered. Therefore, triphenyl tetrazolium chloride (TTC) experiments targeting Pa strain and wheat roots allowed us to conclude that Pa strain can efficiently attach to the root surface. Additionally, using endophyte culture experiments of control and Pa-treated wheat roots allowed us to ascertain that Pa strain can colonize plant root tissues, and, therefore, to verify its lifestyle as an endophyte. For its practical use in the field, it is also mandatory to provide an easy mean of Pa inoculation on seeds, and to provide evidence that the strain can survive on the seeds during their storage. Our experiments unambiguously document that Pa strain can be efficiently encapsulated on seeds using carboxy methyl cellulose, and can survive more than one year on the seeds. These experiments on wheat plants grown from Pa-encapsulated seeds for 45 days allowed to show that Pa strain was able to survive in sterilized soil and compete with other microorganisms in non-sterilized root rhizosphere. These results strongly recommend that Pa strain can be used efficiently as a biofertilizer in wheat fields.

Phylogenomic analysis of strain Pa and 24 other isolates of *P. vagans* and *P. agglomerans* highlighted that the genus *Pantoea* suffers taxonomic imprecision that blurs species delimitation, which could have a profound impact on their practical use as biofertilizers. GGDC, ANI, and phylogenomic analysis revealed the presence of four different species sensu [27] for GGDC analysis, [38] for ANI analysis, and by the presence on the phylogenomic trees of four sister branches that could be used to define these species. GGDC, ANI, and phylogenomic analysis are considered as the gold standard for species’ definition and description [22]. Genome mining of the 25 *Pantoea* strains was conducted through homology-based identification of genes that could provide benefits to plants.

These analyses revealed that the vast majority of *P. agglomerans* and *P. vagans* strains possessed the mined genes. Therefore, strain Pa had a remarkable arsenal of genes contributing to plant-beneficial functions genes. This finding suggests the possibility of using this strain as a biofertilizer. Additionally, SM clusters present in the genome of the 25 isolates of *P. agglomerans* and *P. vagans* were uncovered using prediction informatics for secondary metabolomes (PRISM) [40], antiSMASH 3.0 [39], NP.search [42], NapDos [41], and BAGEL3 specific for bacterial bacteriocins [43].

Except for carotenoid and aryl polyene biosynthetic gene clusters, none of the remaining matched known secondary metabolites, suggesting they could be new metabolites. These results are in line with those reported by Belbahri et al. [22] for the species *Bacillus amyloliquefaciens*. Rarefaction analysis of SM clusters resulting from ongoing genome sequencing efforts highlighted that complete coverage is far from being attained, similar to what was reported for *Bacillus amyloliquefaciens* [22]. Using antiSMASH, a clear correlation was found between the size of the accessory genome and the number of gene clusters known to be involved in SM biosynthesis. None of the SM analyzed were located in the core genome of the *P. agglomerans* and *P. vagans* strains collection. They are located within the different strains’ accessory genome. Core accessory and unique genome analysis suggests that while basic housekeeping genes are allocated to core genome, secondary metabolites, xenobiotic degradation, and drug resistance are located in the accessory and unique genomes of the strains, as suggested earlier by Belbahri et al. [22]. We speculate that this result indicates genomic plasticity that allowed the strain to adapt and colonize its environment and adopt its lifestyle. These results are similar to what was observed for *Bacillus amyloliquefaciens* by Belbahri et al. [22].

LC-HRMS identification of the secreted metabolites under six different media revealed a diverse secondary metabolite arsenal with a total of 95 tentatively-identified hits. Among the findings from metabolomics study, secondary metabolites—which may be beneficial for plant growth such as pyochelin as an essential molecule with an ability to transport biologically relevant metals, quinoline derivative pseudane V and pyrrolam D with reported herbicidal activity, 4’-methoxycyclopeptin as an insecticide—were identified. Also, molecules which were reported to trigger cell–cell communication, a vital need for an efficient symbiotic association such as indole derivatives, dicyclic peptides such as cyclo(Pro-Phe), were also identified. Numerous other compounds such as oncorhyncolide used as antibiotic [56], cyclo(Gly-l-Phe), and cyclo(l-Tyr-l-Pro) were reported as antimicrobial with a biofilm-inhibiting effect [57,58,59]. These hits explain the PGP capabilities of strain Pa, making the experimental evidences discussed in this paper stronger and more logical. Moreover, during metabolomic profiling, it was revealed that strain Pa had a great ability to produce quinoline derivatives which were reported with good antiparasitic, anticancer, and antibacterial activities. Also, most of the thiazole compounds reported during metabolomics analysis were not tested before for their biological importance, and this will be a good avenue to test their possible novel bioactivities by large-scale bacterial fermentation and the isolation of these metabolites. It was interesting to utilize the OSMAC strategy to demonstrate a comprehensive statistical analysis, with the aid of modern metabolomics analysis and PCA as a tool for data visualization and interpretation to compare the influence of media variation on strain Pa to produce diverse secondary metabolites. Both dereplication and statistical analysis revealed that medium 1 (M1) had the highest ability to deliver a large set of SM with 52 hits, including 17 new hits. Therefore, performing large-scale fermentation using medium 1 is suggested to demonstrate a comprehensive chemical profiling including isolation, structure characterization, and biological screening. GC-MS analysis of the volatile fraction of M1 of strain Pa revealed the presence of IAA methyl ester and indole-3-acetamide [60,61]. Therefore, these results confirm in vitro detection of IAA. Additionally, palmitic, linoleic, and oleic acids that enhance seedlings growth and provide efficient protection against phytopathogenic fungi were also detected [60].

Taken together, our results strongly attest that *P. agglomerans* strain Pa, isolated in the time course of this study, could be used efficiently to promote wheat growth in arid and salty areas. Its implementation in seeds after encapsulation is an excellent mean of delivery of the strain to the wheat seeds. Ongoing results are targeting rhizosphere characterization of Pa-inoculated plants, as well as the potential of Pa strain to be used for other plants such as legume culture and trees.

## 4. Materials and Methods

### 4.1. Sampling Location and Soil Physicochemical Characterization

Rhizosphere samples of durum wheat used for the isolation of rhizobacteria were collected from durum wheat fields in the arid Bou-Saâda region located in the south of Algeria (35°23′38″ N, 4°19′18.1″ E). The rhizosphere samples were kept at 4 °C during transfer to the laboratory of applied microbiology (University Ferhat Abbas, Setif, Algeria) and used immediately. The soil of durum wheat fields was characterized by measuring pH, electro-conductivity (EC), calcium carbonate, carbon, and organic matter content. Briefly, soil pH and conductivity were determined using a pH meter (Hanna HI2209, Setif, Algeria) and a conductivity meter (Hanna HI9032, Setif, Algeria). Loss of ignition of the samples after 5 h at 550 °C allowed estimation of organic matter content [62]. Soil physicochemical characteristics are presented in Supplementary Table S1.

### 4.2. Bacterial Isolation from Rhizosphere of Healthy Durum Wheat

One gram of soil was incubated in 200 mL Erlenmeyer flasks containing 10 mL of sterile distilled water (Thermo Fisher Scientific, Carlsbad, CA, USA) and shaken vigorously (200 rpm, 30 min). Bacterial isolation was performed by plating parts of the diluted rhizosphere soil extract on Winogradsky Salt (WS) medium. Plates were incubated (30 °C, 48 h) to yield around two hundred isolates where pure cultures were established by re-culturing a few times on nutrient agar with single bacterial colonies. PGP activities were tested for all strains, and 22 strains were selected based on their PGP abilities (Supplementary Table S2). PGPR with high tolerance to salt were selected by culturing the bacteria on different salt concentrations and checking for their PGP traits under salt stress according to Cherif-Silini et al. [62]. Strain Pa was selected as the best bacterial isolate with high PGP activities in the initial testing, the highest resistance to salt stress and the highest PGP activities under salt stress, and used in downstream experiments.

### 4.3. Effect of Temperature, PEG, and pH

To examine the ability of strain Pa to tolerate different temperatures, nutrient broth liquid media (Sigma-Aldrich, Sigma-Aldrich, Buchs, Switzerland) were seeded with 100 μL Pa culture and incubated at indicated temperatures for 2 days. Bacterial growth was then estimated by 600 nm spectrophotometric estimation of optical density (Hanna HI83200, Setif, Algeria). All measurements were performed in triplicate. For PEG effect, nutrient broth liquid media (Sigma-Aldrich, Buchs, Switzerland) containing 0, 10, 20, 30, and 40% PEG were inoculated with 100 μL of strain Pa culture and incubated at 30 °C for 2 days. Bacterial growth was then assessed by measuring optical density at 600 nm. All measurements were carried out three times. A Similar experiment was conducted for pH effect on bacterial performance. Nutrient broth liquid cultures with indicated pH values prepared in triplicate were inoculated with 100 μL strain Pa culture. The growth of strain Pa was then estimated by 600 nm optical density measurement.

### 4.4. Measurement of PGP Activities

#### 4.4.1. Direct PGPR Activities

Direct PGP activities were carried out three times.

##### Growth on Nitrogen-Free Medium

The WS medium [63] was used to check ability of strain Pa to grow on nitrogen-free medium.

##### Phosphate Solubilization

This assay was conducted as described by Gaur [64]. Pikovskaya (PVK) medium (Sigma-Aldrich, Buchs, Switzerland) containing tricalcium phosphate was used in this assay. Ten μL of strain Pa culture was then spotted on the PVK agar surface and incubated at 30 °C for 7 days. The appearance of a halo around the colony indicates phosphate solubilization. Halo diameter size reflects the phosphate-solubilizing ability.

Tricalcium phosphate solubilization assay in liquid medium for quantitative analysis of phosphate solubilization was conducted according to Olsen et al. [65]. Briefly, absorbance was measured at 610 nm and compared to a standard calibration curve using KH_2_PO_4_ solution.

##### Siderophores Production

Semi-quantitative production of siderophore was screened according to [66] using Chrome Azurol S (CAS) media (Sigma-Aldrich, Buchs, Switzerland). Siderophore production (SP) was evaluated using the formula described in [64] and expressed in percent:

SP (%) = OD of the sample/OD of the control (CAS solution)

##### Indole Acetic Acid (IAA) Production

Winogradsky medium containing 2 g·L^−1^ of tryptophan was seeded (100 μL of strain Pa culture) and used to test the production of IAA according to Slama et al. [17]. IAA concentration was estimated by the use of a standard calibration curve generated by applying IAA solutions (0 to 10^−3^ M).

#### 4.4.2. Indirect PGP Activities

Indirect PGP activities examined in the current study have been performed in triplicate.

##### Protease Production

The development of clear zone tested protease activity after inoculation of strain Pa on skimmed milk agar media (Sigma-Aldrich, Buchs, Switzerland) [67].

##### Chitinase Production

The chitinase activity of the bacterial strain Pa was checked by culturing on chitin agar (Sigma-Aldrich, Buchs, Switzerland) plates [17]. The plates were then incubated and chitinase activity revealed by the presence of a clear zone around colonies.

##### ACC Deaminase Production

The ability of Pa strain to produce ACC deaminase was confirmed by their growth on minimal media containing ACC as sole nitrogen source according to [68].

##### NH_3_ Production

NH_3_ production was assessed according to Slama et al. [17]. Briefly, peptone water was seeded with strain Pa culture and kept at 30 °C for 48 h. Yellowish to brownish color development upon the addition of Nessler’s reagent (0.5 mL, Sigma-Aldrich, Buchs, Switzerland) indicated the production of NH_3_.

##### Hydrogen Cyanide (HCN) Production

HCN production by strain Pa was estimated according to Slama et al. [17] on HCN medium (nutrient agar supplemented with 4.4 g·L^−1^ of glycine), and development on the Whatman paper of an orange to red color (Thomas Scientific, CA, USA) indicated the production of HCN.

### 4.5. Bacterial DNA Extraction and Amplification

DNA extraction, PCR amplification, and molecular identification of bacterial isolates was performed according to Mlaik et al. [69]. Primers, fD1 (5′ AGAGTTTGATCCTGGCTCAG 3′) and rP2 (5′ ACGGCTACCTTGTTACGACTT 3′) [70] were used in the study.

#### DNA Sequencing and Phylogenetic Analysis

The purified amplicons were sequenced in both directions using an ABI 3130 XL available at the iGE3 (Institute of Genetics and Genomics in Geneva, University of Geneva Medical Center (CMU), Switzerland). The exact phylogenetic position has been ascertained by phylogenetic analysis using the multiple sequence alignment web-based MAFFT program [71], the maximum-likelihood (ML) algorithm [72], MEGA software v.6 [73], and the Kimura 2-parameter model [74]. The validity of branches in the trees was evaluated by bootstrap analysis. (Supplementary Figure S2).

### 4.6. Bacterial Genome Sequencing Assembly and Annotation

The genome of strain Pa was sequenced using the facilities available in the iGE3 genomics platform of the University of Geneva (http://www.ige3.unige.ch/genomics-platform.php). De novo assembly resulted in 45 contigs. *P. agglomerans* strain Pa genome sequence has been deposited in the genome sequence database (accession no. MUJJ00000000.1).

#### 4.6.1. Selection of Genomes for Phylogenomic Analysis

Genomes of *P. agglomerans* and *P. vagans* isolates targeted in this study were selected among those submitted to GenBank genome sequence database (Table 1).

#### 4.6.2. Whole Genome Phylogeny

The reference sequence alignment-based phylogeny builder, available at http://realphy.unibas.ch, was used to generate genome alignments [75].

#### 4.6.3. Average Nucleotide Identity Analysis

Average nucleotide identity (ANI) values of *P. vagans* and *P. agglomerans* strains were estimated using the server EzBioCloud, available at http://www.ezbiocloud.net/tools/ani [38,76,77].

#### 4.6.4. Genome-to-Genome Distance Calculator (GGDC) Analysis

DSMZ software, available at http://ggdc.dsmz.de [27], was used to assess in silico genome-to-genome distance values.

### 4.7. P. agglomerans Pa Strain Location within Wheat Plant Root Tissues

*P. agglomerans* Pa strain ability to attach to plant root surface was studied visually by microscopic analysis using the triphenyl tetrazolium chloride (TTC) procedure [78]. Wheat roots were incubated in a *P. agglomerans* strain Pa 10^8^ bacterial solution for 24 h, followed by extensive washes using sterile distilled water and incubation in a TTC solution (0.15% TTC in PBS 0.06 M, pH 6.8) for 2 h. Microscopic observation was then conducted to detect the presence of bacteria on the surface of the root. The development of rose areas indicates the presence of huge amounts of bacteria reducing colorless TTC. This procedure provides a qualitative estimation of *P. agglomerans* Pa strain bacterial attachment to root surface.

To test *P. agglomerans* strain Pa potential to colonize plant root tissues, and therefore to ascertain its life style, roots were collected from 45-day-old wheat plants grown from CMC-encapsulated wheat seeds (Material and Methods 4.8) grown on sterilized compost substrate (Material and Methods 4.8). Surface sterilized roots (1 g) (Material and Methods 4.8) were then ground in 10 mL sterile 9 g/L NaCl solution. A serial dilution up to 10^-6^ was performed and spread on the surface of LB agar petri dishes. Identification of *P. agglomerans* strain Pa was performed by microscopic observation, amoxicillin resistance, and subsequent biochemical identification using Galerie API 20E (Biomerieux, France). Bacterial counting was performed after 45 days. Results were expressed as CFU/g of root fresh weight.

### 4.8. P. agglomerans Strain Pa Survival on Wheat Seeds

Surface sterilized wheat seeds were obtained by successive incubation in ethanol (70%, 1 min) and sodium hypochlorite (2%, 30 min), followed by several washes using sterilized distilled water. *P. agglomerans* Pa strain cultures were centrifuged (7000 rpm, 15 min) and the pellet resuspended in sterile 1% carboxy methyl cellulose (CMC) at 10^8^ bacteria/mL [79]. The mixture was then shaken for 3 h, air-dried overnight in a laminar hood, and conserved at 4 °C in the obscurity. Seeds mixed with CMC without bacteria were considered as a negative control for the rest of the experiment. Bacterial treated seeds (1 g) were resuspended in 10 mL sterile 9 g/L NaCl solution and vortexed for 5 min. A serial dilution up to 10^−6^ was performed and spread on the surface of LB agar petri dishes. Bacterial counting was performed after 48 h, 45 days, 7 months, and one year. Results were expressed as CFU/treated seed.

### 4.9. P. agglomerans Strain Pa Survival on Sterilized and Non-Sterilized Wheat Rhizosphere

For testing *P. agglomerans* strain Pa survival on sterilized wheat rhizosphere, the compost used as substrate for wheat culture was successively sterilized three times at 30 °C for 24 h. Then, an equal quantity of sterile compost was distributed in disinfected pots by sodium hypochlorite. Sterile and *P. agglomerans* strain Pa CMC-encapsulated wheat seeds prepared as described in Material and Methods Section 4.8 were sown 1 seed/pot in sterile conditions. Each set was composed of three pots and a total of six sets was performed. Pots were then incubated (25 °C, 16 h light photoperiod) in culture room. The experiment was conducted for 45 days. To test the viability of *P. agglomerans* strain Pa in soil, 1 g of compost strongly adhering to wheat roots was incubated in 10 mL sterile NaCl solution (9 g/L) and vortexed for 5 min. A serial dilution up to 10^-6^ was performed and spread on the surface of LB agar petri dishes. Bacterial counting was performed after 45 days. Values were presented as CFU/g root fresh weight.

For testing *P. agglomerans* strain Pa survival on non-sterilized wheat rhizosphere, the same procedure was conducted for testing bacterial survival in sterilized compost, except that compost used as substrate was not sterilized and bacterial survival tested after 2, 8, 15, 22, and 30 days.

### 4.10. Plant Inoculation and NaCl Treatment

#### 4.10.1. Durum Wheat Seeds Sterilization and Bacterial Inoculation

Durum wheat (*Triticum durum* L. c.v. Waha) seeds obtained from the Setif Technical Institute of Field Crops (Algeria) were surface sterilized. Strain Pa was grown for 2 days in liquid medium at 30 °C, centrifuged at 12,000 rpm for 10 min, washed twice in phosphate saline buffer (0.9%, pH 7.2), and density was adjusted to 10^8^ CFU/mL. Seeds inoculation was conducted by immersing seeds for 1 h in the bacterial suspension. Control seeds were treated with sterile water and then germinated at 20 °C for 48 h in dark.

#### 4.10.2. Durum Wheat Growth in Pots Filled with Sand

Plastic pots (10 cm in diameter) were filled with 200 g of sterile sand. ½ Hoagland’s Complete Nutrient Solution [62] were added in each pot and used in subsequent experiments. Three sets of pots were treated with 0, 100, and 200 mM NaCl, respectively.

Treated seeds were sowed (one seed per pot at 1 cm depth from the surface). Six seeds represented each treatment. Growth was followed during 45 days in the growth chamber. Light intensity was set at 2100 Lux, while humidity was adjusted and maintained throughout the experiment while irrigating with distilled water.

#### 4.10.3. Durum Wheat Growth in Pots Filled with Soil of Bou-Saâda Durum Wheat Fields

Wheat seeds were soaked for 1 h in water (control) and in Pa bacterial cell suspension (assays). Seeds were then sown at 1 cm depth in 20 cm diameter pots filled with soil (1 Kg). Seed density was at 10 seeds per pot. Soil physicochemical parameters were: pH 7.99, E_C_ 0.83 mS/cm, CaCO_3_ 8%, Carbon 1.95%, and organic matter 3.35%. After seed germination, seedling numbers were reduced to 8 seedlings per pot. Three groups of pots were treated with 0, 100, and 200 mM salt, respectively. The pots were irrigated twice a week with tap water for the control (400 mL approximately) and the same volume of 100 and 200 mM NaCl solutions for 100 and 200 mM groups, respectively. The treatments with 100 and 200 mM NaCl solutions were maintained until obtaining a soil corresponding to the 100 and 200 mM NaCl solutions, respectively. The experiments were repeated three times. At the two leaves stage, a second bacterial inoculation was performed through irrigation using 1 mL of bacterial cell suspension prepared as described above. The pots were organized using a random design under 16–26 °C temperature, 16/8 h photoperiod. After 10 weeks (ear stage), all parameters were recorded, and sampling for biochemical analysis was performed.

#### 4.10.4. Growth Parameters Analysis of Control and Bacterial Inoculated Durum Wheat

At the 4–5 leaves stage, plants were washed thoroughly with distilled water. Roots and shoots were then separated, and their length, and fresh and dry (after incubation at 65 °C for 72 h) weights were measured. For plants grown in soil pots, thallus and ear numbers were also counted.

#### 4.10.5. Biochemical Parameters of Control and Bacterial Inoculated Durum Wheat

All biochemical analyses (chlorophyll content, Na+ and K+, and proline contents) were performed in triplicate.

Chlorophyll (Chl) a and b contents were determined according to Cherif-Silini et al. [62]. Formulae used to estimate chlorophyll a and b content are detailed below:

Chl_a_ (mg/L) = 12.41 OD_663_ − 2.59 OD_645_

Chl_b_ (mg/L) = 22.9 OD_645_ − 4.68 OD_663_

Chl_a + b_ = Chl_a_ + Chl_b_

Na^+^ and K^+^ content of shoots and roots were determined after mineralization by flame atomic absorption spectrometry (Hanna HI83200, Setif, Algeria) following the recommendations of Cherif-Silini et al. [62].

Proline extraction was conducted according to Naidu et al. [80] and OD was measured at 520 nm [81].

### 4.11. Secondary Metabolites Profiling and Dereplication Using Metabolomics Analysis

#### 4.11.1. Small Scale Fermentation Under OSMAC Conditions and Metabolites Extraction

Glycerol stock of strain Pa was cultured on ISP2 agar plate under sterile conditions and incubated for 48 h at 27 °C. Six different media (M1: Malt extract 4 g/L, yeast 4 g/L, glucose 4 g/L, lab lemco 2 g/L. M2: Yeast 1 g/L, MOPS 0.2 g/L, glycerol 10 g/L, bacto-casitone 2 g/L, yeast autolysate 1 g/L, Lab lemco 1 g/L, CaCO_3_ 1 g/L. M3: Malt extract 4 g/L, yeast extract 10 g/L, glucose 10 g/L. M4: Yeast extract 1 g/L, bacto-casitone 2 g/L, lab lemco 0.8 g/L. M5: Sucrose 50 g/L, Casamino acid 0.2 g/L, yeast extract 4 g/L, MOPS 5 g/L. M6: Mannitol 4 g/L, NZ-amine 2 g/L, MgSO_4_ 0.2 g/L, CaCO_3_ 2 g/L, K_2_HPO_4_ 0.2 g/L, NaCl 2 g/L) were prepared in 1 L fermentation flasks (300 mL) in quadruplicate and autoclaved. Three flasks of each medium were inoculated with a single colony from freshly-prepared for 48-h-old strain Pa culture plate under a sterile condition, and the fourth flask of each medium was used as the medium blank. All 24 flasks were fermented on a shaker at 180 rpm for 7 days at 30 °C. At the end of fermentation process (day 7), about 50 g/L Diaion HP20 resins (Sigma-Aldrich, Buchs, Switzerland) was added into the fermentation flask (Thermo Fisher Scientific, CA, USA), left shaking for 6 h, then each flask was filtered under vacuum to separate the HP20 resins—which were then extracted separately with methanol to produce 24 total extracts of six different media after evaporation of methanol under vacuum.

#### 4.11.2. Preparation of Samples for LC-HRMS Analysis

For LC-HRMS analysis, 1 mg of the methanolic extract was dissolved in 10 mL methanol filtered through 0.2 µm PTFE filter (Milian, Geneva, Switzerland) and placed into HPLC vial prior to LC-HRMS analysis.

#### 4.11.3. LC-MS Analysis: HPLC-HRMS Instrumentation and Conditions

HRESIMS data were obtained using a Thermo LTQ Orbitrap coupled to an HPLC system (PDA detector, PDA auto-sampler and pump). All conditions were according to Slama et al. [17].

#### 4.11.4. Raw Mass Data Analysis Using MZmine 2.3.7

Raw mass data files were sliced into two data sets as positive and negative based on ionization mode using MassConvert tool from Proteo Wizard. As the negative mode was not high resolution, only positive mode files were considered for further process. Positive mode sliced data files of all samples and sample blanks belonged to six different media were imported and processed using MZmine 2.37, following the steps and predefined parameters explained in [82]. The sequence of steps involved peak detection (mass detection and chromatogram builder), chromatogram deconvolution, deisotoping, filtering, alignment, gap filling, adduct and complex ion search, and finally formula prediction. Final data output from the MZmine was exported as a CSV file and further clean-up was done manually by removing adducts and peaks present in both samples and their medium blanks as explained in [82]. Additionally, the formulae which did not follow the nitrogen rule were also eliminated. The CSV file was cross-checked with Xcalibur 3.1 using raw mass data files to eliminate unreliable formulae, and the unidentified peaks by MZmine were also evaluated for reliable formulae and the intensity before elimination.

#### 4.11.5. Dereplication Process to Identify Known and New Hits

The CSV file, after the clean-up process, was aligned in ascending order with the peak retention times. Predicted formulae were searched using databases such as Antibase 2012, SciFinder, Dictionary of Natural product 27.0, DEREP-NP 2013, and REAXYS online databases. This was followed by a thorough search of the fragmentation patterns of the precursor ions on Xcalibur 3.1 with the aid of a fragmentation tool available with ChemDraw professional 17.0 (PerkinElmer Informatics, Cambridge, UK) to make lists of best possible hits from each medium. Finally, all six lists were combined to produce a comparison table with tentative identification, chemical structure, and reported biological importance of the predicted hits (Supplementary Table S5).

#### 4.11.6. Multivariate Analysis for Media Optimization

The CSV file, after an extensive clean-up process as explained in the Section 4.16, was uploaded into SIMCA 14.1 (Umetrics, Umeå, Sweden) for statistical analysis to select the best medium out of six for future large-scale chemical profiling. During the analysis process with SIMCA, the feature ID number was set as the primary ID, while the retention time, m/z of molecular ion peak, the peak area, and possible molecular formulae were set as the secondary IDs. Then, the data set uploaded into SIMCA was analyzed using unsupervised statistical analysis method, PCA (principal component analysis) score plots and loading plots were generated to compare the variations between samples, as well as the secondary metabolite profiles, respectively.

#### 4.11.7. GC-MS Analysis

Analysis was performed using procedures described in Slama et al. [17].

### 4.12. Homology-Based Identification of Genes Contributing to Plant-Beneficial Functions

Comparative genomics analysis was performed according to detailed procedures given in supplementary materials and methods.

### 4.13. SM Clusters Identification

NapDos [41], antiSMASH 3.0 [39], NP.search [42], prediction informatics for secondary metabolomes (PRISM) [40], and BAGEL3 [43] were used in the course of this study to analyze and predict secondary metabolite gene clusters in annotated draft genome sequence files (Supplementary Table S1).

### 4.14. Identification of Core Genome and Accessory Genomes of the Strain Collection

The core genome, commonly designed as the subset of the genome present in all the strains analyzed for a given bacterial species, was determined using the Spine [83]. Agent was, on the other hand, used to determine the accessory genome of each isolate of the *Pantoea* collection [83].

### 4.15. Comparative Genomics Analysis of P. agglomerans Strains

Pan and core genomes of *P. agglomerans* and *P. vagans* strains collection (Supplementary Table S1) were performed using the BPGA pipeline [84]. Functional annotation based on KEGG Orthology (KO) were done using BlastKOALA analysis [85].

### 4.16. Statistical Analysis

The experiments were repeated three times and results were expressed as mean ± standard error of the mean. The data were analyzed using SPSS 22 and GraphPad Prism 8. One-way ANOVA and two-way ANOVA were used to analyze the data to find whether there was a significant effect of the treatment compared to the control sample in the presence of three different salt concentrations—0, 100, and 200 mM. A significant level at 5% (*p* < 0.05) was used, and Tukey’s multiple comparison tests were performed when a significant difference was encountered. In addition, multivariate data analysis was performed with SIMCA 14.1 software.

## Figures and Tables

**Table 1:** List and characteristics of *Pantoea* strains used in the current study.

Species		Strain	Genome Size (Mb)	Plasmid	Description/References	GenBank Accession
*Pantoea agglomerans*	1	C410P1 (Complete)	5.11524	Y	Isolated from lettuce in China (Anhui province); Biocontrol agent	CP016889.1
	2	FDAARGOS_160 (Complete)	4.80881	Y	Isolated from wound swab of human (*Homo sapiens*); Children’s National Hospital, the United States	CP014129.1
	3	Tx10 (Scaffold)	4.85699	N	Isolated from sputum from a cystic fibrosis patient (*Homo sapiens*) in Canada; produces multiple antibiotics that are effective against *Erwinia amylovora, Staphylococcus aureus*, *S. epidermidis*, and *Escherichia coli*; biocontrol agent [28]	ASJI00000000.1
	4	Eh318 (Scaffold)	5.03584	N	Isolated from the stem of an asymptomatic apple (NY, the United States); produces two antibiotics, named pantocin A and B, that are effective against *Erwinia amylovora* [29]	AXOF00000000.1
	5	190 (Scaffold)	5.00257	N	Isolated from soil in South Korea [30]	JNGC00000000.1
	6	MP2 (Scaffold)	4.73383	N	Isolated from fungus-growing termites (*Macrotermes natalensis*) in South Africa [31]	JPKQ00000000.1
	7	IG1 (Contig)	4.82958	N	Isolated from Japan; grows symbiotically with various plants; the lipopolysaccharides derived from *P. agglomerans* IG1 were effective in the prevention of various diseases, such as bacterial or viral infection, lifestyle-related diseases [32]	BAEF00000000.1
	8	NBRC 102470 (contig)	4.65204	N	Isolated from Japan	BCZA00000000.1
	9	DAPP-PG734 (Contig)	5.36593	N	A hypersensitive reaction-inducing *Pantoea agglomerans* strain isolated from olive knots caused by *Pseudomonas savastanoi* pv. *savastanoi* [33]	JNVA00000000.1
	10	4 (Scaffold)	4.82789	N	Isolated from wheat (*Triticum aestivum*) seed in Canada	JPOT00000000.2
	11	3 (Scaffold)	4.81358	N	Isolated from wheat (*Triticum aestivum*) seed in Canada	LVHW00000000.1
	12	824-1 (Scaffold)	4.98907	N	Elicits galls on *Gypsophila paniculata* and hypersensitive response (HR) on beet; isolated from NY (the United States)	LXSX00000000.1
	13	4188 (Scaffold)	5.002	N	Isolated from *Beta vulgaris* in NY (the United States); causes galls on beet and gypsophila	LXSW00000000.1
	14	P5 (Scaffold)	3.52764	N	Isolated from soil (desert) in Iran	NGNU00000000.1
	15	NFPP29(Scaffold)	4.10462	N	-	FUWI00000000.1
*Pantoea agglomerans*	16	LMAE-2 (Contig)	4.98116	N	Isolated from seabed sediment moderately contaminated with Cu^2+^ in Puerto Montt (Chile); a copper-resistant marine bacterium with potential use in bioremediation [34]	JWLQ00000000.1
	17	P10c (Contig)	4.77592	Y	Isolated from pear blossoms in New Zealand; ability to rapidly colonize apple and pear flowers and to suppress fire blight disease; developed as a commercial biocontrol agent [35]	LIME00000000.1
	18	DSM 3493 (contig)	4.65938	N	Type strain of *Pantoea agglomerans*	FYAZ00000000.1
	19	Pa (Contig)	4.79516	N	Isolated from rhizosphere of durum wheat in arid environment Bousaada (Algeria)	MUJJ00000000.1
*Pantoea vagans*	20	ZBG6 (Contig)	4.729	N	Isolated from soil in Zellenberg (France)	LFQL00000000.1
	21	PaVv11 (Scaffold)	4.85077	N	-	CEFP00000000.1
	22	C9-1 (Complete)	4.88834	Y	Commercially registered for biological control of fire blight, a disease of pear and apple trees caused by *Erwinia amylovora* [36]	CP002206.1
	23	MP7 (Scaffold)	4.5987	N	Isolated from fungus-growing termites (*Macrotermes natalensis*) in South Africa [31]	JPKP00000000.1
	24	RIT273 (Contig)	5.36534	N	Isolated from Willow (*Salix* sp.) in NY (the United States)	JFOK00000000.1
	25	299R (Contig)	4.58148	N	A spontaneous rifampin-resistant derivative of the phyllosphere model bacterium isolate 299, a pigmented bacterium that was recovered from healthy leaves of a Bartlett pear tree near Healdsburg, CA [37]	ANKX00000000.1
	26	848_PVAG (Contig)	4.99032	N	Isolated from human (*Homo sapiens*) in WA (the United States)	JUQR00000000.1
	27	LMG 24199 (Complete)	4.79033	2	Type strain of *Pantoea vagans*	CP038853.1

## Supplementary Materials

Supplementary materials can be found at https://www.mdpi.com/1422-0067/20/16/3989/s1.
