# Supplementary material for: Durum Wheat Stress Tolerance Induced by Endophyte Pantoea agglomerans with Genes Contributing to Plant Functions and Secondary Metabolite Arsenal
Source: Int J Mol Sci. 2019 Aug 16;20(16):3989. doi: 10.3390/ijms20163989 (PMC6720286; doi:10.3390/ijms20163989)
Supplement: Supplementary file 1 [file ijms-20-03989-s001.pdf]

Supplementary Materials and Methods:

Homology based mining of genes contributing to plant-beneficial functions

Nutrient acquisition

Nitrogen fixation

nifHDK operons encoding the structural proteins of nitrogenase in proteobacterial PGPR *Azospirillum* and *Burkholderia* and genes *nifS* and *nifU* involved in nitrogen fixation were used as bait to search for similar sequences in the genome of *Pantoea* spp. collection analyzed [1].

Mineral phosphate solubilization

The genome collection used in this study have been mined using the pyrroloquinoline quinone-encoding genes *pqqBCDEFG* in the PGPR *Pseudomonas fluorescens* F113, *Erwinia herbicola* and *Enterobacter intermedium* [2-4].

Phytase

Bacillus velezensis SQR9 3 phytase encoding gene was used as a query in homology search in the *Pantoea* spp. genomes for phytase putative encoding genes [5].

Urea utilization

Homologs of the genes *ureABC* of *Bacillus subtilis* strain 168 were searched using BLAST to recover urease genes in *Pantoea* spp. studied genomes [6].

Exoenzymes production

Two approaches have been used to identify and characterize genes encoding proteases, lipases, cellulases, pectinases, amylases, laccases, xylanases and lichenases exoenzymes; i) keyword search of the different enzymes followed by identification of secreted enzymes using SignalP 4.1 [7] or ii) using exoenzyme sequences described in closely related species in BLAST search of the different genomes analyzed in this study [6].

PGPR fitness conferring genes

Phenazine encoding genes (*phzADEFG*), aiding in long term survival and ability to compete with the resident microflora, were searched in the different studied genomes by a BLAST approach [8]. Other fitness conferring genes involved osmo protectant glycine betaine synthesis genes *gbsAB* genes [9], heat-shock protein genes *dnaJ, dnaK, groES, grpE, htpX, hspQ, hsp90, ibpA, yrfH, hslV, yegD, clpP* and cold shock protein genes *cspA, cspC, cspD, cspE* and *cspI* [10]. Type II secretory pathway have been mined using *hofC, hofB, ppdB, pilD, glgX, hofQ* [11]. Additionally, type III secretory pathways were identified using homology searches with *fliF, fliH, fliN* genes [12].

Root colonization and growth promotion factors

Swarming motility

Homologs to gene clusters *flgBCDEGKLMN*, *flhABCDE* and *swrABC* have been searched in the genomes of the different *Pantoea* spp. targeted in this study [12].

Chemotaxis ability

The different *Patoea* genomes have been checked for the presence of homologs of *cheARVWZ, fliADGHIJLMNPQRS* operons involved in the regulation of *B. subtilis* chemotactic response. Additionally, homologs of *Bacillus velezensis* UCMB5113 *motABPS* operon that control cell envelope and cellular processes motility and chemotaxis have been blast searched in the collection of genomes analyzed [6].

Rhizosphere colonization

The presence of the critical site recombinase *xerCD* gene for the PGPRs to be effective rhizosphere colonizers has been checked in the different analyzed *Pantoea* genomes [13].

Exopolysaccharide biosynthesis

Using *Bacillus subtilisepsA-O* operon genes, *tapA*, *tasA*, *sipW*, *pgsB* and *bslA* genes [14] as baits in homology-based searches allowed the recovery of genes encoding exopolysaccharide biosynthesis in *Pantoea* genomes.

Plant growth-promoting traits: hormones

Auxin biosynthesis

Different tryptophan dependent pathways for biosynthesis of the auxinic phytohormone indole acetic acid (IAA) known in *Bacillus* species have been mined i) indole-3-pyruvate (*ipyA*) pathway involving the tryptophan transaminase (*patB*), indole-3-pyruvate decarboxylase (*yclC* and *yclB*) and indole-3-acetaldehyde dehydrogenase (*dhaS*) genes, ii) indole-3-acetonitrile (IAN) involving the nitrilase gene (*yhcX*) and iii) uncharacterized IAA biosynthesis pathway involving tryptophan acetyltransferase gene (*ysnE*) [5, 15, 16]. Homologs of the gene *ywkB*, that may be involved in the transport of auxin out of the bacterial cell and its redistribution to the plant roots and encoding a putative auxin efflux carrier protein, have also been fetched in the different genomes [6].

Cytokinin biosynthesis

The *Pantoea* genomes targeted in this study have been mined for the presence of the *miaABE* gene encoding tRNA dimethyl allyltransferase that removes a zeatin precursor from tRNA and the *Agrobacterium tumefaciens* tzs (trans-zeatin synthase) gene [17].

Phenylacetic acid

The gene *ipdC* involved in the biosynthesis of phenylacetic acid (PAA) in *Azospirillum brasilens e*[18] have been mined in the genome of *Pantoea*. PAA have been shown to possess a weak auxin activity and antimicrobial activity against both bacteria and fungi. The *paaABC* operon in the genomes of *Azospirillum* and *B. simplex* that is important for PAA degradation was also mined in this study [18].

ACC deamination

The genes *acdS*, encoding the ACC deaminase structural genes, *acdR*, encoding leucine responsive regulatory protein (LRP) and *yedO* of *Pseudomonas putida* GR12-2 were used as query for homology search of the mined *Pantoea* genomes [19].

Nitric oxide (NO) production

The copper nitrite reductase gene *nirBDK* and *niiA* of *Azospirillum brasilense* Sp245, that leads to NO formation and therefore root branching in plants, was used in homology searches of the targeted *Pantoea* genomes [1].

Polyamines biosynthesis

Polyamines such as spermine, spermidine and putrescine have been shown to confer PGP properties in *B. subtilis* OKB105 [20]. Therefore, homology to genes involved in polyamine synthesis such as *speA* (agmatine synthesis), *speB*, (putrescine synthesis); and *speE*, *speD* (spermidine synthesis), *speG*, *speF* were examined in the genome of *Pantoea.* The gene *metK*, responsible for the conversion of methionine to S-adenosyl-methionine, was also searched in the *Pantoea* genome. Additionally, a genome survey for genes involved in the biosynthesis of

polyamines binding proteins, permeases, and transporters was also conducted by keyword search.

Plant protection from oxidative stress

Antioxidant enzymes

All enzymes active against oxidative stress mainly superoxide dismutases (*sodA*, *sodC* and *sodF*), hydrogen peroxide decomposing catalases (*katA*, *katE*, katG and *katX*), manganese catalase (*ydbD*), three alkyl hydroperoxide reductases (*ahpC* and *ahpF*), thiol peroxidase (*tpx* and *bcp*), glutathione peroxidase (*gpo* and *btuE*), bacillopeptidase F (*bpr*), gamma-glutamyl transpeptidase (*ggt*), and an operon (*ohrARB*and *AL522_04860*) for resistance to organic peroxides described by Niazi et al. [6] in *B. velezensis* UCMB5113 have been used as candidates for homology based searches in the *Pantoea* genomes. Two other sets of genes have also been mined in the targeted genomes, the genes *gacS*, *soxS*, *soxR* and *oxyR* involved in plant protection against oxidative stress [21] and the *hmpA* gene encoded by the genome of *B. velezensis* UCMB5113 and encoding flavohemoprotein nitric oxide dioxygenase [22].

Plant induction of disease resistance

Salicylate biosynthesis

Homologs of *pchBA* genes of *Pseudomonas aeruginosa* genes have been used as a bait in blast searches in the different *Pantoea* genomes.

Acetoin and 2,3-butandiol biosynthesis

Acetoin biosynthesis in the collection of *Pantoea* strains have been checked using *B. velezensis* SQR9 genes encoding acetoin biosynthesis: acetolactate synthase *alsS* (E.C. 2.2.1.6), acetolactate decarboxylase *alsD* (E.C. 4.1.1.5), *ilvGHI* operon and the regulatory gene *alsR* as well as the gene *bdhA* encoding 2,3-butanediol dehydrogenase encoding 2,3-butandiol biosynthesis [5] in homology-based searches.

Antibiotics and related compounds

Hydrogen cyanide (HCN) production

HCN encoding genes were mined using the *Pseudomonas* spp. *hcnABC* operon [1].

2,4-diacetylphloroglucinol production

Blast searches have been conducted using *phl*ACBD *Pseudomnonas fluorescens* genes to collect putative homologous sequences in the genomes of the mined *Pantoea* strains [1].

γ-aminobutyric acid (GABA) Biosynthesis

GABA biosynthesis ability of the different *Pantoea* strains targeted have been studied using homology search approaches with baits consisting of *gabD* and *gabT* involved in production of pest/disease suppressing γ-aminobutyric acid (GABA) [23].

Resistance to drugs and heavy metals

Tetracycline resistance

Proteins contributing to tetracycline resistance *tetB* and transcriptional regulator tetR in *Bacillus subtilis* [24] have been used as bait in blast searches of the *Pantoea* genomes collection. Homologs of the multifunctional tetracycline-metal/H^+ antiporter (*tetA*) have also been mined [25].

Streptothricin resistance

Homologs of the streptothricin acetyltransferase encoding operon *yyaACDEHJKLRST* [26] have been targeted by blast search.

Fosfomycin resistance

Fosfomycin resistance was checked in the genomes of the strains of *Pantoea* using either keyword search approach or through blast search using the *Bacillus cereus fosB* gene [27].

Bleomycin resistance

Glyoxalase/Bleomycin resistance genes present in the genomes of the different *Pantoea* strains have been recovered either using key word search or by looking for homologs to the *Bacillus licheniformis* Glyoxalase/Bleomycin resistance gene *ykcA* and *phnM* in blast search [28].

Penicillin resistance

β-lactamase genes present in the genome of the *Pantoea* strains collection have been identified by key word search in the genome as well as homology-based searches using *Bacillus subtilis* (strain 168) β-lactamase gene *penP* and *ampCDH* operon as a bait in the blast search [29].

Quinolone resistance

The different genomes have been submitted to a targeted search of homologs of the *Staphylococcus norA* gene [30].

Chloramphenicol/florfenicol resistance protein

Escherichia coli gene *floR*, AL522_16210 and BEE12_19240 locus have been used as a bait for targeted search of homologs in the *Pantoea* genomes mined [31].

Aminoglycoside 6-adenylyltransferase

Genes encoding homologs of the aminoglycoside 6-adenylyltransferase gene of *Bacillus subtilis* 168 (*aadK*) have been mined in the different strains targeted [32].

Aminoglycoside phosphotransferase

Aminoglycoside phosphotransferase has been searched in the genome of the *Pantoea* strain collection either by keyword search in the genome or by homology search using *Bacillus subtilis ycbJ* and *ycfN* genes [33].

Antibiotic efflux ABC transporter (ATP-binding protein)

Antibiotic efflux ABC transporters (*ycfUVW*, *yadH*, *mdlAB*, *cydCD*, *msbA* and *yojI*) have been identified either through keyword search in the different *Pantoea* genomes mined or by using *Bacillus subtilis vmlR* encoding antibiotic efflux ATP-binding transport gene as a bait [34].

Wide range drugs and toxic compounds resistance through multidrug exporters

Putative multidrug exporters' genes have been identified from the different *Pantoea* genomes using either keyword searches or according to Niazi et al. [6].

Arsenic detoxification

Arsenic detoxification was studied using either keyword search for arsenic resistance or through homology search using the genes *arsABC*, *ywrK*, *yffB*, *yfgD* as a bait [35].

Copper resistance

The *copYZABD* operon encoding a major copper resistance mechanism as well as one-component regulators *cueR* and *csoR*, identified in *Escherichia coli*, *Enterococcus hirae* and *Mycobacterium*

tuberculosis, respectively [36] and *ctpAB* and *ycnJ* genes encoding copper resistance proteins [37] were used in BLAST searches for homologs in the *Pantoea* genome collection.

Aluminum resistance

ynbB gene of *Bacillus subtilis* strain 168 have been used as a bait in blast search for homologs in the *Pantoea* genome collection [29].

Camphor resistance

Escherichia coli crcAB, *cspE*, and *yhdV* have been used as a bait in blast search for homologs in the different *Pantoea* targeted genomes [38].

Tellurite resistance

Bacillus anthracis yceGH, *yaaN*, *tehB* and *mae* have been targeted in a blast homology search in the *Pantoea* genome collection [39].

Cation Diffusion Facilitator Transporters *CzcD*

Bacillus subtilis CzcD gene encoding cadmium, cobalt and zinc/H(+)-K(+) antiporter that protects the cell against elevated levels of Zn(II), Cu, Co(II) and Ni(II) has been used as a bait in a blast search of homology in the *Pantoea* selected genomes [40].

Metalloregulators family *Fur*, *ArsR*, *MerR*, *NikR*, *DtxR*, *mtnR* and *yfmP*

Homologs of all sensors *Fur*, *ArsR*, *MerR*, *NikR*, *DtxR*, *mtnR* and *yfmP* in the *Pantoea* genome collection have been searched using *Bacillus subtilis* sequences [41].

Toxin-Antitoxin Module response to stress

Bacillus subtilis sequences encoding the toxin/antitoxin system gene s*ndoA* (*ydcE*) and *ndoAI* (*ydcD*) have been used to mine the *Pantoea* genome collection selected [42].

Degradation of aromatic compounds

Resistance to phenolic acids

Phenolic acid decarboxylases of *B. subtilis padC*, *bsdBCD* (*yclBCD*), *pdc*, BEE12_10480 and AL522_07735 allowing resistance to phenolic acids such as Vanillate, 4-hydroxybenzoate, salicylic, ferulic and *p*-coumaric acids considered as natural toxins have been mined. The putative LysR-type regulator, encoded by *bsdA* (*yclA*), *cysB* and *cbl* genes and encoding the transcriptional activator of *bsdBCD* expression in response to phenolic acids [43] was also used in the study.

Degradation of sulfur-containing organic molecules

The operon *dszABC* of *Rhodococcus* sp. [44] active against the model compound Dibenzothiophene (DBT) was used as a bait in homology-based searches in the genome of the *Pantoea* collection.

2-Methylhydroquinone and catechol resistance

Bacillus velezenzis FZB42 *azoR2* and *mhqADNOPE* gene homologs have been searched in the *Pantoea* collection [45].

Supplementary Table S1. Soil physicochemical characteristics. Mean of three repetitions ± standard error represents the data presented.

	pH	Ec (mS/cm)	$CaCO_3$ (%)	Carbon (%)	Organic matter (%)
Soil	9 ± 0.05	3.57 ± 0.01	22.00 ± 0.57	0.39 ± 0.02	0.68 ± 0.02

Supplementary Table S2. Plant growth promoting activities of 22 bacteria selected in this study.

Strains	P2O5 µg/ml	IAA µg/ml	SID%	HCN	NH3	ACC	N2
**D1**	30.31±2.20	83.41±1.29	12.7±0.30	-	+	-	+
**D2**	49.9±1.10	8.25±1.07	10.2±0.40	-	+	-	+
**D3**	55.44±5.40	112.5±2.50	8.36±0.13	-	+	-	+
**D5**	17.95±0.50	10.33±1.87	5.96±0.76	-	-	-	+
**B1**	105.81±1.41	104.25±0.55	14.7±0.30	-	+	-	+
**B2**	102.46±0.56	97.58±2.44	3.20±0.40	-	+	-	+
**B3**	150.76±1.50	99.75±0.05	51.3± 0.50	-	+	+	+
**B4**	39.54±2.53	23.91±0.55	30.5± 0.50	-	-	-	+
**B5**	176.51±16.08	102.58±0.91	46.36±0.46	-	+	+	+
**B6**	67.7±3.30	203.33±1.13	15.26±0.26	-	+	-	+
**B9**	112.31±2.89	175±12.60	10.54±0.24	-	+	-	+
**B10**	15.91±0.95	21.33±0.83	9.66±0.59	-	+	-	+
**B12**	67.5±0.51	11.01±0.89	18.36±0.36	+	+	-	+
**O1**	28.5±1.50	13.16±0.06	6.26±0.46	-	+	-	+
**O2**	115.89±0.88	7.08±0.30	40.96± 0.96	-	-	-	+
**O3**	91.7±1.31	11.33±0.30	11.35±0.45	-	+	-	+
**O4**	105.7±2.00	12.33±0.23	50.36± 0.36	+	+	+	+
**O6**	94.14±0.99	4.16±0.34	10.75±075	-	+	-	+
**O8**	115.97±1.08	4.83±0.33	28.96±024	-	+	-	+
**O9**	102.43±4.07	16.66±0.67	16.38±0.33	-	+	-	+
**O10**	72.6±1.80	10.31±0.71	5.22±0.42	-	+	+	+

Supplementary Table S3. PGP activities of *Pantoea agglomerans* strain Pa.

Direct Plant Growth Promotion		Indirect Plant Growth Promotion	
Nitrogen Fixation	+	Chitinase Production	+
Phosphate Solubilization	223.12±48.08 μg/mL	Protease Production	+
		Cellulase Production	+
Siderophore Production	40.8±8.09%	ACC deaminase Production	+
Indole Acetic Acid Production	99.15±0.67 μg/mL	NH_3 Production	+
		HCN Production	-

Supplementary Table S4. Compounds identified in Pa volatile fraction by GC-MS analysis

Rt	KI*	LS**	Tentative identification	Structure of the molecule	PGP effectiveness or biocontrol ability	Reference
11.76	1630	90.9	Indole-3-acetic acid methyl ester	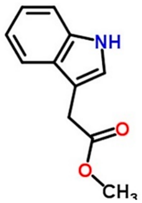	Plays an important role in leaf development, Integration of auxin/indole-3-acetic acid 17 and RGA-LIKE3 confers salt stress resistance through stabilization by nitric oxide in Arabidopsis	[46, 104]
24.57	1773	93.1	Indole-3-acetamide	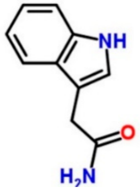	A molecule related to auxin biosynthesis and growth of plants	[47]
26.63	1942	88.7	Palmitic acid	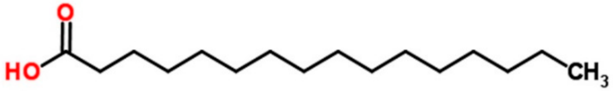	Enhances the growth of the seedlings and effects against sproe germination and mycelial growth of phytopathogenic fungi, In salt resistance Spartina patens plant, membrane fatty acid remodeling and could contribute to the resistance of this plant to salt. A significant increase in the relative amounts of the saturated fatty acids (SFA) was observed, namely palmitic acid, essential for PS II functioning, and stearic (C18:0) acid during incresing levels of salinity.	[48, 105]

Supplementary Table S4. Continued.

Rt	KI*	LS**	Tentative identification	Structure of the molecule	PGP effectiveness or biocontrol ability	Reference
32.22	2095	90.7	Linoleic acid	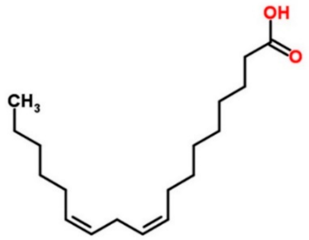	Salinity stress triggers the biosynthesis of fatty acids, such as linolenic acid, responsible for adaptation and growth development of rice plants in response to salinity stress.	[106]
32.42	2113	86.9	Oleic acid	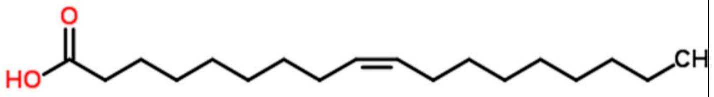	Enhanced the growth of the seedlings and is effective against phytopathogenic fungi	[48]

* Kovat’s Index. ** Lib Score

Supplementary Table S5. Comparison of chemical profiles of Pa under OSMAC by LC-HRESIMS

Index	Rt (min)	m/z $[M+H]^+$	Molecular formula	Tentative Identification	Structure	PGP effectiveness or biocontrol ability	M1	M2	M3	M4	M5	M6	Reference
1	4.52	445.1315	$C_{23}H_{24}O_7S$	New thiazole derivative			×	×	×	×	×	✓	
2	5.02	235.1082	$C_{12}H_{14}N_2O_3$	Cyclo(Phe-Ser)	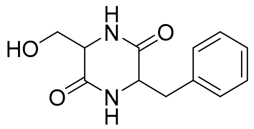	Significant inhibition against different phytopathogenic fungi	✓	×	×	×	×	×	[49]
3	5.02	247.1083	$C_{13}H_{14}N_2O_3$	N-Acetyl tryptophan	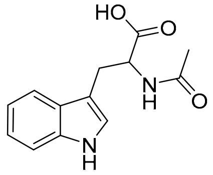	Neuroprotective activity in vivo, Under stress, melatonin usually accumulates sharply by modulating its biosynthesis and metabolic pathways. Beginning from the precursor tryptophan, four consecutive enzymes mediate the biosynthesis of tryptamine or 5-hydroxytryptophan, serotonin, N-acetylserotonin or 5-methoxytryptamine, and melatonin	✓	×	×	×	×	×	[50, 107]
4	5.10	261.18533	$C_{14}H_{16}O_3N_2$	cyclo(Pro-Tyr)	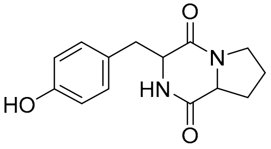	Antibacterial agent against MRSA and *E. coli*, moderate inhibition against hepatitis C protease	✓	✓	×	×	×	×	[51, 52]

5	5.10	194.0817	$C_{13}H_{14}N_2O_3$	Spoxazomicin C	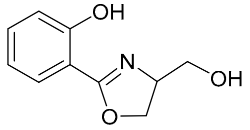	Good anti-trypanosomal agent	×	✓	×	×	×	×	[53]
6	5.11	205.0976	$C_{11}H_{12}N_2O_2$	Cyclo(Gly-Phe)	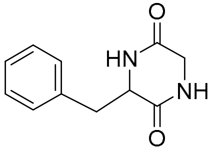	Antimicrobial and biofilm inhibiting agent	✓	×	×	×	×	×	[54]
7	5.20	217.0977	$C_{12}H_{12}N_2O_2$	Sannanine	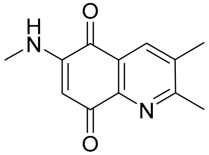	Moderate antitumor activity	✓	×	×	×	×	×	[55]
8	5.40	197.12887	$C_{10}H_{16}N_2O_2$	cyclo(Pro-Val)	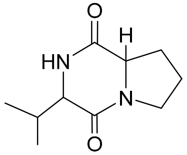	Mild anticancer activity in vitro	✓	×	×	×	×	×	[56]
9	5.48	259.1080	$C_{14}H_{14}N_2O_3$	Pyridindolol	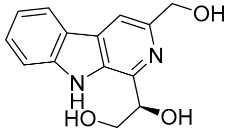	Antimicrobial agent	×	✓	×	×	×	×	[57]
10	5.58	185.1287537	$C_9H_{16}N_2O_2$	cyclo(Ala-Leu)	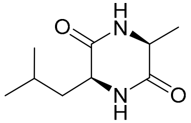	Antifoulingagent	✓	×	×	×	×	×	[58]
11	5.80	227.1395645	$C_{11}H_{18}N_2O_3$	cyclo-4-hydroxyPro-Leu	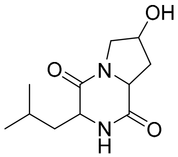	Anti-proliferative against human glioma cells	✓	✓	×	×	×	×	[59]
12	5.81	160.07580	$C_{10}H_9NO$	4-(Hydroxymethyl)quinoline	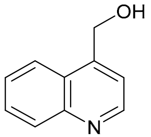	Anti-schistasomal activity	×	✓	×	×	×	×	[60]

13	6.09	214.1442	$C_{11}H_{19}NO_3$	Pyrrolam D	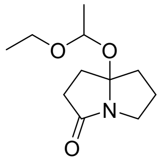	Possible herbicidal activity	×	✓	×	×	×	×	[61]
14	5.94	211.1446	$C_{11}H_{18}O_2N_2$	Cyclo(Ile-Pro)	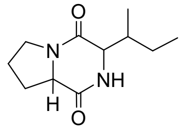	Moderate antibacterial activity	✓	×	✓	×	×	×	[62]
15	6.20	204.1023	$C_{12}H_{13}NO_2$	Actinopolymorphol B	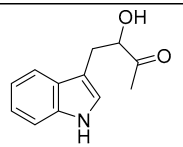	Cancer chemoprevetion activity As an oestrogen receptor heterodimerizing	×	×	✓	×	×	×	[63]
16	6.90	219.1133	$C_{12}H_{14}N_2O_2$	Cyclo(Ala-Phe)	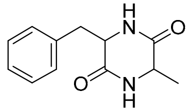	Moderate antibacterial activity	✓	×	×	×	×	×	[62]
17	6.90	277.1551	$C_{15}H_{20}N_2O_3$	Cyclo(Leu-Tyr)	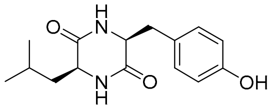	Moderate antibacterial activity	✓	×	×	×	×	×	[62]
18	7.53	157.0958	$C_7H_{12}N_2O_2$	Cyclo(Val-Gly)	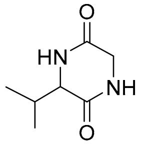	Moderate antimalarial activity	×	×	×	×	×	✓	[64]
19	8.23	169.0959	$C_8H_{12}N_2O_2$	cyclo-(Ala,Pro)	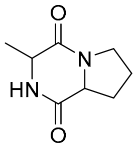	Moderate antifungal and antibacterial activity	×	×	×	×	✓	×	[65]
20	7.12	239.0820	$C_{14}H_{10}N_2O_2$	6-Carboxymethyl-phenazine	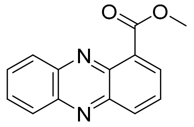	Moderate antifungal Activity against different plant pathogenic fungi	✓	×	×	×	×	×	[66]

21	7.76	176.0708	$C_{10}H_9NO_2$	4-Hydroxy-N-methylcarbostyril	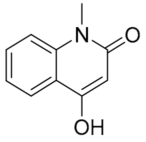	Mild antimitotic activity. Neither cytotoxic nor antimicrobial.	✓	×	×	×	×	×	[67]
22	8.63	188.1074	$C_{12}H_{13}NO$	2-alkyl-4-quinolone	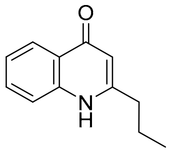	Membrane vesicle stimulant in *Pseudomonas* sp.	×	×	✓	×	×	×	[68]
23	8.74	245.1289	$C_{14}H_{16}N_2O_2$	cyclo-(Pro-Phe)	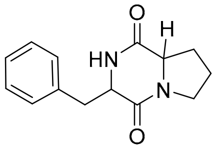	cell-cell signaling effect	✓	×	×	×	×	×	[69]
24	9.49	327.1216	$C_{19}H_{18}O_5$	WS-1128-C	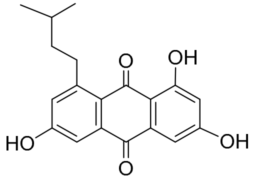	A potent estrogen receptor agonist	×	✓	×	×	×	×	[70]
25	9.61	190.0866	$C_{11}H_{11}NO_2$	3-Indole propionic acid	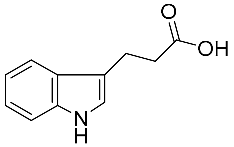	Messenger molecules in bacteria to support effective communication for symbiotic relationships	×	✓	×	×	×	×	[71]
26	9.69	311.1394	$C_{18}H_{18}N_2O_3$	4'-Methoxycyclopeptin	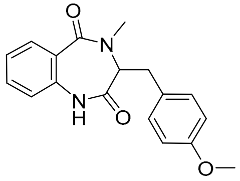	Weak insecticidal activity	✓	×	×	×	×	×	[72]
27	10.00	286.1554	$C_{16}H_{19}N_3O_2$	Cyclo(D-Val-L-Try)	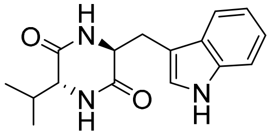	Bioactivity not reported	×	✓	×	×	×	×	[73]

28	10.22	213.1602	$C_{11}H_{20}N_2O_2$	cyclo(L-Ile-L-Valine)	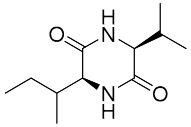	Moderate antibacterial activity	✓	×	×	×	×	×	[62]
29	10.69	300.1712	$C_{17}H_{21}N_3O_2$	Cyclo(Leu-Try)	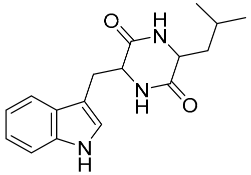	Antioxidant activity	×	✓	×	×	×	×	[74]
30	11.48	178.0324	C_9H_7NOS	2-(2-Hydroxyphenyl) thiazole	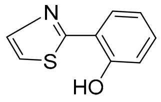	The up-accumulation of thiazoles might help to restore DNA stability and alleviate oxidative stress caused by NaCl treatment in *Brassica napus* leaves. DNA damage repair and stress-tolerance mechanisms has been observed as its role in bacteria, yeast and *Arabidopsis thaliana*	✓	✓	✓	×	×	×	[75, 108]
31	11.52	224.0381	$C_{10}H_9NO_3S$	(+)-(S)-dihydroaeruginoic acid	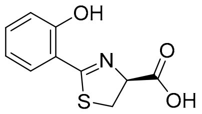	Bioactivity not reported	✓	×	×	×	×	×	[76]
32	11.62	204.1025	$C_{12}H_{13}NO_2$	Actinopolymorphol B	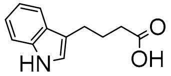	Potential anticancer activity as tubulin polymerization inhibitors	×	×	✓	×	×	×	[77]
33	12.61	218.1182	$C_{13}H_{15}NO_2$	**New quinolinederivative**			✓	×	×	×	×	×	
34	12.55	274.1445	$C_{16}H_{19}NO_3$	3-(2'-Acetoxy-3'-oxo-4'-methylpentyl)-indole	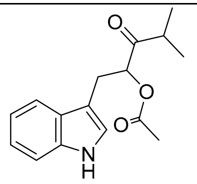	Potential anticancer activity as tubulin polymerization	×	×	✓	×	×	×	[78]

35	12.65	261.1603	$C_{15}H_{20}N_2O_2$	Cyclo(Leu-Phe)	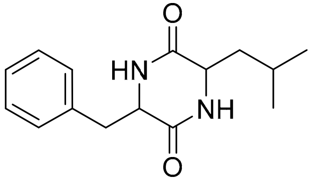	Mild radical scavenging activity	✓	×	×	×	×	×	[74]
36	13.18	214.1232	$C_{14}H_{15}NO$	New quinoline derivative			✓	×	×	×	×	×	
37	13.40	295.1448	$C_{18}H_{18}N_2O_2$	cyclo-(L-Phe-L-Phe)	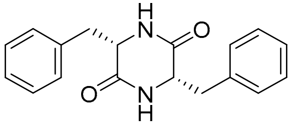	Good anthelmintic activity	✓	×	×	×	×	×	[78]
38	13.49	216.1389	$C_{14}H_{17}NO$	Pseudane-V	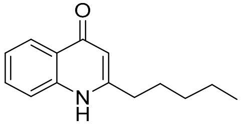	Potential herbicide as an inhibitor of Photosystem II	✓	×	×	×	×	×	[79]
39	13.51	276.1602	$C_{16}H_{21}NO_3$	3-n-Heptyl-3-hydroxy-(1H,3H)-quinoline-2,4-dione	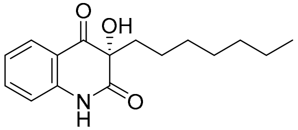	A potent anti-asthmatic and anti-inflammatory agent as 5-Lipoxygenase inhibitors.	✓	✓	✓	×	×	×	[80]
40	13.62	284.1651	$C_{18}H_{21}NO_2$	New quinoline derivative			✓	×	×	×	×	×	
41	14.01	226.1231	$C_{15}H_{15}NO$	(E)-4-Phenyl-3-(pyridine-2-yl)but-2-en-1-ol	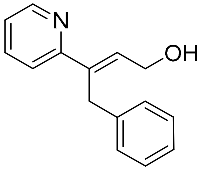	A potent antiproliferative agent	✓	×	×	×	×	×	[81]
42	14.06	228.1389	$C_{15}H_{17}NO$	New quinolinederivative			✓	×	×	×	×	×	
43	14.49	211.0872	$C_{13}H_{10}N_2O$	Cyanomycin	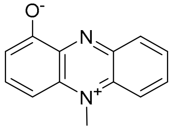	An antibiotic	✓	×	×	×	×	×	[82]

44	14.79	228.1389	C15H17NO	New quinolinederivative			✓	×	×	×	×	×	
45	14.79	300.1600	$C_{18}H_{21}NO_3$	Streptoverticillin	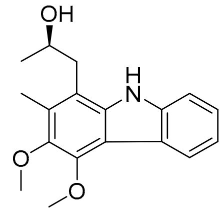	Good antifungal activity against *Peronophythora litchii*	×	✓	×	×	×	×	[83]
46	14.84	178.0325	C_9H_9NOS	(R)-5-phenyloxazolidine-2-thione	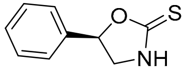	Tyrosinases inhibitor	×	×	✓	×	×	×	[84]
47	14.88	274.1444	$C_{16}H_{19}NO_3$	New quinoline derivative			✓	×	×	×	×	×	
48	14.88	323.0530	$C_{14}H_{16}N_2O_3S_2$	Pyochelin	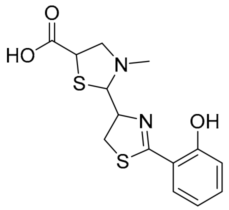	Transporting and shuttling biologically relevant metals (Fe(III), Cu(II) or Zn(II))	✓	×	×	×	×	×	[85]
49	14.90	160.0428	$C_6H_9NO_2S$	Citiolone	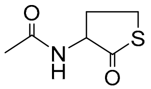	Oxygen free radical scavenger	×	✓	×	×	×	×	[86]
50	14.91	274.1444	$C_{16}H_{19}NO_3$	3-(2'-acetoxy-3'-oxo-4'-methylpentyl)-indole	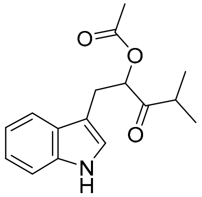	Potential anticancer activity as tubulin polymerization	×	✓	×	×	×	×	[77]
51	15.26	194.15454	$C_{12}H_{19}NO$	**New quinoline derivative**			×	✓	×	×	×	×	

52	15.52	232.1339	$C_{14}H_{17}NO_2$	3-(2'-Hydroxy-3'-oxo-4'-methylpentyl)-indole	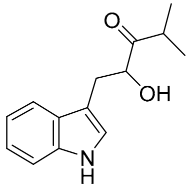	Potential anticancer activity as tubulin polymerization	×	✓	×	×	×	×	[77]
53	15.70	274.1809	$C_{17}H_{23}NO_2$	Lorneamide A	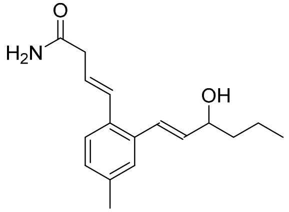	Antibacterial against *Bacillus subtilis*	✓	×	✓	×	×	×	[87, 88]
54	15.81	325.0679	$C_{14}H_{16}N_2O_3$	cyclo(4-OH-Pro-Phe)	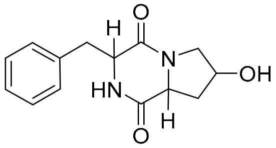	Good Acaricidal activity	✓	×	×	×	×	×	[89]
55	16.05	286.1812	$C_{18}H_{23}NO_2$	Dienomycin-B	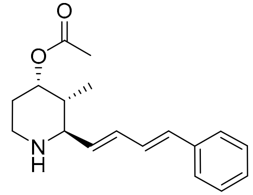	Anti- bacterial activity	×	✓	×	×	×	×	[90]
56	16.07	246.1494	$C_{15}H_{19}NO_2$	New quinoline derivative			✓	×	×	×	×	×	
57	16.33	244.1338	$C_{15}H_{17}NO_2$	New quinoline derivative			✓	×	×	×	×	×	
58	16.54	242.1548	$C_{16}H_{19}NO$	2-Heptenyl-4-quinolinon	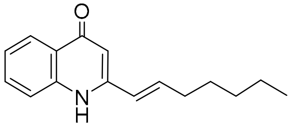	Antimalarial and antioxidant activity	✓	✓	×	✓	×	×	[91]

59	16.87	274.1444	$C_{16}H_{19}NO_3$	€-3-(hept-1-en-1-yl)-3-hydroxyquinoline-2,4(1H,3H)-dione	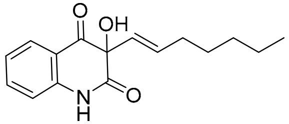	Bioactivity not reported, possible antimalarial and anticancer agent	✓	×	×	✓	×	×	[91]
60	17.01	651.2722	$C_{30}H_{41}O_{13}N_3$	**New quinoline derivative**			×	×	×	✓	×	×	
61	17.06	244.1700	$C_{16}H_{21}NO$	2-n-Heptyl-(1H)-quinolin-4-one	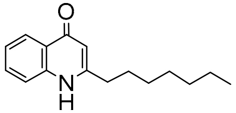	A potent inhibitor of some cytochrome-mediated electron-transport systems	✓	✓	×	×	×	✓	[92]
62	17.49	176.0693	$C_{10}H_9NO_2$	4-Hydroxy-N-methylcarbostyril;	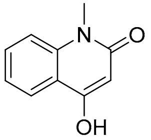	Not either antibacterial or antimitotic	×	×	×	✓	×	×	[93, 94]
63	17.53	162.0536	$C_9H_7NO_2$	Indole-3-carboxylic acid	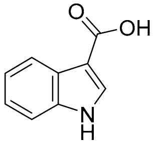	Significant antibacterial and anthelmintic activity	×	×	×	✓	×	×	[95]
64	18.03	174.0552	$C_{10}H_7NO_2$	4-Quinolinecarboxylic acid	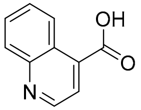	Highly potent alkaline phosphatase inhibitor	×	×	✓	×	×	×	[96]
65	18.32	260.1654	$C_{16}H_{21}NO_2$	2-Heptyl-3-hydroxy-4(3H)-quinolone	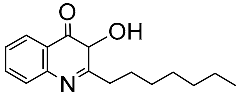	A signal molecule in cell-cell communication system of *Pseudomonas aeruginosa*; Control of the expression of *lasB*, which encodes for the major virulence factor, *LasB*elastase	✓	✓	✓	×	×	×	[97]
66	18.32	329.1923	$C_{20}H_{25}NO_3$	**New quinoline derivative**			×	✓	×	×	×	×	
67	18.36	298.1394	$C_{13}H_{19}N_3O_5$	**New quinoline derivative**			×	×	×	×	×	✓	

68	18.63	260.2015	$C_{17}H_{25}NO$	Phormidinine A	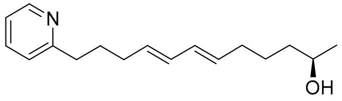	Bioactivity not reported	×	×	✓	×	×	×	[98]
69	18.91	258.1859	$C_{17}H_{23}NO$	2-octyl-4(1H)-quinolone|2-octyl-4-quinolone	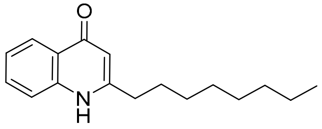	Moderate antimalarial activity	×	×	×	✓	×	×	[91]
70	18.92	260.16321	$C_{16}H_{21}O_{2}N$	**New quinoline derivative**			×	×	×	×	×	✓	
71	18.97	158.0599	$C_{10}H_{7}NO$	Quinoline-4-carbaldehyde	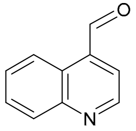	Anti-asthmatic activity	×	×	×	✓	×	×	[99]
72	18.97	172.0755	$C_{11}H_{9}NO$	N-Benzoyl-Pyrrole	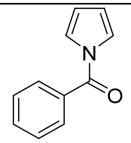	Potential anti-inflammatory activity	×	×	×	✓	×	×	[100]
73	19.43	272.1645	$C_{17}H_{21}NO_{2}$	**New quinoline derivative**			✓	×	×	✓	×	×	
74	19.14	272.1647	$C_{17}H_{21}NO_{2}$	**New quinoline derivative**			✓	×	×	✓	×	×	
75	19.19	274.1805	$C_{17}H_{23}NO_{2}$	4-hydroxy-2-octylquinoline 1-oxide	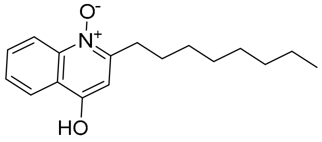	Moderate anti-malarial, antibacterial, and anti-oxidant activity	✓	✓	×	×	×	×	[91]
76	19.63	270.1861	$C_{18}H_{23}NO$	2-(n-delta(1)-Nonenyl)-4-oxyquinoline	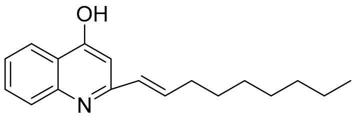	Moderate antimalarial activity	✓	✓	×	×	×	×	[91]
77	19.62	270.1861	$C_{18}H_{23}NO$	Burkholone	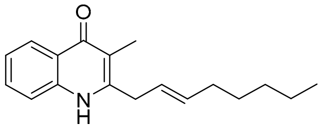	Cytotoxic antibiotic	×	×	✓	×	×	×	[101]

78	19.84	272.2017	$C_{18}H_{25}NO$	1. 2-Nonyl-quinonyl-4-ol	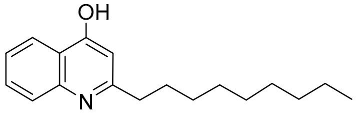	Moderate antimalarial activity	✓	✓	×	×	×	×	[91]
79	19.7	358.2380	$C_{22}H_{31}NO_3$	**New quinoline derivative**			✓	×	×	×	×	×	
80	19.72	324.15463	$C_{21}H_{23}OS$	New thiazole derivative			×	×	×	×	×	✓	
81	19.93	356.2224	$C_{22}H_{29}NO_3$	**New quinoline derivative**			✓	×	×	×	×	×	
82	20.15	277.1172	$C_{16}H_{22}O_4$	Oncorhyncolide	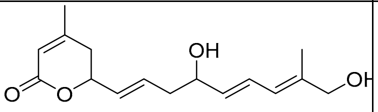	Potential anticancer activity	✓	✓	✓	×	×	×	[101, 102]
83	20.16	286.1809	$C_{18}H_{23}NO_2$	**New quinoline derivative**			✓	✓	×	×	×	✓	
84	20.89	288.1965	$C_{18}H_{25}NO_2$	4-Hydroxy-2-n-nonanyl-quinoline N-oxide	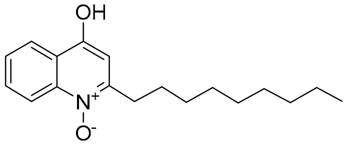	Moderate antimalarial activity	✓	✓	×	✓	×	✓	[91]
85	21.21	330.2066	$C_{20}H_{27}NO_3$	**New quinoline derivative**			×	×	✓	×	×	×	
86	21.59	312.1594	$C_{19}H_{21}NO_3$	New quinoline derivative			×	×	✓	×	×	×	
87	21.67	314.2118	$C_{20}H_{27}NO_2$	(Z)-4-hydroxy-2-(undec-4-en-1-yl) quinoline 1-oxide	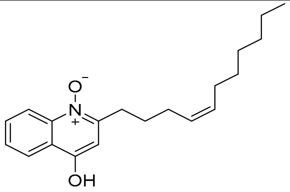	Antibacterial, antimalarial and mild anti-tubercular activity	✓	✓	✓	✓	×	×	[91]

88	21.83	300.2322	$C_{20}H_{29}NO$	2-Undecyl-4(1H)-quinolinone; 2-Undecylquinolone	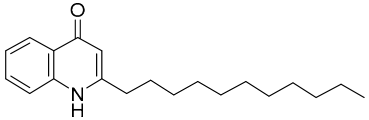	Potential algicidal activity	×	✓	×	×	×	×	[103]
89	21.90	202.0869	$C_{12}H_{11}NO_2$	**New quinoline derivative**			×	×	×	×	×	✓	
90	22.02	352.2274	$C_{23}H_{29}NO_2$	**New quinoline derivative**			×	×	✓	×	×	×	
91	22.23	324.2325	$C_{22}H_{29}NO$	**New quinoline derivative**			×	✓	×	×	×	×	
92	22.39	326.2482	$C_{22}H_{31}NO$	**New quinoline derivative**			×	×	✓	×	×	×	
93	22.53	340.22730	$C_{22}H_{29}NO_2$	**New quinoline derivative**			✓	✓	×	×	×	×	
94	22.65	316.2277	$C_{20}H_{29}NO_2$	**New quinoline derivative**			✓	✓	×	×	×	×	
95	22.93	342.2426	$C_{22}H_{31}NO_2$	**New quinoline derivative**			×	✓	×	×	×	×	

Supplementary Table S6. Secondary metabolite clusters of *Pantoea agglomerans* and *P. vagans* strains used in this study.

Strain clusters	Type	Length (bp)	Most similar known clusters	Predicted core clusters
**Pa**				
Cluster 1	Aryl polyene	43586	APE Ecbiosynthetic gene cluster (78% of genes show similarity)	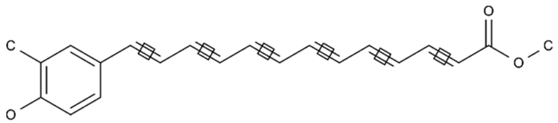
Cluster 2	Hserlactone	20611	-	-
Cluster 3	Thiopeptide	26257	O-antigen biosynthetic gene cluster (14% of genes show similarity)	-
Cluster 4	Terpene	23561	Carotenoid biosynthetic gene cluster (100% of genes show similarity)	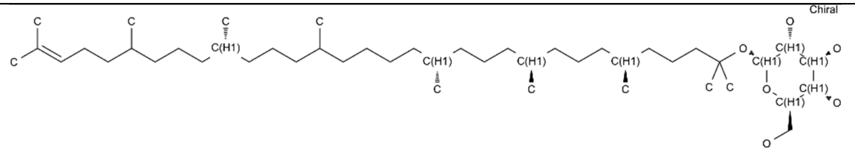
Cluster 5	Siderophore	12354	Desferrioxamine B biosyntheticgene cluster (40% of genes show similarity)	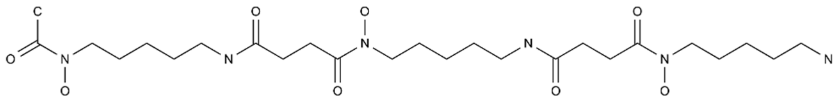
Cluster 6	Hserlactone	20638	-	-
Cluster 7	Nrps	52063	Turnerbactin biosynthetic gene cluster (30% of genes show similarity)	-

Supplementary Table S6. Continued.

Strain clusters	Type	Length (bp)	Most similar known clusters	Predicted core clusters
**299R**				
Cluster 1	Thiopeptide	26255	-	-
Cluster 2	Hserlactone-Aryl polyene	69839	APE Ecbiosynthetic gene cluster (78% of genes show similarity)	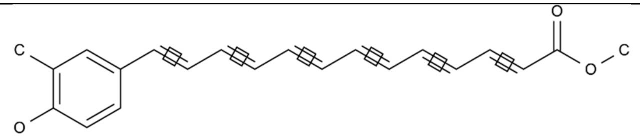
Cluster 3	Terpene	23561	Carotenoid biosynthetic gene cluster (100% of genes show similarity)	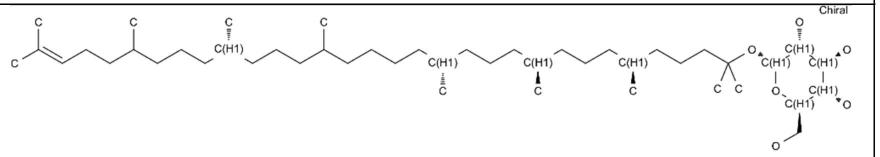
Cluster 4	Siderophore	12354	Desferrioxamine B biosynthetic gene cluster (60% of genes show similarity)	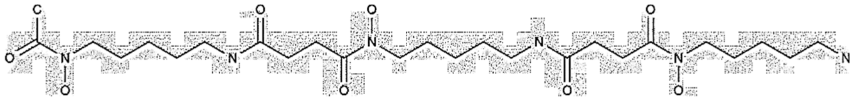

Supplementary Table S6. Continued.

Strain clusters	Type	Length (bp)	Most similar known clusters	Predicted core clusters
**IG1**				
Cluster 1	Thiopeptide	26256	-	-
Cluster 2	Hserlactone-Aryl polyene	69838	APE Ecbiosynthetic gene cluster (78% of genes show similarity)	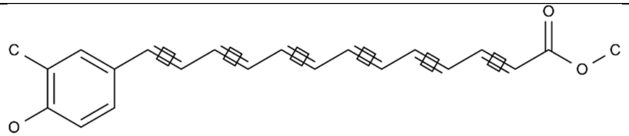
Cluster 3	Siderophore	12354	Desferrioxamine B biosynthetic gene cluster (40% of genes show similarity)	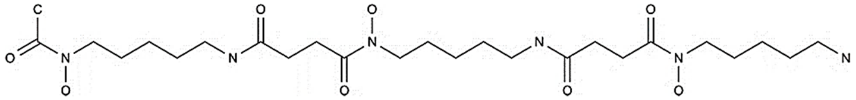
Cluster 4	Terpene	23561	Carotenoid biosynthetic gene cluster (100% of genes show similarity)	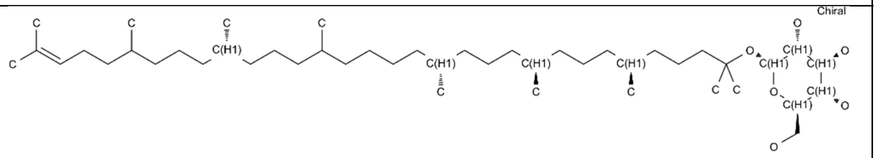
Cluster 5	Nrps	44500	Turnerbactin biosynthetic gene cluster (30% of genes show similarity)	-

Supplementary Table S6. Continued.

Strain clusters	Type	Length (bp)	Most similar known clusters	Predicted core clusters
**NBRC_102470**				
Cluster 1	Thiopeptide	26256	-	-
Cluster 2	Hserlactone	20611	-	-
Cluster 3	Aryl polyene	43586	APE Ecbiosynthetic gene cluster (84% of genes show similarity)	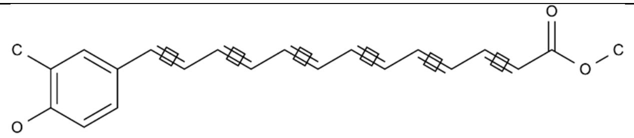
Cluster 4	Siderophore	11848	Desferrioxamine B biosynthetic gene cluster (40% of genes show similarity)	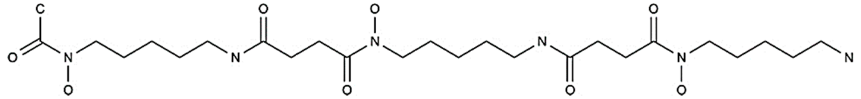
Cluster 5	Terpene	23561	Carotenoid biosynthetic gene cluster (100% of genes show similarity)	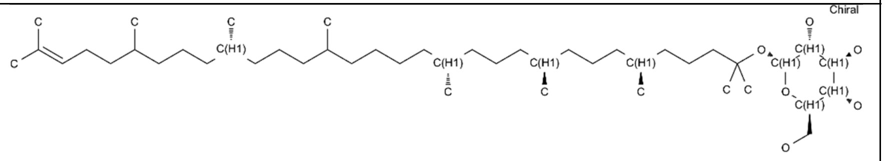
Cluster 6	Nrps	52063	Turnerbactin biosynthetic gene cluster (30% of genes show similarity)	-
Cluster 7	Hserlactone	14148	-	-

Supplementary Table S6. Continued.

Strain clusters	Type	Length (bp)	Most similar known clusters	Predicted core clusters
**824-1**				
Cluster 1	Thiopeptide	26256	-	-
Cluster 2	Hserlactone-Aryl polyene	69840	APE Ecbiosynthetic gene cluster (78% of genes show similarity)	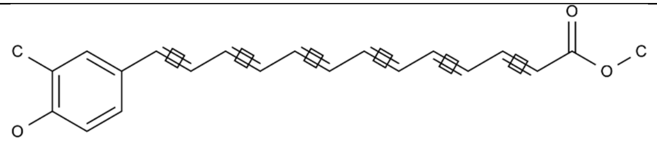
Cluster 3	Hserlactone	20638	-	-
Cluster 4	Nrps	52063	Turnerbactin biosynthetic gene cluster (30% of genes show similarity)	-
Cluster 5	Terpene	23561	Carotenoid biosynthetic gene cluster (100% of genes show similarity)	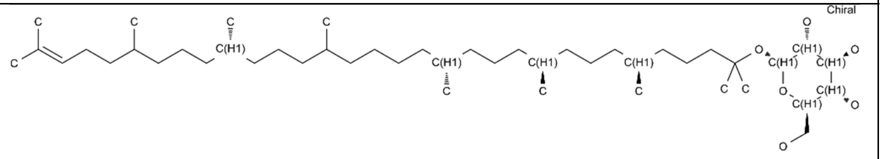
Cluster 6	Siderophore	12354	Desferrioxamine B biosynthetic gene cluster (40% of genes show similarity)	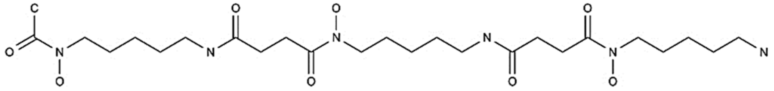

Supplementary Table S6. Continued.

Strain clusters	Type	Length (bp)	Most similar known clusters	Predicted core clusters
**3**				
Cluster 1	Hserlactone-Aryl polyene	69840	APE Ecbiosynthetic gene cluster (78% of genes show similarity)	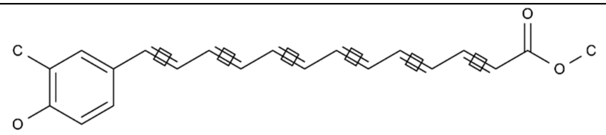
Cluster 2	Thiopeptide	26254	O-antigen biosynthetic gene cluster (14% of genes show similarity)	-
Cluster 3	Hserlactone	20638	-	-
Cluster 4	Nrps	52063	Turnerbactin biosynthetic gene cluster (30% of genes show similarity)	-
Cluster 5	Siderophore	12354	Desferrioxamine B biosynthetic gene cluster (60% of genes show similarity)	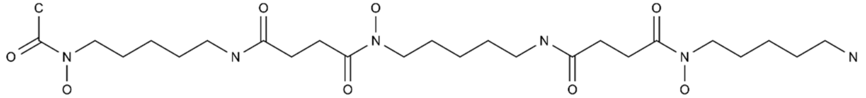
Cluster 6	Terpene	23561	Carotenoid biosynthetic gene cluster (83% of genes show similarity)	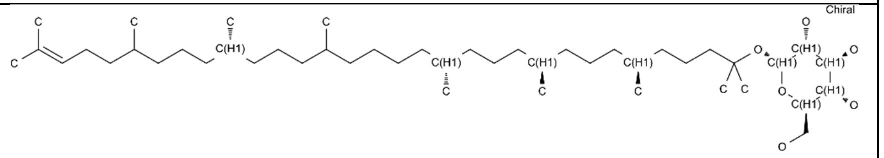

Supplementary Table S6. Continued.

Strain clusters	Type	Length (bp)	Most similar known clusters	Predicted core clusters
**4188**				
Cluster 1	Siderophore	12354	Desferrioxamine B biosynthetic gene cluster (60% of genes show similarity)	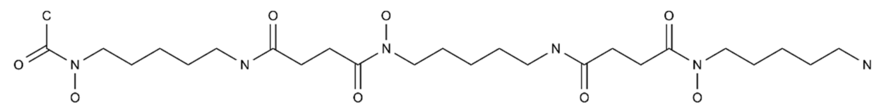
Cluster 2	Terpene	23561	Carotenoid biosynthetic gene cluster (100% of genes show similarity)	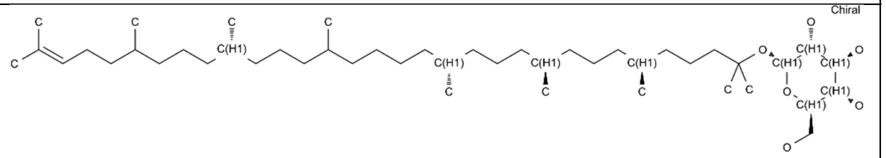
Cluster 3	Thiopeptide	26256	-	-
Cluster 4	Hserlactone	20611	-	-
Cluster 5	Aryl polyene	43586	APE Ecbiosynthetic gene cluster (84% of genes show similarity)	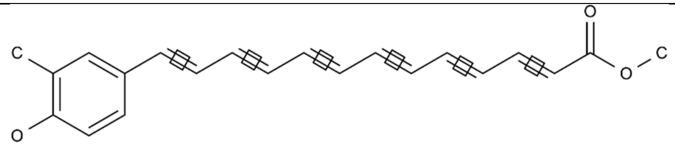
Cluster 6	Hserlactone	20638	-	-
Cluster 7	Nrps	52063	Turnerbactin biosynthetic gene cluster (30% of genes show similarity)	-

Supplementary Table S6. Continued.

Strain clusters	Type	Length (bp)	Most similar known clusters	Predicted core clusters
**C410P1**				
Cluster 1	Nrps	52062	Turnerbactin biosynthetic gene cluster (30% of genes show similarity)	-
Cluster 2	Hserlactone	20638	-	-
Cluster 3	Thiopeptide	26257	-	-
Cluster 4	Hserlactone-Aryl polyene	70891	APE Ecbiosynthetic gene cluster (78% of genes show similarity)	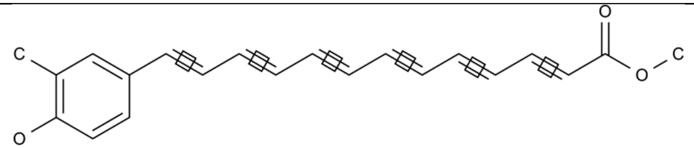

Supplementary Table S6. Continued.

Strain clusters	Type	Length (bp)	Most similar known clusters	Predicted core clusters
**190**				
Cluster 1	Nrps	52063	Turnerbactin biosynthetic gene cluster (30% of genes show similarity)	-
Cluster 2	Hserlactone	20638	-	-
Cluster 3	Thiopeptide	26256	-	-
Cluster 4	Hserlactone	20611	-	-
Cluster 5	Aryl polyene	43586	APE Ecbiosynthetic gene cluster (78% of genes show similarity)	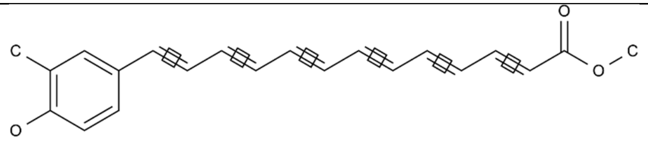
Cluster 6	Siderophore	12354	Desferrioxamine B biosynthetic gene cluster (40% of genes show similarity)	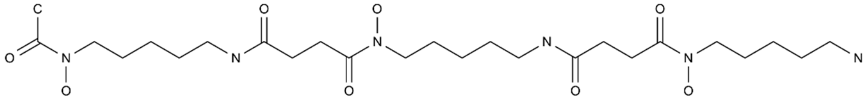
Cluster 7	Terpene	23561	Carotenoid biosynthetic gene cluster (100% of genes show similarity)	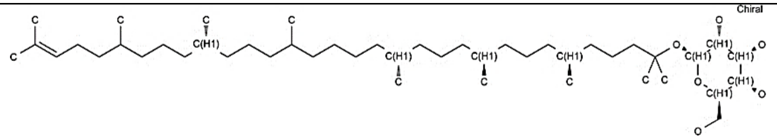
Cluster 8	Phenazine	20476	Pyocyanine biosynthetic gene cluster (28% of genes show similarity)	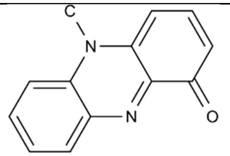

Supplementary Table S6. Continued.

Strain clusters	Type	Length (bp)	Most similar known clusters	Predicted core clusters
**4**				
Cluster 1	Nrps	52063	Turnerbactin biosynthetic gene cluster (30% of genes show similarity)	-
Cluster 2	Hserlactone	20638	-	-
Cluster 3	Thiopeptide	26256	-	-
Cluster 4	Hserlactone-Aryl polyene	69838	APE Ecbiosyntheti cgene cluster (78% of genes show similarity)	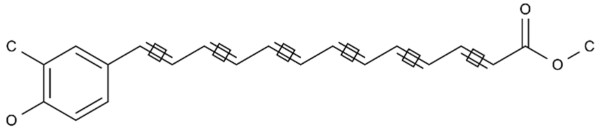
Cluster 5	Siderophore	12354	Desferrioxamine B biosynthetic gene cluster (60% of genes show similarity)	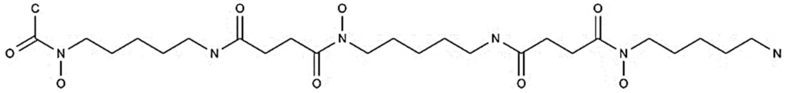
Cluster 6	Terpene	23561	Carotenoid biosynthetic gene cluster (83% of genes show similarity)	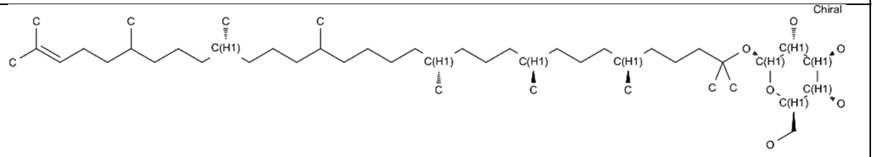
Cluster 7	Other	41736	-	-

Supplementary Table S6. Continued.

Strain clusters	Type	Length (bp)	Most similar known clusters	Predicted core clusters
**Eh318**				
Cluster 1	Thiopeptide	26255	-	-
Cluster 2	Hserlactone-Aryl polyene	69839	APE Ecbiosynthetic gene cluster (78% of genes show similarity)	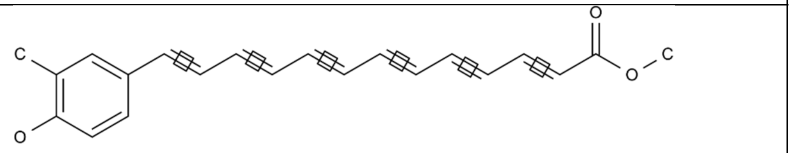
Cluster 3	Terpene	23561	Carotenoid biosynthetic gene cluster (100% of genes show similarity)	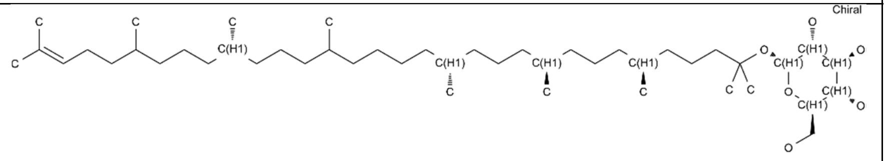
Cluster 4	Siderophore	12354	Desferrioxamine B biosynthetic gene cluster (60% of genes show similarity)	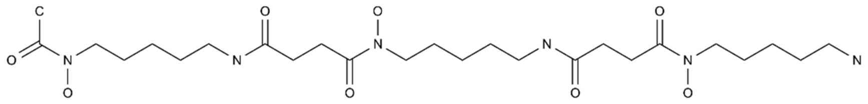
Cluster 5	Nrps	52063	Turnerbactin biosynthetic gene cluster (30% of genes show similarity)	-
Cluster 6	Hserlactone	20638	-	-

Supplementary Table S6. Continued.

Strain clusters	Type	Length (bp)	Most similar known clusters	Predicted core clusters
**DAPP-PG734**				
Cluster 1	Siderophore	8977	Desferrioxamine B biosynthetic gene cluster (60% of genes show similarity)	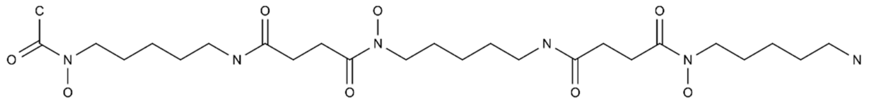
Cluster 2	Nrps	31380	Turnerbactin biosynthetic gene cluster (30% of genes show similarity)	-
Cluster 3	Terpene	23561	Carotenoid biosynthetic gene cluster (100% of genes show similarity)	
Cluster 4	Hserlactone	20611	-	-
Cluster 5	Aryl polyene	26442	APE Ecbiosynthetic gene cluster (26% of genes show similarity)	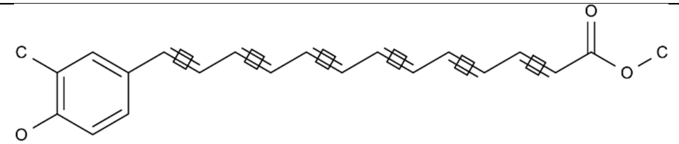
Cluster 6	Hserlactone	18529	-	-
Cluster 7	Thiopeptide	26256	O-antigen biosynthetic gene cluster (14% of genes show similarity)	-
Cluster 8	Other	15304	-	-

Supplementary Table S6. Continued.

Strain clusters	Type	Length (bp)	Most similar known clusters	Predicted core clusters
**FDAARGOS_160**				
Cluster 1	Nrps	52065	Turnerbactin biosynthetic gene cluster (30% of genes show similarity)	-
Cluster 2	Thiopeptide	26254	-	-
Cluster 3	Hserlactone	20611	-	-
Cluster 4	Aryl polyene	44486	APE Ecbiosynthetic gene cluster (89% of genes show similarity)	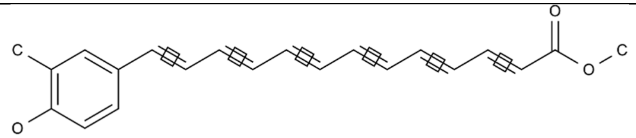

Supplementary Table S6. Continued.

Strain clusters	Type	Length (bp)	Most similar known clusters	Predicted core clusters
**C9-1**				
Cluster 1	Thiopeptide	26254	-	-
Cluster 2	Hserlactone-Aryl polyene	69818	APE Ecbiosynthetic gene cluster (89% of genes show similarity)	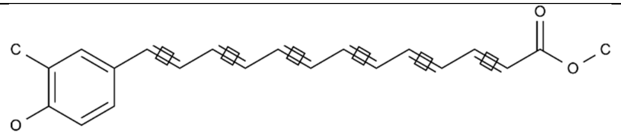
Cluster 3	Nrps	52055	Turnerbactin biosynthetic gene cluster (30% of genes show similarity)	-

Supplementary Table S6. Continued.

Strain clusters	Type	Length (bp)	Most similar known clusters	Predicted core clusters
**MP2**				
Cluster 1	Thiopeptide	26256	-	-
Cluster 2	Hserlactone-Aryl polyene	69852	APE Ecbiosynthetic gene cluster (78% of genes show similarity)	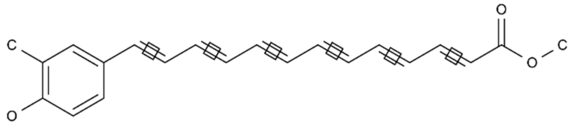
Cluster 3	Nrps	52059	Turnerbactin biosynthetic gene cluster (30% of genes show similarity)	-
Cluster 4	Hserlactone	14737	-	-
Cluster 5	Terpene	23561	Carotenoid biosynthetic gene cluster (100% of genes show similarity)	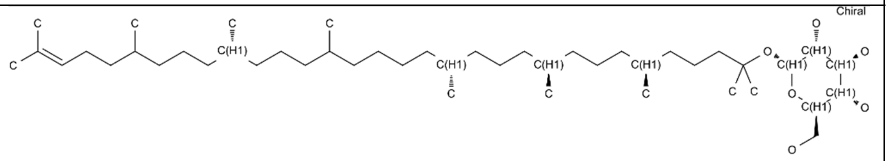
Cluster 6	Siderophore	12354	Desferrioxamine B biosynthetic gene cluster (40% of genes show similarity)	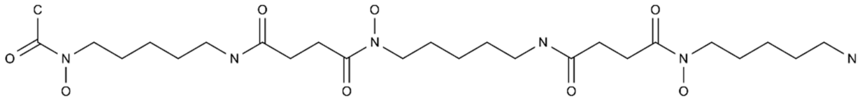

Supplementary Table S6. Continued.

Strain clusters	Type	Length (bp)	Most similar known clusters	Predicted core clusters
**LMAE-2**				
Cluster 1	Hserlactone	19435	-	-
Cluster 2	Nrps	52036	Turnerbactin biosynthetic gene cluster (30% of genes show similarity)	-
Cluster 3	Hserlactone-Aryl polyene	69856	APE Ecbiosynthetic gene cluster (84% of genes show similarity)	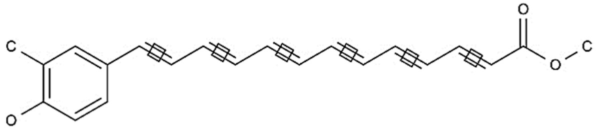
Cluster 4	Thiopeptide	26266	O-antigen biosynthetic gene cluster (14% of genes show similarity)	-
Cluster 5	Terpene	23561	Carotenoid biosynthetic gene cluster (100% of genes show similarity)	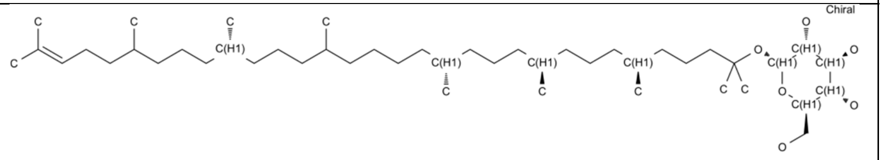
Cluster 6	Siderophore	12354	Desferrioxamine B biosynthetic gene cluster (60% of genes show similarity)	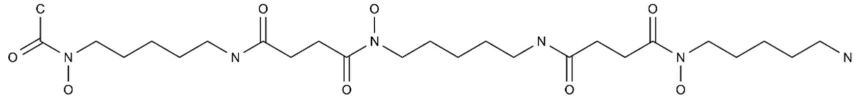

Supplementary Table S6. Continued.

Strain clusters	Type	Length (bp)	Most similar known clusters	Predicted core clusters
**P10c**				
Cluster 1	Thiopeptide	26256	-	-
Cluster 2	Hserlactone-Aryl polyene	69840	APE Ecbiosynthetic gene cluster (78% of genes show similarity)	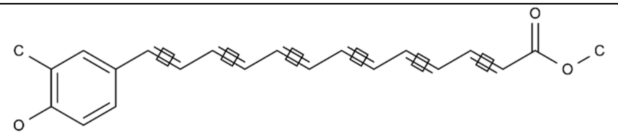
Cluster 3	Nrps	52063	Turnerbactin biosynthetic gene cluster (30% of genes show similarity)	-
Cluster 4	Hserlactone	20638	-	-
Cluster 5	Terpene	23561	Carotenoid biosynthetic gene cluster (100% of genes show similarity)	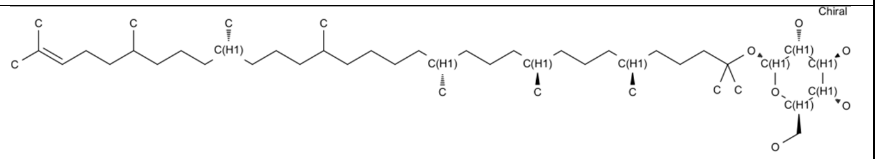
Cluster 6	Siderophore	12354	Desferrioxamine B biosynthetic gene cluster (40% of genes show similarity)	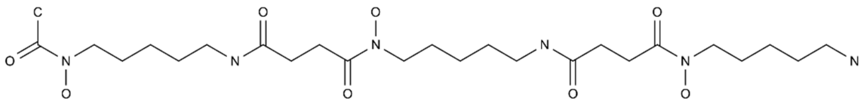

Supplementary Table S6. Continued.

Strain clusters	Type	Length (bp)	Most similar known clusters	Predicted core clusters
**RIT273**				
Cluster 1	Thiopeptide	26256	-	-
Cluster 2	Hserlactone	20611	-	-
Cluster 3	Aryl polyene	43586	APE Ecbiosynthetic gene cluster (73% of genes show similarity)	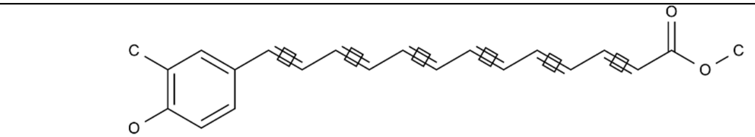
Cluster 4	Terpene	23561	Carotenoid biosynthetic gene cluster (100% of genes show similarity)	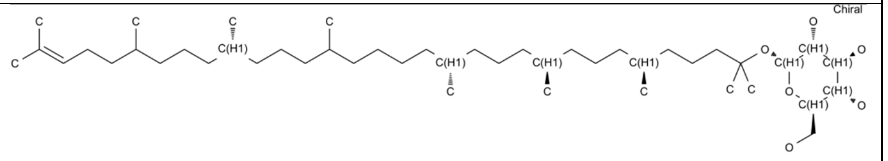
Cluster 5	Siderophore	12354	Desferrioxamine B biosynthetic gene cluster (40% of genes show similarity)	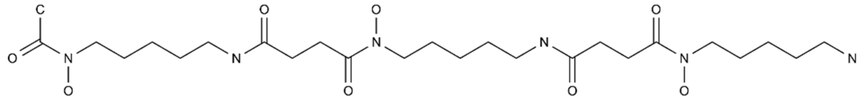
Cluster 6	Terpene	23561	Carotenoid biosynthetic gene cluster (100% of genes show similarity)	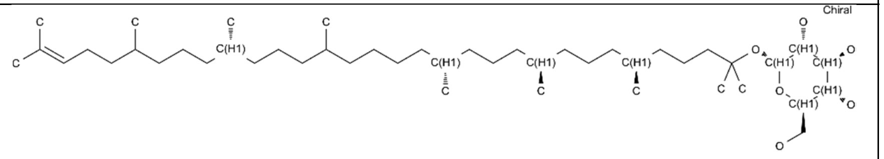
Cluster 7	Hserlactone	20638	-	-
Cluster 8	Nrps	52063	Turnerbactin biosynthetic gene cluster (30% of genes show similarity)	-

Supplementary Table S6. Continued.

Strain clusters	Type	Length (bp)	Most similar known clusters	Predicted core clusters
**MP7**				
Cluster 1	Nrps	52063	Turnerbactin biosynthetic gene cluster (38% of genes show similarity)	-
Cluster 2	Thiopeptide	26254	-	-
Cluster 3	Hserlactone	20611	-	-
Cluster 4	Hserlactone	20638	-	-
Cluster 5	Terpene	23561	Carotenoid biosynthetic gene cluster (100% of genes show similarity)	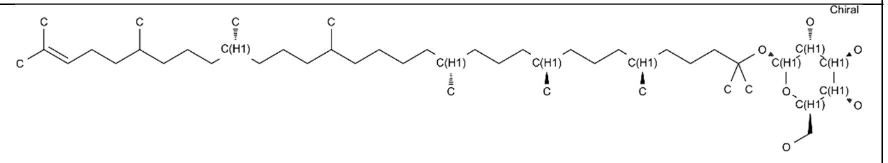
Cluster 6	Siderophore	8309	Desferrioxamine B biosynthetic gene cluster (60% of genes show similarity)	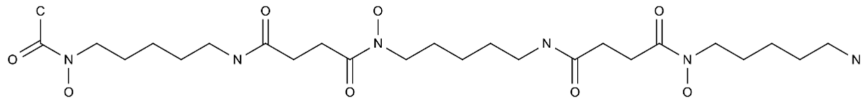

Supplementary Table S6. Continued.

Strain clusters	Type	Length (bp)	Most similar known clusters	Predicted core clusters
**Tx10**				
Cluster 1	Microcin	1405	-	-
Cluster 2	Siderophore	12354	Desferrioxamine B biosynthetic gene cluster (40% of genes show similarity)	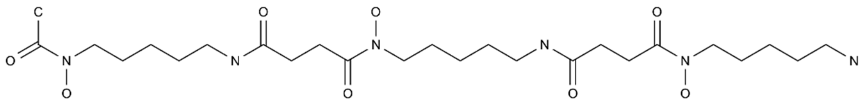
Cluster 3	Terpene	23561	Carotenoid biosynthetic gene cluster (100% of genes show similarity)	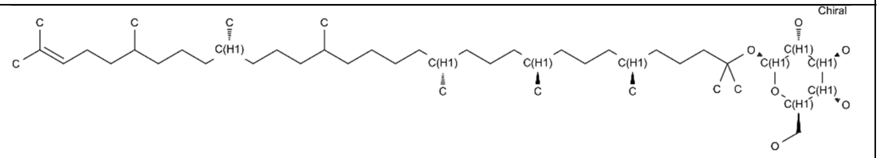
Cluster 4	Nrps	52063	Turnerbactin biosynthetic gene cluster (30% of genes show similarity)	-
Cluster 5	Hserlactone	20638	-	-
Cluster 6	Hserlactone-Aryl polyene	69839	APE Ecbiosynthetic gene cluster (78% of genes show similarity)	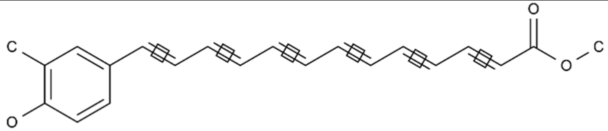
Cluster 7	Thiopeptide	26256	O-antigen biosynthetic gene cluster (14% of genes show similarity)	-

Supplementary Table S6. Continued.

Strain clusters	Type	Length (bp)	Most similar known clusters	Predicted core clusters
**PaVv1**				
Cluster 1	Siderophore	12354	Desferrioxamine B biosynthetic gene cluster (40% of genes show similarity)	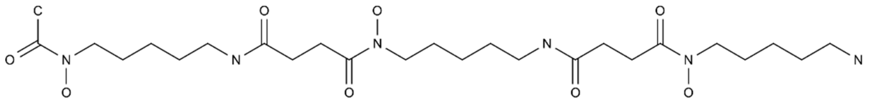
Cluster 2	Terpene	23561	Carotenoid biosynthetic gene cluster (100% of genes show similarity)	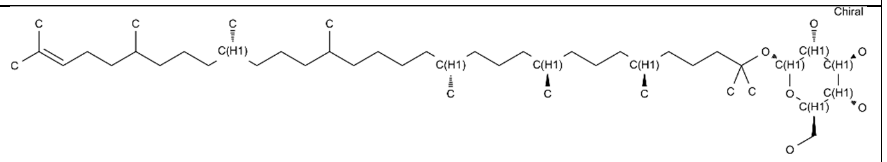
Cluster 3	Nrps	52063	Turnerbactin biosynthetic gene cluster (30% of genes show similarity)	-
Cluster 4	Hserlactone	20638	-	-
Cluster 5	Hserlactone-Aryl polyene	69849	APE Ecbiosynthetic gene cluster (78% of genes show similarity)	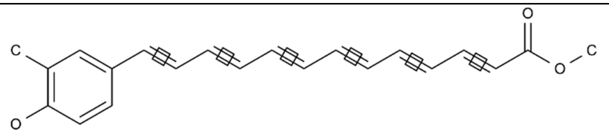
Cluster 6	Thiopeptide	26256	O-antigen biosynthetic gene cluster (14% of genes show similarity)	-

Supplementary Table S6. Continued.

Strain clusters	Type	Length (bp)	Most similar known clusters	Predicted core clusters
**848_PVAG**				
Cluster 1	Terpene	23563	Carotenoid biosynthetic gene cluster (100% of genes show similarity)	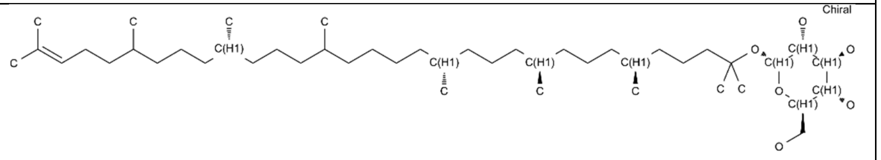
Cluster 2	Hserlactone	14599	-	-
Cluster 3	Siderophore	7304	-	-
Cluster 4	Thiopeptide	18090	-	-
Cluster 5	Siderophore	5203	Aerobactin biosynthetic gene cluster (22% of genes show similarity)	-
Cluster 6	Nrps	20621	Amychelin biosynthetic gene cluster (18% of genes show similarity)	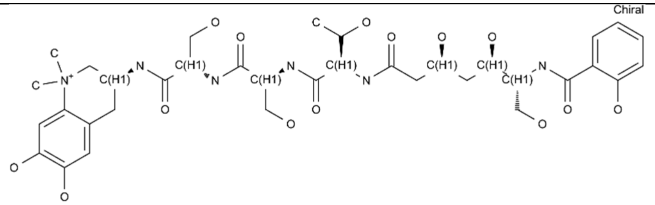
Cluster 7	Aryl polyene	42418	APE Ecbiosynthetic gene cluster (73% of genes show similarity)	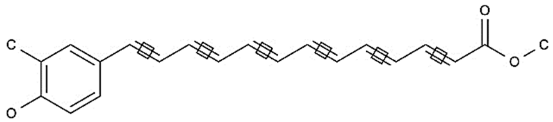

Supplementary Table S6. Continued.

Strain clusters	Type	Length (bp)	Most similar known clusters	Predicted core clusters
**ZBG6**				
Cluster 1	Siderophore	12354	Desferrioxamine B biosynthetic gene cluster (60% of genes show similarity)	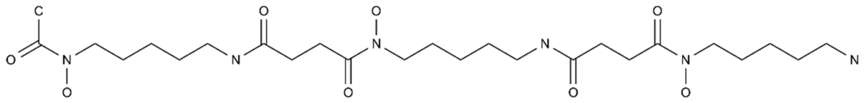
Cluster 2	Terpene	23561	Carotenoid biosynthetic gene cluster (83% of genes show similarity)	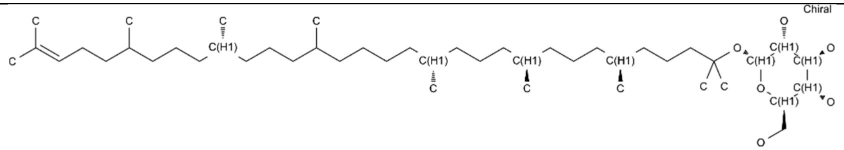
Cluster 3	Thiopeptide	26256	-	-
Cluster 4	Hserlactone	20638	-	-
Cluster 5	Nrps	52063	Turnerbactin biosynthetic gene cluster (30% of genes show similarity)	-
Cluster 6	Hserlactone-Aryl polyene	50404	APE Ecbiosynthetic gene cluster (78% of genes show similarity)	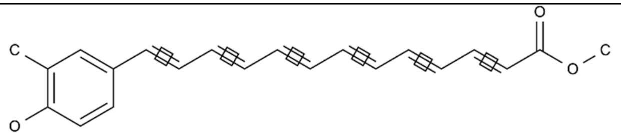

Supplementary Table S6. Continued.

Strain clusters	Type	Length (bp)	Most similar known clusters	Predictedc ore clusters
**NFPP29**				
Cluster 1	Thiopeptide	26253	-	-
Cluster 2	Hserlactone-Aryl polyene	68767	APE Ecbiosynthetic gene cluster (78% of genes show similarity)	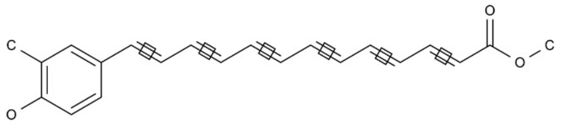

Supplementary Table S6. Continued.

Strain clusters	Type	Length (bp)	Most similar known clusters	Predicted core clusters
**P5**				
Cluster 1	Nrps	52063	Turnerbactin biosynthetic gene cluster (30% of genes show similarity)	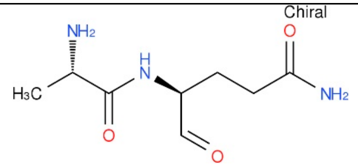
Cluster 2	Hserlactone	20638	-	-
Cluster 3	Hserlactone-Aryl polyene	69810	APE Ecbiosynthetic gene cluster (84% of genes show similarity)	-
Cluster 4	Siderophore	7860	Desferrioxamine B biosynthetic gene cluster (60% of genes show similarity)	-
Cluster 5	Terpene	23561	Carotenoid biosynthetic gene cluster (83% of genes show similarity)	-

Supplementary Figure S1. (**A**) Genome-to-Genome Distance Calculation (GGDC) and (**B**) Average Nucleotide Identity (ANI) values between studied strains.

(A)

GGDC	299R	NFPP29	MP7	FDAARGOS_160	C9-1	848_PVAG	824-1	3	4	Eh318	Tx10	NBRC 102470	190	IG1	P10c	ZBG6	MP2	RIT273	PaVv1	LMAE-2	4188	C410P1	DAPP-PG734	Pa	P5
299R	98.3	96.98	2.75	3.86	4.21	0	3.77	3.6	3.83	3.81	3.69	3.76	3.83	3.62	3.64	3.67	3.8	3.75	3.74	3.55	3.51	5.28	3.66	3.52	3.9
NFPP29	96.98	98.3	3.64	3.78	4.16	0	4.78	4.69	4.79	4.84	4.64	4.73	4.82	4.71	4.66	4.66	4.62	4.82	4.78	4.49	4.63	5.25	4.56	4.57	4.43
MP7	2.75	3.64	98.3	77.53	84.89	0	5.47	5.57	5.65	5.5	5.4	5.41	5.57	5.01	5.33	5.33	5.68	5.23	5.27	5.25	5.03	7.63	5.27	4.97	6.22
FDAARGOS_160	3.86	3.78	77.53	98.3	82.97	0	7.09	7.23	7.13	7.21	6.96	6.82	7.36	6.6	6.66	6.97	7.45	6.98	6.68	6.68	6.54	6.96	6.65	6.53	6.35
C9-1	4.21	4.16	84.89	82.97	98.3	0	7.97	8.16	8.11	8.02	7.8	7.69	7.95	7.36	7.62	7.65	8.43	7.82	7.47	7.37	7.15	7.7	7.42	7.26	7.05
848_PVAG	0	0	0	0	0	98.3	0	0	0	0	0	0	0	0	0	0	0	0	0	0	0	0	0	0	0
824-1	3.77	4.78	5.47	7.09	7.97	0	98.3	95.17	94.88	95.06	95.11	94.93	94.74	95.2	94.96	94.86	95.14	94.87	94.7	89.36	89.62	91.43	88.88	89.54	87.75
3	3.6	4.69	5.57	7.23	8.16	0	95.17	98.3	95.42	95.53	95.5	95.47	95.39	95.56	95.46	95.44	95.52	95.37	95.25	90.11	89.61	91.6	90.46	89.79	88.2
4	3.83	4.79	5.65	7.13	8.11	0	94.88	95.42	98.3	95.4	95.49	98.02	95.24	95.23	95.5	95.4	95.53	95.52	95.25	90.07	89.83	91.71	90.06	89.74	88.68
Eh318	3.81	4.84	5.5	7.21	8.02	0	95.06	95.53	95.4	98.3	95.42	95.39	95.73	95.36	95.42	95.38	95.55	95.39	95.35	90.04	89.59	91.58	90.67	89.86	88.66
Tx10	3.69	4.64	5.4	6.96	7.8	0	95.11	95.5	95.49	95.42	98.3	95.44	95.39	95.47	95.52	95.4	95.52	95.44	95.36	90.12	89.8	91.8	90.78	89.88	88.68
NBRC 102470	3.76	4.73	5.41	6.82	7.69	0	94.93	95.47	98.02	95.39	95.44	98.3	95.23	95.35	95.33	95.29	95.49	95.27	95.15	90.17	89.9	91.6	90.83	89.95	88.71
190	3.83	4.82	5.57	7.36	7.95	0	94.74	95.39	95.24	95.73	95.39	95.23	98.3	95.18	95.35	95.21	95.28	95.2	95.17	89.75	89.47	91.59	89.9	89.82	88.43
IG1	3.62	4.71	5.01	6.6	7.36	0	95.2	95.56	95.23	95.36	95.47	95.35	95.18	98.3	95.45	95.4	97.14	95.31	95.11	89.76	90.08	91.46	90.74	90.08	88.29
P10c	3.64	4.66	5.33	6.66	7.62	0	94.96	95.46	95.5	95.42	95.52	95.33	95.35	95.45	98.3	95.41	95.5	95.34	95.3	89.83	89.67	91.42	90.62	96.33	88.14
ZBG6	3.67	4.66	5.33	6.97	7.65	0	94.86	95.44	95.4	95.38	95.4	95.29	95.21	95.4	95.41	98.3	95.89	95.65	95.62	90.19	89.91	91.55	90.47	89.59	88.95
MP2	3.8	4.62	5.68	7.45	8.43	0	95.14	95.52	95.53	95.55	95.52	95.49	95.28	97.14	95.5	95.89	98.3	95.76	95.57	90.28	89.86	91.85	90.93	89.91	88.72
RIT273	3.75	4.82	5.23	6.98	7.82	0	94.87	95.37	95.52	95.39	95.44	95.27	95.2	95.31	95.34	95.65	95.76	98.3	95.57	90.06	89.42	91.64	90.81	89.81	88.6
PaVv1	3.74	4.78	5.27	6.68	7.47	0	94.7	95.25	95.25	95.35	95.36	95.15	95.17	95.11	95.3	95.62	95.57	95.57	98.3	89.95	89.45	91.42	90.24	89.77	88.3
LMAE-2	3.55	4.49	5.25	6.68	7.37	0	89.36	90.11	90.07	90.04	90.12	90.17	89.75	89.76	89.83	90.19	90.28	90.06	89.95	98.3	95.08	95.6	95.56	95.28	88.94
4188	3.51	4.63	5.03	6.54	7.15	0	89.62	89.61	89.83	89.59	89.8	89.9	89.47	90.08	89.67	89.91	89.86	89.42	89.45	95.08	98.3	95.47	94.76	95.25	88.77
C410P1	5.28	5.25	7.63	6.96	7.7	0	91.43	91.6	91.71	91.58	91.8	91.6	91.59	91.46	91.42	91.55	91.85	91.64	91.42	95.6	95.47	98.3	95.68	96.36	89.58
DAPP-PG734	3.66	4.56	5.27	6.65	7.42	0	88.88	90.46	90.06	90.67	90.78	90.83	89.9	90.74	90.62	90.47	90.93	90.81	90.24	95.56	94.76	95.68	98.3	95.64	88.68
Pa	3.52	4.57	4.97	6.53	7.26	0	89.54	89.79	89.74	89.86	89.88	89.95	89.82	90.08	96.33	89.59	89.91	89.81	89.77	95.28	95.25	96.36	95.64	98.3	88.92
P5	3.9	4.43	6.22	6.35	7.05	0	87.75	88.2	88.68	88.66	88.68	88.71	88.43	88.29	88.14	88.95	88.72	88.6	88.3	88.94	88.77	89.58	88.68	88.92	98.3

(B)

ANI	299R	NFPP29	MP7	FDAARGOS_160	C9-1	848_PVAG	824-1	3	4	Eh318	Tx10	NBRC 102470	190	IG1	P10c	ZBG6	MP2	RIT273	PaVv1	LMAE-2	4188	C410P1	DAPP-PG734	Pa	P5
299R	100	99.25	90.1	90.52	90.81	79.52	90.65	90.86	90.97	90.92	90.96	90.53	90.93	90.95	90.94	90.47	90.95	90.93	90.59	90.9	90.42	91.04	90.89	90.52	90.65
NFPP29	99.25	100	90.63	90.71	90.72	79.94	90.98	90.89	90.99	90.95	90.9	90.96	90.96	91.04	90.93	90.92	91.01	91.01	91.02	90.95	90.86	91.18	90.9	91	90.94
MP7	90.1	90.63	100	96.38	97.02	80.13	91	91.06	91.1	90.94	91.06	91.06	91.17	90.99	91.01	90.95	91.02	90.9	90.99	90.92	90.92	91.65	91.03	90.94	91.28
FDAARGOS_160	90.52	90.71	96.38	100	96.73	80.43	91.56	91.41	91.43	91.46	91.36	91.63	91.49	91.36	91.44	91.39	91.52	91.44	91.34	91.35	91.35	91.31	91.31	91.47	91.28
C9-1	90.81	90.72	97.02	96.73	100	80.38	91.66	91.61	91.71	91.73	91.66	91.61	91.57	91.61	91.61	91.57	91.55	91.51	91.63	91.44	91.37	91.46	91.51	91.49	91.51
848_PVAG	79.52	79.94	80.13	80.43	80.38	100	80.1	80.19	80.05	80.28	80.23	80.05	80.09	80.12	80	80	80.15	80.07	80.12	80.52	80.11	80.31	80.12	80.05	80.08
824-1	90.65	90.98	91	91.56	91.66	80.1	100	98.51	98.51	98.51	98.56	98.52	98.43	98.55	98.52	98.56	98.58	98.51	98.35	97.48	97.48	97.64	97.21	97.49	97.22
3	90.86	90.89	91.06	91.41	91.61	80.19	98.51	100	98.78	98.75	98.77	98.7	98.79	98.79	98.76	98.69	98.79	98.78	98.59	97.71	97.36	97.72	97.73	97.55	97.32
4	90.97	90.99	91.1	91.43	91.71	80.05	98.51	98.78	100	98.79	98.81	98.67	98.79	98.73	98.82	98.66	98.83	98.81	98.6	97.74	97.57	97.83	97.78	97.61	97.44
Eh318	90.92	90.95	90.94	91.46	91.73	80.28	98.51	98.75	98.79	100	98.81	98.67	98.68	98.74	98.76	98.67	98.73	98.71	98.59	97.76	97.58	97.86	97.85	97.54	97.46
Tx10	90.96	90.9	91.06	91.36	91.66	80.23	98.56	98.77	98.81	98.81	100	98.71	98.75	98.78	98.8	98.72	98.8	98.75	98.67	97.77	97.56	97.81	97.85	97.55	97.47
NBRC 102470	90.53	90.96	91.06	91.63	91.61	80.05	98.52	98.7	98.67	98.67	98.71	100	98.64	98.66	98.57	98.64	98.69	98.63	98.58	97.57	97.52	97.85	97.61	97.57	97.43
190	90.93	90.96	91.17	91.49	91.57	80.09	98.43	98.79	98.79	98.68	98.75	98.64	100	98.73	98.76	98.61	98.75	98.76	98.62	97.74	97.42	97.82	97.69	97.53	97.39
IG1	90.95	91.04	90.99	91.36	91.61	80.12	98.55	98.79	98.73	98.74	98.78	98.66	98.73	100	98.79	98.67	98.8	98.72	98.57	97.77	97.49	97.85	97.81	97.58	97.36
P10c	90.94	90.93	91.01	91.44	91.61	80	98.52	98.76	98.82	98.76	98.8	98.57	98.76	98.79	100	98.71	98.82	98.79	98.58	97.73	97.54	97.78	97.76	97.52	97.29
ZBG6	90.47	90.92	90.95	91.39	91.57	80	98.56	98.69	98.66	98.67	98.72	98.64	98.61	98.67	98.71	100	98.85	98.75	98.72	97.55	97.55	97.78	97.6	97.56	97.48
MP2	90.95	91.01	91.02	91.52	91.55	80.15	98.58	98.79	98.83	98.73	98.8	98.69	98.75	98.8	98.82	98.85	100	98.86	98.74	97.8	97.51	97.88	97.9	97.58	97.4
RIT273	90.93	91.01	90.9	91.44	91.51	80.07	98.51	98.78	98.81	98.71	98.75	98.63	98.76	98.72	98.79	98.75	98.86	100	98.68	97.75	97.44	97.72	97.83	97.58	97.37
PaVv1	90.59	91.02	90.99	91.34	91.63	80.12	98.35	98.59	98.6	98.59	98.67	98.58	98.62	98.57	98.58	98.72	98.74	98.68	100	97.55	97.58	97.78	97.62	97.57	97.41
LMAE-2	90.9	90.95	90.92	91.35	91.44	80.52	97.48	97.71	97.74	97.76	97.77	97.57	97.74	97.77	97.73	97.55	97.8	97.75	97.55	100	98.55	98.71	98.77	98.6	97.46
4188	90.42	90.86	90.92	91.35	91.37	80.11	97.48	97.36	97.57	97.58	97.56	97.52	97.42	97.49	97.54	97.55	97.51	97.44	97.58	98.55	100	98.65	98.4	98.59	97.48
C410P1	91.04	91.18	91.65	91.31	91.46	80.31	97.64	97.72	97.83	97.86	97.81	97.85	97.82	97.85	97.78	97.78	97.88	97.72	97.78	98.71	98.65	100	98.7	98.98	97.54
DAPP-PG734	90.89	90.9	91.03	91.31	91.51	80.12	97.21	97.73	97.78	97.85	97.85	97.61	97.69	97.81	97.76	97.6	97.9	97.83	97.62	98.77	98.4	98.7	100	98.7	97.4
Pa	90.52	91	90.94	91.47	91.49	80.05	97.49	97.55	97.61	97.54	97.55	97.57	97.53	97.58	97.52	97.56	97.58	97.58	97.57	98.6	98.59	98.98	98.7	100	97.46
P5	90.65	90.94	91.28	91.28	91.51	80.08	97.22	97.32	97.44	97.46	97.47	97.43	97.39	97.36	97.29	97.48	97.4	97.37	97.41	97.46	97.48	97.54	97.4	97.46	100

Supplementary Figure S2. Maximum Likelihood phylogenetic tree (16SrRNA gene) of *Pantoea agglomerans* strain Pa and its closest *P. vagans and P. agglomerans* relatives. *Dickeyazea* (accession: AF520711) was used as outgroup.

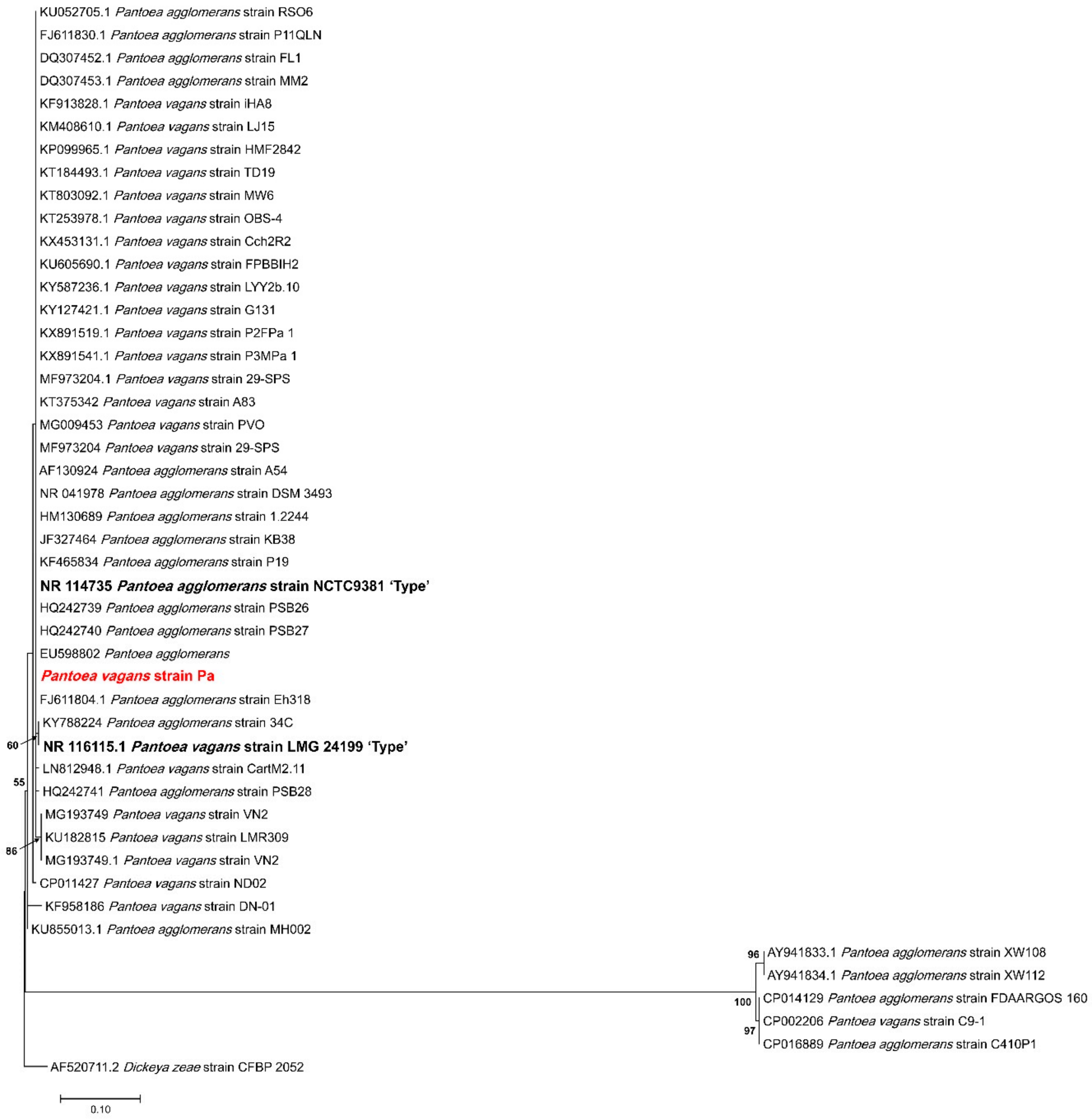

Supplementary Figure S3. Control and *P. agglomerans* isolate Pa inoculated *Triticum durum* L. cv Waha plants grown in presence of 100 mM NaCl.

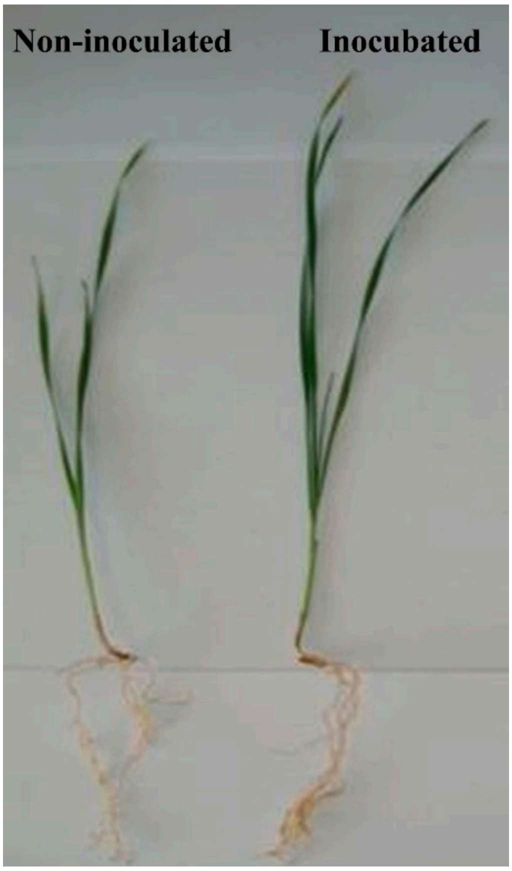

Supplementary Figure S4. Control and *P. agglomerans* isolate Pa inoculated *Triticum durum* L. cv Waha plants grown in soil in presence of 100 mM NaCl.

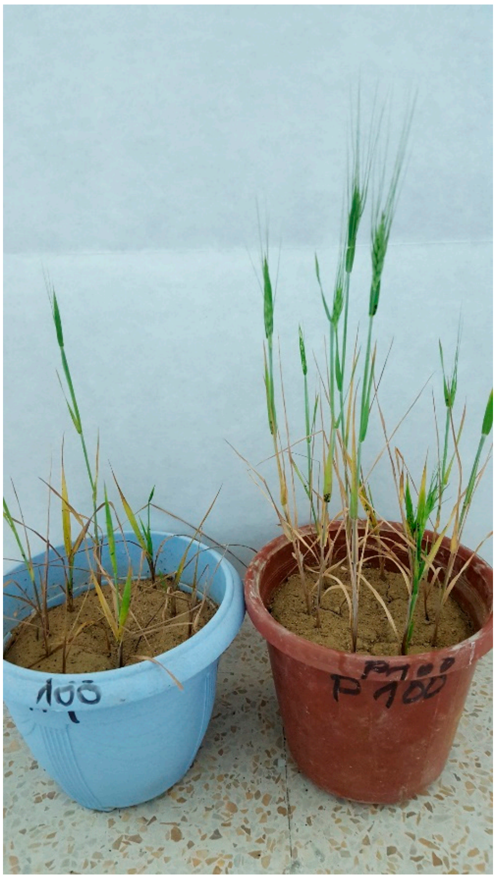

Supplementary Figure S5. Comparison between LC-HRESIMS profiles of six different media (M1-M6) and their blanks.

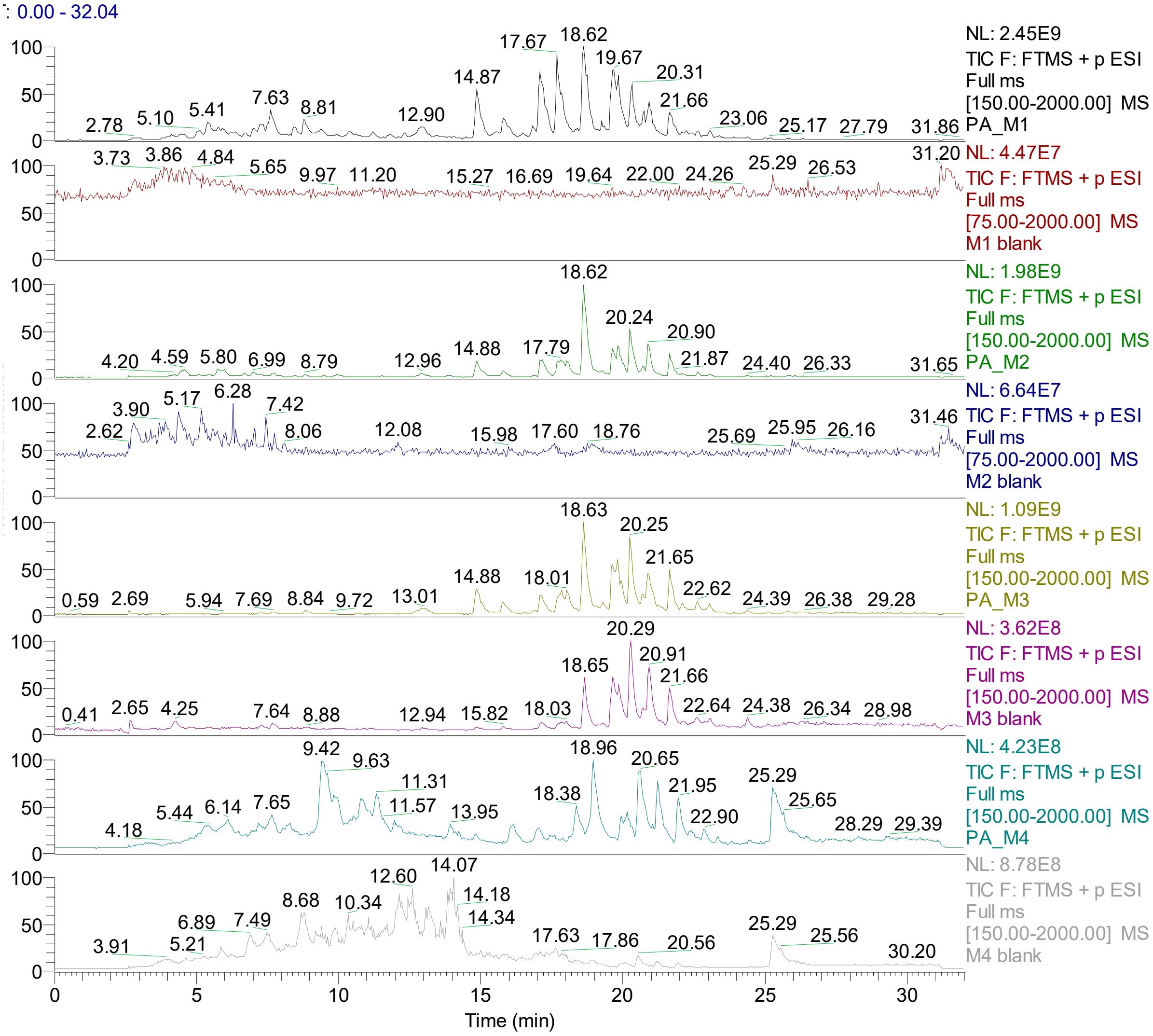

References

1. Bruto, M.; Prigent-Combaret, C.; Muller, D.; Moënne-Loccoz, Y. Analysis of genes contributing to plant-beneficial functions in plant growth-promoting rhizobacteria and related proteobacteria. *Sci. Rep*. **2014**, 4, 6261. doi:10.1038/srep06261.
2. Miller, S.H.; Browne, P.; Prigent-Combaret, C.; Combes-Meynet, E.; Morrissey, J.P.; O' Gara, F. Biochemical and genomic comparison of inorganic phosphate solubilization in *Pseudomonas* species. *Environ. Microbiol. Rep*. **2010**, *2*, 403-411. doi:10.1111/j.1758-2229.2009.00105.x.
3. Liu, S.; Lee, L.; Tai, C.; Hung, C.; Chang, Y.; Wolfram, J., et al. Cloning of an *Erwinia herbicola* gene necessary for gluconic acid production and enhanced mineral phosphate solubilization in *Escherichia coli* HB101: nucleotide sequence and probable involvement in biosynthesis of the coenzyme pyrroloquinoline quinone. *J. Bacteriol*. **1992**, *174*, 5814–5819. doi:10.1128/jb.174.18.5814-5819.1992.
4. Kim, J.H.; Ji, C.J.; Ju, S.Y.; Yang, Y.M.; Ryu. S.H.; Kwon, Y.; Won, Y.B.; Lee, Y.E.; Youn, H.;Lee, J.W. *Bacillus licheniformis* contains two more PerR-like proteins in addition to PerR, Fur, and Zur orthologues. *PLoS ONE*.**2003**, 11, e0155539. doi:10.1371/journal.pone.0155539.
5. Shao, J.H.; Li, S.Q.; Zhang, N.; Cui, X.S.; Zhou, X.; Zhang, G.S.; Shen, Q.R.; Zhang, R.F. Analysis and cloning of the synthetic pathway of the phytohormone indole-3-acetic acid in the plant-beneficial *Bacillus amyloliquefaciens* SQR9. *Microb. Cell Fact*. **2015**, 14, 130. doi:10.1186/s12934-015-0323-4.
6. Niazi, A.; Manzoor, S.; Asari, S.; Bejai, S.; Meijer, J.; Bongcam-Rudloff, E. Genome analysis of *Bacillus amyloliquefaciens* subsp. *plantarum* UCMB5113: A rhizobacterium that improves plant growth and stress management. *PLoS ONE*. **2014**, 9, e104651. doi:10.1371/journal.pone.0104651.
7. Petersen, T.N.; Brunak, S.; von Heijne, G.; Nielsen, H. SignalP 4.0: Discriminating signal peptides from transmembrane regions. *Nature Methods*. **2011**, *8*, 785–786. doi:10.1038/nmeth.1701.
8. Mazzola, M.; Cook, R.J.; Thomashow, L.S.; Weller, D.M.; Pierson, L.S. Contribution of phenazine antibiotic biosynthesis to the ecological competence of fluorescent pseudomonads in soil habitats. *Appl. Environ. Microbiol*. **1992**, *58*, 2616-2624.
9. Boch, J.; Kempf, B.; Schmid, R.; Bremer, E. Synthesis of the osmoprotectant glycine betaine in *Bacillus subtilis*: characterization of the gbsAB genes. *J. Bacteriol*. **1996**, *178*, 5121-5129.
10. Gupta, A.; Gopal, M.; Thomas, G.V.; Manikandan, V.; Gajewski, J.; Thomas, G.; Seshagiri, S.; Schuster, S.C.; Rajesh, P.;Gupta, R. Whole genome sequencing and analysis of plant growth promoting bacteria isolated from the rhizosphere of plantation crops coconut, cocoa and arecanut. *PLoS ONE*. **2014**, 9, e104259. doi:10.1371/journal.pone.0104259.
11. Sandkvist, M. Type II Secretion and Pathogenesis. *Infect. Immun*. **2001**, *69(6)*, 3523–3535. doi:10.1128/IAI.69.6.3523-3535.2001.
12. Ghelardi, E.; Salvetti, S.; Ceragioli, M.; Gueye, S.A.; Celandroni, F.; Senesi, S. Contribution of surfactin and SwrA to flagellin expression, swimming, and surface motility in *Bacillus subtilis*. *Appl. Environ. Microbiol*.**2012**,*78*: 6540-6544. doi:10.1128/AEM.01341-12.
13. Shen, X.; Hu, H.; Peng, H.; Wang, W.; Zhang, X. Comparative genomic analysis of four representative plant growth-promoting rhizobacteria in *Pseudomonas*. *BMC Genomics*. **2013**, 14, 271. doi:10.1186/1471-2164-14-271.
14. Vlamakis, H.; Chai, Y.; Beauregard, P.; Losick, R.; Kolter, R. Sticking together: building a biofilm the Bacillus subtilis way. *Nat. Rev. Microbiol*. **2013**, *11*, 157-168. doi:10.1038/nrmicro2960.
15. Zimmer, W.; Aparicio, C.; Elmerich, C. Relationship between tryptophane biosynthesis and indole-3-acetic acid production in *Azospirillum*: Identification and sequencing of a trpGDC cluster. *Mol. Gen. Genet*. **1991**, *229*, 41-51.
16. Idris, E.E.S.; Iglesias, D.J.; Talon, M.; Borriss, R. Tryptophan-dependent production of indole-3-acetic acid (IAA) affects level of plant growth promotion by *Bacillus amyloliquefaciens* FZB42. *Mol. Plant Microbe Interact*. **2007**, *20*, 619-626. doi:10.1094/MPMI-20-6-0619.

17. Vacheron, J.; Desbrosses, G.; Bouffaud, M-L.; Touraine, B.; Moënne-Loccoz, Y.; Muller, D.; Legendre, L.; Wisniewski-Dyé, F.; Prigent-Combaret, C. Plant growth-promoting rhizobacteria and root system functioning. *Front. Plant Sci*. **2013**, 4, 356. doi:10.3389/fpls.2013.00356.
18. Somers, E.; Ptacek, D.; Gysegom, P.; Srinivasan, M.; Vanderleyden, J. *Azospirillum brasilense* produces the auxin-like phenylacetic acid by using the key enzyme for indole-3-acid biosynthesis. *Appl. Environ. Microbiol*. **2005**, *71*, 1803-1810. doi:10.1128/AEM.71.4.1803-1810.2005.
19. Glick, B.R.; Jacobson, C.B.; Schwarze, M.M.K.; Pasternak, J.J. 1-Aminocyclopropane-1-carboxylic acid deaminase mutants of the plant growth promoting rhizobacterium *Pseudomonas putida* GR12-2 do not stimulate canola root elongation. *Can. J. Microbiol*. **1994**, *40*, 911-915. doi:10.1139/m94-146.
20. Xie, S.S.; Wu, H.J.; Zang, H.Y.; Wu, L.M.; Zhu, Q.Q.; Gao, X.W. Plant growth promotion by spermidine-producing *Bacillus subtilis* OKB105. *Mol. Plant Microbe Interact*. **2014**, *27*, 655-663. doi:10.1094/MPMI-01-14-0010-R.
21. Ochsner, U.A.; Vasil, M.L.; Alsabbagh, E.; Parvatiyar, K.; Hassett, D.J. Role of the *Pseudomonas aeruginosa* oxyR-recG operon in oxidative stress defense and DNA repair: OxyR-dependent regulation of katB-ankB, ahpB, and ahpC-ahpF. *J.Bacteriol*. **2000**, *182*, 4533-4544.
22. Whistler, C.A.; Corbell, N.A.; Sarniguet, A.; Ream, W.; Loper, J.E. The two-component regulators GacS and GacA influence accumulation of the stationary-phase sigma factor sigmaS and the stress response in *Pseudomonas fluorescens* Pf-5. *J. Bacteriol*. **1998**, *180*, 6635-6641.
23. Loper, J.E.; Hassan, K.A.; Mavrodi, D.V.; Davis, E.W.; Lim, C.K.; Shaffer, B.T.; Elbourne, L.D.H.; Stockwell, V.O.; Hartney, S.L.; Breakwell, K.; Henkels, M.D.; Tetu, S.G.; Rangel, L.I.; Kidarsa, T.A.; Wilson, N.L.; van de Mortel, J.E.; Song, C.; Blumhagen, R.; Radune, D.; Hostetler, J.B.;Brinkac, L.M.; Durkin, A.S.; Kluepfel, D.A.; Wechter, W.P.; Anderson, A.J.; Kim, Y.C.; Pierson, L.S.; Pierson, E.A.; Lindow, S.E.; Kobayashi, D.Y.; Raaijmakers, J.M.; Weller, D.M.; Thomashow, L.S.; Allen, A.E.; Paulsen, I.T. Comparative genomics of plant-associated *Pseudomonas* spp.: insights into diversity and inheritance of traits involved in multitrophic interactions. *PLoS Genet*. **2012**, 8, e1002784. doi:10.1371/journal.pgen.1002784.
24. Sakaguchi, R.; Amano, H.; Shishido, K. (1988). Nucleotide sequence homology of the tetracycline-resistance determinant naturally maintained in *Bacillus subtilis* Marburg 168 chromosome and the tetracycline-resistance gene of *B. subtilis* plasmid pNS1981. *Biochim. Biophys. Acta*. **1988**, *950*, 441-444.
25. Someya, Y.; Yamaguchi, A.; Sawai, T. A novel glycylcycline, 9-(N,N-dimethylglycylamido)-6-demethyl-6-deoxytetracycline, is neither transported nor recognized by the transposon Tn10-encoded metal-tetracycline/H+ antiporter. *Antimicrob. Agents Chemother*. **1995**, 39: 247–249.
26. Jacob, J.; Evers, S.; Bischoff, K.; Carlier, C.; Courvalin, P. Characterization of the *sat4* gene encoding a streptothricin acetyltransferase in *Campylobacter coli* BE/G4. *FEMS Microbiol. Lett*.**1994**,*120*: 13-17.
27. Fu, Z.; Liu, Y.; Chen, C.; Guo, Y.; Ma, Y.; Yang, Y.; Fupin, H.; Xiaogang, X.; Minggui, W. Characterization of fosfomycin resistance gene, *fosB*, in methicillin-resistant *Staphylococcus aureus* isolates. *PLoS ONE*. **2016**, 11, e0154829. doi:10.1371/journal.pone.0154829
28. Rey, M.W.; Ramaiya, P.; Nelson, B.A.; Brody-Karpin, S.D.; Zaretsky, E.J.; Tang, M.; Lopez de Leon, A.; Xiang, H.; Gusti, V.; Clausen, I.G.; Olsen, P.B.; Rasmussen, M.D.; Andersen, J.T.; Jørgensen, P.L.; Larsen, T.S.; Sorokin, A.; Bolotin, A.; Lapidus, A.; Galleron, N.; Ehrlich, S.D.; Berka, R.M. Complete genome sequence of the industrial bacterium *Bacillus licheniformis* and comparisons with closely related *Bacillus* species. *Genome Biol*. **2004**, 5, R77.
29. Barbe, V.; Cruveiller, S.; Kunst, F.; Lenoble, P.; Meurice, G.; Sekowska, A.; Vallenet, D.; Wang, T.; Moszer, I.; Medigue, C.; Danchin, A. From a consortium sequence to a unified sequence: the*Bacillus subtilis* 168 reference genome a decade later. *Microbiology*. **2009**, *155*, 1758-1775. doi:10.1099/mic.0.027839-0.

30. Neyfakh, A.A.; Borsch, C.M.; Kaatz, G.W. Fluoroquinolone resistance protein NorA of *Staphylococcus aureus* is a multidrug efflux transporter. *Antimicrob. Agents Chemother*. **1993**, *37*, 128-129. doi:10.1128/AAC.37.1.128.
31. Doublet, B.; Schwarz, S.; Kehrenberg, C.; Cloeckaert, A. Florfenicol resistance gene *floR* is part of a novel transposon. *Antimicrob. Agents Chemother*. **2005**, *49*, 2106-2108. doi:10.1128/AAC.49.5.2106-2108.2005.
32. Noguchi, N.; Sasatsu, M.; Kono, M. Genetic mapping in *Bacillus subtilis* 168 of the *aadK* gene which encodes aminoglycoside 6-adenylyltransferase. *FEMS Microbiol. Lett*. **1993**, *114*, 47-52.
33. Hosoya, S.; Yamane, K.; Takeuchi, M.; Sato, T. Identification and characterization of the *Bacillus subtilis* D-glucarate/galactarate utilization operon ycbCDEFGHJ. *FEMS Microbiol. Lett*. **2002**, *210*, 193-199.
34. Ohki, R.; Tateno, K.; Takizawa, T.; Aiso, T.; Murata, M. Transcriptional termination control of a novel ABC transporter gene involved in antibiotic resistance in *Bacillus subtilis. J. Bacteriol*. **2005**, *187*, 5946-5954. doi:10.1128/JB.187.17.5946-5954.2005.
35. Duan, J.; Jiang, W.; Cheng, Z.; Heikkila, J.J.;Glick, B.R. The complete genome sequence of the plant growth-promoting bacterium *Pseudomonas* sp. UW4. *PLoS ONE*. **2013**, 8, e58640. doi:10.1371/journal.pone.0058640.
36. Rademacher, C.; Masepohl, B. Copper-responsive gene regulation in bacteria. *Microbiology*. **2012**, *158*, 2451-2464. doi:10.1099/mic.0.058487-0.
37. Zhang, S.; Wang, D.; Wang, Y.; Hasman, H.; Aarestrup, F.M.; Alwathnani, H.A.; Zhu, Y.G.; Rensing, X. Genome sequences of copper resistant and sensitive *Enterococcus faecalis* strains isolated from copper-fed pigs in Denmark. *Stand. Genomic Sci*. **2015**, 10, 35. doi:10.1186/s40793-015-0021-1.
38. Hu, K.H.; Liu, E.; Dean, K.; Gingras, M.; DeGraff, W.; Trun, N.J. Overproduction of three genes leads to camphor resistance and chromosome condensation in *Escherichia coli*. *Genetics*. **1996**, *143*, 1521-1532.
39. Franks, S.E.; Ebrahimi, C.; Hollands, A.; Okumura, C.Y.; Aroian, R.V.; Nizet, V.; McGillivray, S.M. Novel role for the *yceGH* tellurite resistance genes in the pathogenesis of *Bacillus anthracis*. *Infect. Immun*. **2014**, *82*: 1132-1140. doi:10.1128/IAI.01614-13.
40. Moore, C.M.; Gaballa, A.; Hui, M.; Ye, R.W.; Helmann, J.D. Genetic and physiological responses of *Bacillus subtilis* to metal ion stress. *Mol. Microbiol*. **2005**, *57*, 27-40. doi:10.1111/j.1365-2958.2005.04642.x.
41. Osman, D.; Cavet, J.S. Bacterial metal-sensing proteins exemplified by ArsR-SmtB family repressors. *Nat. Prod. Rep*. **2010**, *27*, 668-680. doi:10.1039/b906682a.
42. Wu, X.; Wang, X.; Drlica, K.; Zhao, X. A Toxin-antitoxin module in *Bacillus subtilis* can both mitigate and amplify effects of lethal stress. *PLoS ONE*. **2011**, 6, e23909. doi:10.1371/journal.pone.0023909.
43. Graf, N.; Wenzel, M.; Altenbuchner, J. Identification and characterization of the vanillin dehydrogenase YfmT in *Bacillus subtilis* 3NA. *Appl. Microbiol. Biotechnol*. **2016**, *100*, 3511-3521. doi:10.1007/s00253-015-7197-6.
44. Piddington, C.S.; Kovacevich, B.R.; Rambosek, J. Sequence and molecular characterization of a DNA region encoding the dibenzothiophene desulfurization operon of *Rhodococcus* sp. strain IGTS8. *Appl. Environ. Microbiol*. **1995**, *61*, 468-475.
45. Nguyen, V.D.; Wolf, C.; Mäder, U.; Lalk, M.; Langer, P.; Lindequist, U.; Hecker, M.; Antelmann, H. Transcriptome and proteome analyses in response to 2-methylhydroquinone and 6-brom-2-vinyl-chroman-4-on reveal different degradation systems involved in the catabolism of aromatic compounds in Bacillus subtilis. *Proteomics*. **2007**, *7*, 1391-1408. doi:10.1002/pmic.200700008.
46. Li, L.; Hou, X.; Tsuge, T.; Ding, M.; Aoyama, T.; Oka, A.; Gu, H.; Zhao, Y.; Qu, L. The possible action mechanisms of indole-3-acetic acid methyl ester in Arabidopsis. *Plant Cell Rep*. **2008**, *27*, 575-584. doi:10.1007/s00299-007-0458-9.

47. Lehmann, T.; Hoffmann, M.; Hentrich, M.; Pollmann, S. Indole-3-acetamide-dependent auxin biosynthesis: A widely distributed way of indole-3-acetic acid production? *European J. Cell Biol.* **2010**, *89*, 895-905. doi:10.1016/j.ejcb.2010.06.021.
48. Liu, S.; Ruan, W.; Li, J.; Xu, H.; Wang, J.; Gao, Y.; Wang, J. Biological control of phytopathogenic fungi by fatty acids. *Mycopathologia*. **2008**, *166*, 93-102. doi:10.1007/s11046-008-9124-1.
49. Iang, C.; Yagmin. M.A. Isolation and Anti-phytopathogenic Activity of Secondary metabolites from Alternaria sp. FL25, an Endopytic Fungus in Ficuscarica. *Chinese J. Appl. Environ. Biol*. **2010,** 16(1), 76-78.
50. Mudgal, J.; Rao, C.M.; Arora, D.; Basu Mallik, S.; Pai, K.S.R.; Nampoothiri, M. N-acetyl-L-tryptophan, a substance-P receptor antagonist attenuates aluminum-induced spatial memory deficit in rats AU - Fernandes, Joylee. *Toxicol. Mech. Methods*. **2018,** 28 (5), 328-334.
51. Hawas, W.U.; Al-Farawati, R.; Abou El-Kassem, T.L.; Turki, J.A. Different culture metabolites of the red sea fungus *Fusarium equiseti* optimize the inhibition of hepatitis C virus NS3/4A protease (HCV PR). *Mar. Drugs*. **2016**, 14(10). doi:10.3390/md14100190
52. Sangluo, C., Zhang, D.; Chen, M.; Zhang, Z.; and Lian, X.-Y. A rare diketopiperazine glycoside from marine-sourced Streptomyces sp. ZZ446 AU. *Nat. Prod. Res*. **2018**, 1-5. doi:10.1080/14786419.2018.1544978
53. Inahashi, Y.; Iwatsuki, M.; Ishiyama, A.; Namatame, M.; Nishihara-Tsukashima, A.; Matsumoto, A.; Hirose, T.; Sunazuka, T.; Yamada, H.; Otoguro, K.; Takahashi, Y.; Ōmura, S.; Shiomi, K. Spoxazomicins A–C, novel antitrypanosomal alkaloids produced by an endophytic actinomycete, *Streptosporangium oxazolinicum* K07-0460T. *J. Antibiot*. **2011**, 64, 303.
54. De Carvalho, M.P.; Abraham, W.R. Antimicrobial and biofilm inhibiting diketopiperazines. *Curr. Med. Chem*. **2012**, 19, 3564-3577.
55. Sun, M.; Ou, J.; Li, W.; Lu, C. Quinoline and naphthalene derivatives from *Saccharopolyspora* sp. YIM M13568. *J. Antibiot*. **2017**, 70, 320–322.
56. Xie, C.-L.; Zhou, M.; Xia, M.-L.; Zhou, T.-T.; Chen, H.-F.; Yang, X.-W.; and Yang, Q. Chemical constituents from the deep sea-derived *Streptomyces xiamenensis* MCCC 1A01570 and their effects on RXRα transcriptional regulation. *Nat. Prod. Res*. **2018**, 1-4. doi:10.1080/14786419.2018.150814
57. Fiedler, H.-P.; Bruntner, C.; Bull, A.T.; Ward, A.C.; Goodfellow, M.; Potterat, O.; Puder, C.; Mihm, G. Marine actinomycetes as a source of novel secondary metabolites. *Antonie Leeuwenhoek*. **2005**, 87(1), 37-42.
58. Cho, JY.; Kang, J.Y.; Hong, Y.K.; Baek, H.H.; Shin, H.W.; Kim, M.S. Isolation and structural determination of the antifouling diketopiperazines from marine-derived *Streptomyces praecox* 291-11. *Biosci. Biotechnol. Biochem*. **2012**, 76(6), 1116-1121.
59. Ye, X.; Chai, W.; Lian, X.-Y.; Zhang, Z. Novel propanamide analogue and antiproliferative diketopiperazines from mangrove Streptomyces sp. Q24. *Nat. Prod. Res*. **2017**, 31(12), 1390-1396. doi: 10.1080/14786419.2016.1253079.
60. Ingram, K.; Ellis, W.; and Keiser, J. Antischistosomal activities of mefloquine-related arylmethanols. *Antimicrob. Agents Chemother*. **2012**, 56(6), 3207.
61. Majik, M.S.; Tilve, S.G. Pyrrolizidine alkaloids pyrrolams A–D: A survey of synthetic efforts, biological activity, and studies on their stability. *Synthesis*. **2012**, 44(17), 2673-2681.
62. El-Gendy, B.E.D.M.; Rateb, M.E. Antibacterial activity of diketopiperazines isolated from a marine fungus using t-butoxycarbonyl group as a simple tool for purification. *Bioorg. Med. Chem. Lett*. **2015**, 25(16), 3125-3128.
63. Huang, S.-X.; Powell, E.; Rajski, S.R.; Zhao, L.-X.; Jiang, C.-L., Duan, Y.; Xu, W.; Shen, B. Discovery and total synthesis of a new estrogen receptor heterodimerizing Actinopolymorphol A from Actinopolymorpharutilus. *Org. Lett*. **2010**, 12(15), 3525-3527.
64. Pérez-Picaso, L.; Olivo, H.F.; Argotte-Ramos, R.; Rodríguez-Gutiérrez, M.; Rios, M.Y. Linear and cyclic dipeptides with antimalarial activity. *Bioorg. Med. Chem. Lett*. **2012**, *22(23)*, 7048-7051.

65. Cao, D.D.; Do, T.Q.; Doan Thi Mai, H.; Vu Thi, Q.; Nguyen, M.A.; Le Thi, H.M.; Tran, D.T.; Chau, V.M.; Cong Thung, D.; Pham, V.C. Antimicrobial lavandulylated flavonoids from a sponge-derived actinomycete. *Nat. Prod. Res*. **2018**, 1-8. doi:10.1080/14786419.2018.1538219.
66. Xingliang, L.; Xiang, Z.; Min, Z.; Qinglai, W.; Xudong, Z.; Junkai, L. Synthesis and fungicidal activity of 1,3,4-oxadiazol-2-yl thioether derivatives containing a phenazine-1-carboxylic acid scaffold. *Nat. Prod. Res*. **2018**, 1-6. doi:10.1080/14786419.2018.1489389.
67. Audisio, D.; Messaoudi, S.; Cojean, S.; Peyrat, J.-F.; Brion, J.-D.; Bories, C.; Huteau, F.; Loiseau, P.M.; Alami, M. Synthesis and antikinetoplastid activities of 3-substituted quinolinones derivatives. *European J. Med. Chem***. 2012**, *52*, 44-50.
68. Mashburn-Warren, L.; Howe, J.; Brandenburg, K.; Whiteley, M. Structural requirements of the *Pseudomonas* quinolone signal for membrane vesicle stimulation. *J. Bacteriol***. 2009**, *191(10)*, 3411.
69. Degrassi, G.; Aguilar, C.; Bosco, M.; Zahariev, S.; Pongor, S.; Venturi, V. Plant growth-promoting *Pseudomonas putida* WCS358 produces and secretes four cyclic dipeptides: Cross-Talk with quorum sensing bacterial sensors. *Curr. Microbiol***. 2002**, 45, 250-254.
70. Marti, T.; Hu, Z.; Pohl, N. L.; Shah, A. N.; Khosla, C. Cloning, nucleotide sequence, and heterologous expression of the biosynthetic gene cluster for R1128, a non-steroidal estrogen receptor antagonist: Insights into an unusual priming mechanism. *J. Biol. Chem***. 2000**, *275(43)*, 33443-33448.
71. Sivaprakasam, S.; Bhutia, D.Y.; Ramachandran, S.; Ganapathy, V. Cell-surface and nuclear receptors in the colon as targets for bacterial metabolites and its relevance to colon health. *Nutrients*. **2017**, *9(8)*, 856. doi:10.3390/nu9080856.
72. Uchida, R.; Imasato, R.; Yamaguchi, Y.; Masuma, R.; Shiomi, K.; Tomoda, H.; Ōmura, S. Yaequinolones, new insecticidal antibiotics produced by *Penicillium* sp. FKI-2140. *J. Antibiotics*. **2006**, *59*, 646-651. doi:10.1038/ja.2006.86.
73. He, R.; Wang, B.; Wakimoto, T.; Wang, M.; Zhu, L.; Abe, I. Cyclodipeptides from metagenomic library of a Japanese marine sponge. *J. Braz. Chem. Soc*. **2013,** *24*, 1926-1932.
74. Furukawa, T.; Akutagawa, T.; Funatani, H.; Uchida, T.; Hotta, Y.; Niwa, M.; Takaya, Y. Cyclic dipeptides exhibit potency for scavenging radicals. *Bioorg. Med. Chem. Lett*. **2012**, *20(6)*, 2002-2009.
75. Bukovits, G.; Mohr, N.; Budzikiewicz, H. 2-Phenylthiazol-derivate aus *Pseudomonas* cepacia/2-Phenylthiazole derivatives from *Pseudomonas cepacia*. Zeitschrift für Naturforschung B. **1982**, *37(7)*, 877-880. doi:10.1515/znb-1982-0717.
76. Shapiro, J.A.; Morrison, K.R.; Chodisetty, S.S.; Musaev, D.G.; Wuest, W.M. Biologically inspired total synthesis of Ulbactin F, an iron-binding natural product. *Org. Lett*. **2018**, *20(18)*, 5922-5926.
77. Patil, R.; Patil, S.A.; Beaman, K.D.; Patil, S.A. Indole molecules as inhibitors of tubulin polymerization: potential new anticancer agents, an update (2013–2015). *Future Med. Chem***. 2016**, 8(11), 1291-1316.
78. Walchshofer, N.; Sarciron, M.E.; Garnier, F.; Delatour, P.; Petavy, A.F.; Paris, J. Anthelmintic activity of 3, 6-dibenzyl-2,5-dioxopiperazine, cyclo(L-Phe-L-Phe). *Amino Acids*. **1997**, *12(1)*, 41-47.
79. Reil, E.; Höfle, G.; Draber, W.; Oettmeier, W. Quinolones and their N-oxides as inhibitors of photosystem II and the cytochrome b6/f-complex. *Biochim. Biophys. Acta Bioenerg*. **2001**, 1506(2), 127-132.
80. Kitamura, S.; Hashizume, K.; Iida, K.; Miyashita, E.; Shirahata, K.; Kase, H. Studies on lipoxygenase inhibitors. II. KF8940 (2-n-heptyl-4-hydroxyquinoline-n-oxide), A potent and selective inhibitor of 5-lipoxygenase, produced by pseudomonas methanica. *J. Antibiot*. **1986**, 39(8), 1160-1166.
81. Shin, C.; Lim, H.; Moon, S.; Kim, S.; Yong, Y.; Kim, B. J.; Lee, C. H.; Lim, Y. A novel antiproliferative agent, phenylpyridineylbutenol, isolated from *Streptomyces* sp. *Bioorg. Med. Chem. Lett*. **2006**, 16(21), 5643-5645.

82. Funaki, M.; Tsuchiya, F.; Maeda, K.; Kamiya, T. Cyanomycin, a new antibiotic. *J. Antibiot*. **1958**, 11(4), 143-149.
83. Feng, N.; Ye, W.; Wu, P.; Huang, Y.; Xie, H.; Wei, X. Two new antifungal alkaloids produced by *Streptoverticillium morookaense*. *J. Antibiot*. **2007**, *60(3)*, 179-83.
84. Seo, B., Yun, J., Lee, S., Kim., M., Hwang, K., Kim, J., Min, K.R., Kim, Y., Moon, D. Barbarin as a new tyrosinase inhibitor from *Barbarea orthocerus*. *Planta Medica*. **1999**, *65(8)*, 683-685.
85. Brandel, J.; Humbert, N.; Elhabiri, M.; Schalk, I.J.; Mislin, G.L.; Albrecht-Gary, A.M. Pyochelin, a siderophore of *Pseudomonas aeruginosa*: physicochemical characterization of the iron (III), copper (II) and zinc (II) complexes. *Dalton Trans*. **2012**, 7, 2820-2834. doi:10.1039/c1dt11804h.
86. Sumoski, W.; Baquerizo, H.; Rabinovitch, A. Oxygen free radical scavengers protect rat islet cells from damage by cytokines. *Diabetologia*. **1989**, *32(11)*, 792-796.
87. Capon, R.J.; Skene, C.; Lacey, E.; Gill, J.H.; Wicker, J.; Heiland, K.; Friedel, T. Lorneamides A and B: Two new aromatic amides from a southern Australian marine actinomycete. *J. Nat. Prod*. **2000**, *63*, 1682-1683.
88. Sobolevskaya, M.P.; Kuznetsova, T.A. Biologically active metabolites of marine actinobacteria. *Russ. J. Bioorganic Chem*. **2010**, *36*, 560-573.
89. Lai, D.; Brötz-Oesterhelt, H.; Müller, W. E. G.; Wray, V.; Proksch, P. Bioactive polyketides and alkaloids from *Penicillium citrinum*, a fungal endophyte isolated from *Ocimum tenuiflorum*. *Fitoterapia*. **2013**, *91*, 100-106.
90. Umezawa, S.; Tsuchiya, T.; Tatsuta, K.; Horiuchi, Y.; Usui, T. A new antibiotic, dienomycin. I. Screening method, isolation and chemical studies. *J. Antibiot*. **1970,** *23(1)*, 20-27.
91. Supong, K.; Thawai, C.; Supothina, S.; Auncharoen, P.; Pittayakhajonwut, P. Antimicrobial and anti-oxidant activities of quinoline alkaloids from *Pseudomonas aeruginosa* BCC76810. *Phytochem. Lett*. **2016**, 17, 100-106.
92. Krab, K.; Wikstrom, M. Effect of 2-n-Heptyl-4-hydroxyquinoline N-oxide on proton permeability of the mitochondrial membrane. *Biochem. J*. **1980**, *186*, 637-639.
93. O'Donnell, F.; Smyth, T.J.P.; Ramachandran, V.N.; Smyth, W.F. A study of the antimicrobial activity of selected synthetic and naturally occurring quinolines. *Int. J. Antimicrob. Agents*. **2010**, *35(1)*, 30-38.
94. Audisio, D.; Messaoudi, S.; Cojean, S.; Peyrat, J.F.; Brion, J.D.; Bories, C.; Huteau, F.; Loiseau, P.M.; Alami, M. Synthesis and antikinetoplastid activities of 3-substituted quinolinones derivatives. *European J. Med. Chem*. **2012**, 52, 44.
95. Malipeddi, H.; Jose, T.; Mv Ramana, D.; Anand, R. Synthesis and biological evaluation of indole-3-carboxylic acid derivatives of amino acids and peptides. *Int. Res. J. Pharm*. **2010**, *1(1)*, 436-440.
96. Khan, I.; Shah, S. J.A.; Ejaz, S.A.; Ibrar, A.; Hameed, S.; Lecka, J.; Millán, J.L.; Sévigny, J.; Iqbal, J. Investigation of quinoline-4-carboxylic acid as a highly potent scaffold for the development of alkaline phosphatase inhibitors: synthesis, SAR analysis and molecular modelling studies. RSC Advances. **2015,** *5(79)*, 64404-64413.
97. Pesci, E.C.; Milbank, J.B.J.; Pearson, J.P.; McKnight, S.; Kende, A.S.; Greenberg, E.P.; Iglewski, B.H. Quinolone signaling in the cell-to-cell communication system of Pseudomonas aeruginosa. *Proc. Natl. Acad. Sci. USA*. **1999**, 96, 11229-11234.
98. Teruya, T.; Kobayashi, K.; Suenaga, K.; Kigoshi, H. Phormidinines A and B, novel 2-alkylpyridine alkaloids from the cyanobacterium *Phormidium* sp. *Tetrahedron Lett*. **2005**, *46(23)*, 4001-4003.
99. Lee, C.H.; Lee, H.S. Relaxant effect of quinoline derivatives on histamine-induced contraction of the isolated guinea pig trachea. *J. Korean Soc. Appl. Biol. Chem*. **2001**, 54(1), 118-123.
100. Rocha, T.M.; Machado, N.J.; de Sousa, J.A.C.; Araujo, E.V.O.; Guimaraes, M.A.; Lima, D.F.; Leite, J.R.D.S.D.A.; Leal, L.K.A.M. Imidazole alkaloids inhibit the pro-inflammatory mechanisms of human neutrophil and exhibit anti-inflammatory properties *in vivo*. *J. Pharm. Pharmacol*. **2019**. doi:10.1111/jphp.13068

101. Mori, T.; Yamashita, T.; Furihata, K.; Nagai, K.; Suzuki, K.I.; Hayakawa, Y.; Shin-Ya, K. Burkholone, a new cytotoxic antibiotic against IGF-I dependent cells from *Burkholderia* sp. *J. Antibiot*. **2007**, *60(11)*, 713-716.
102. Needham, J.; Andersen, R.J.; Kelly, M.T. Oncorhyncolide, a novel metabolite of a bacterium isolated from seawater. *Tetrahedron Lett*. **1991**, *32(3)*, 315-318.
103. Cho, J.Y. Algicidal activity of marine *Alteromonas* sp. KNS-16 and isolation of active compounds. *Biosci. Biotechnol. Biochem*. **2012**, *76(8)*, 1452-1458.
104. Shi, H.; Liu, W.; Wei, Y.; Ye, T. Integration of auxin/indole-3-acetic acid 17 and RGA-LIKE3 confers salt stress resistance through stabilization by nitric oxide in Arabidopsis. *J. Exp. Bot*. **2017**. *68(5)*, 1239-1249. doi: 10.1093/jxb/erw508.
105. Duarte, B.; Matos, A.R.; Marques, J.C.; Caçador, I. Leaf fatty acid remodeling in the salt-excreting halophytic grass Spartina patens along a salinity gradient. *Plant Physiol. Biochem*. **2018**, *124*, 112-116. doi: 10.1016/j.plaphy.2018.01.007.
106. Aziz, A.; Siti-Fairuz, M.; Abdullah, M.Z.; Ma, N.L.; Marziah, M. Fatty acid profile of salinity tolerant rice genotypes grown on saline soil. *Malays. Appl. Biol*. **2015**, *44*, 119-124.
107. Yang, Y.; Yan, L; Yana, S; Tao, L; Yanchun, C; Dake, Z; Zhiwei, Z. The Role of phyto-melatonin and related metabolites in response to stress. Molecules. **2018**, *23(8)*, 1887. doi: 10.3390/molecules23081887.
108. Jia, H.; Shao, M.; He, Y.; Guan, R.; Chu, P.; Jiang, H. Proteome dynamics and physiological responses to short-term salt stress in *Brassica napus* leaves. PLoS One. **2015**, 10(12), e0144808. doi: 10.1371/journal.pone.0144808.
